# Surface coating and speckling of the human iliotibial tract does not affect its load-deformation properties

**DOI:** 10.1038/s41598-020-77299-1

**Published:** 2020-11-27

**Authors:** Johann Zwirner, Benjamin Ondruschka, Mario Scholze, Niels Hammer

**Affiliations:** 1 Department of Anatomy, University of Otago, Dunedin, New Zealand; 2 Institute of Legal Medicine, University Medical Center Hamburg-Eppendorf, Hamburg, Germany; 3 Institute of Legal Medicine, University of Leipzig, Leipzig, Germany; 4 Institute of Materials Science and Engineering, Chemnitz University of Technology, Chemnitz, Germany; 5 Department of Macroscopic and Clinical Anatomy, Medical University of Graz, Graz, Austria; 6 Department of Orthopaedic and Trauma Surgery, University of Leipzig, Leipzig, Germany; 7 Fraunhofer IWU, Dresden, Germany

**Keywords:** Biomedical engineering, Ligaments

## Abstract

Stochastic surface patterns form an important requirement to facilitate digital image correlation and to subsequently quantify material properties of various tissues when loaded and deformed without artefacts arising from material slippage. Depending on the samples’ natural colour, a surface pattern is created by speckling with colour or dye only, or it requires combined surface coating and speckling before to enhance the contrast, to facilitate high-quality data recording for mechanical evaluation. However, it is unclear to date if the colours deployed for coating and speckling do significantly alter the biomechanical properties of soft tissues. The given study investigated the biomechanical properties of 168 human iliotibial tract samples as a model for collagen-rich soft tissues, separated into four groups: untreated, graphite speckling only, water-based coating plus graphite speckling and solvent-based coating plus graphite speckling following a standardized approach of application and data acquisition. The results reveal that elastic modulus, ultimate tensile strength and strain at maximum force of all groups were similar and statistically non-different (p ≥ 0.69). Qualitatively, the speckle patterns revealed increasing contrast differences in the following order: untreated, graphite speckling only, water-based coating plus graphite speckling and solvent-based coating plus graphite speckling. Conclusively, both coating by water- and solvent-based paints, as well as exclusive graphite speckling, did not significantly influence the load-deformation parameters of the here used human iliotibial tract as a model for collagen-rich soft tissues. In consequence, water- and solvent-based coating paints seem equally suitable to coat collagen-rich soft tissues for digital image correlation, resulting in suitable speckle patterns and unbiased data acquisition.

## Introduction

Digital image correlation (DIC) is an established method in experimental biomechanics to obtain load-deformation properties such as elastic modulus (E_mod_), ultimate tensile strength (UTS) or strain at maximum force (SF_max_) of biological tissues based on surface deformation when the tissues are loaded and subsequently deformed^1–5^. In the case of non-homogenous and anisotropic materials, DIC enables for accurate contactless measurements at a reasonable cost-benefit ratio compared to other techniques such as holographic interferometry, electronic speckle pattern interferometry or Moiré interferometry^6^. Software using the DIC data helps calculate in-surface strain fields by tracking sub-images (facets) of the entire digital image, which are represented by grey-level distributions of a random pattern^7,8^. If the sample does not have a natural stochastic surface pattern, creating a high-contrast surface pattern forms a crucial step of the sample preparation for the subsequent biomechanical experiments^9,10^. Commonly, the speckle patterns are sprayed on the samples using a high-contrast paint or a dispersion of graphite powder^9,10^. Most frequently, a black-on-white speckle pattern with black speckles on a white-coated background is created, especially if the specimens are too dry to assure a proper attachment of the graphite and if the natural surface colour fails to create a sufficient contrast to the added speckles^11^. It is to date unclear whether and to what extent surface treatments of human collagen-rich soft tissues to create stochastic speckle patterns alter their biomechanical properties as a measurement setup-related factor. If surface treatments of collagen-rich soft tissues such as coating and speckling are destructive to collagens as the predominant load-bearing extracellular matrix components^12^, a decrease of the respective UTS of the sample compared to the untreated sample can be expected. Recently, it was shown that the E_mod_ of the human iliotibial tract critically depends on its hydration state^13^. This highlights not only the importance to osmotically adapt all tested samples when biomechanical properties are obtained in vitro^14,15^, but also that additional over- or dehydrating of the tested samples should be avoided to the best possible extent. However, some of the chemicals, colours and dyes to create speckle patterns include dehydrating components such as acetone^16^ or isopropanol^17^ with a potential influence on the obtained biomechanical properties of the tested tissues. In a previous study, our group found that commercially available solvent- and water-based sprays did not alter the mechanical properties of rat bones when tested under quasi-static conditions^1^. The question arises, whether these observations on hard tissues can be extrapolated to soft tissues.

The given study aims to assess the influence of coating and speckling on the load-deformation properties of the human iliotibial tract as a well-characterized model of collagen-rich human soft tissues^18–22^. Based on the dehydrating components of coating sprays and the previous observations on hard tissues, we investigated the two following hypotheses:

1. The load-deformation properties of the human iliotibial tract are altered by coating sprays.
2. The load-deformation properties of the human iliotibial tract remain unaltered by graphite speckling.

## Materials and methods

### Harvesting and sample preparation for mechanical testing

A total of 24 iliotibial tract specimens from 22 cadavers (5 female, 17 male, mean age 47 ± 23 years) were retrieved during forensic autopsies at the Institute of Legal Medicine, University of Leipzig, Germany. All specimens were stored deep-frozen at − 80 °C until mechanical testing was performed, following an initial precooling step. When further processed, the specimens were thawed and cut into 2 to 17 small subsamples, depending on the initial size of the iliotibial tract. The sample preparation for mechanical testing including the application of an osmotic stress protocol was performed as described previously^4,19^. The study has been approved by the Ethics Committee of the University of Leipzig, Germany (protocol number 486/16-ek) and is in line with the Saxonian Death and Funeral Act of 1994 (third section, paragraph 18 item 8). In line with German law, the state attorney as the legal representative was informed about the use of the tissues for research purposes in each case.

### Coating and speckling for digital image correlation

**Figure 1:**
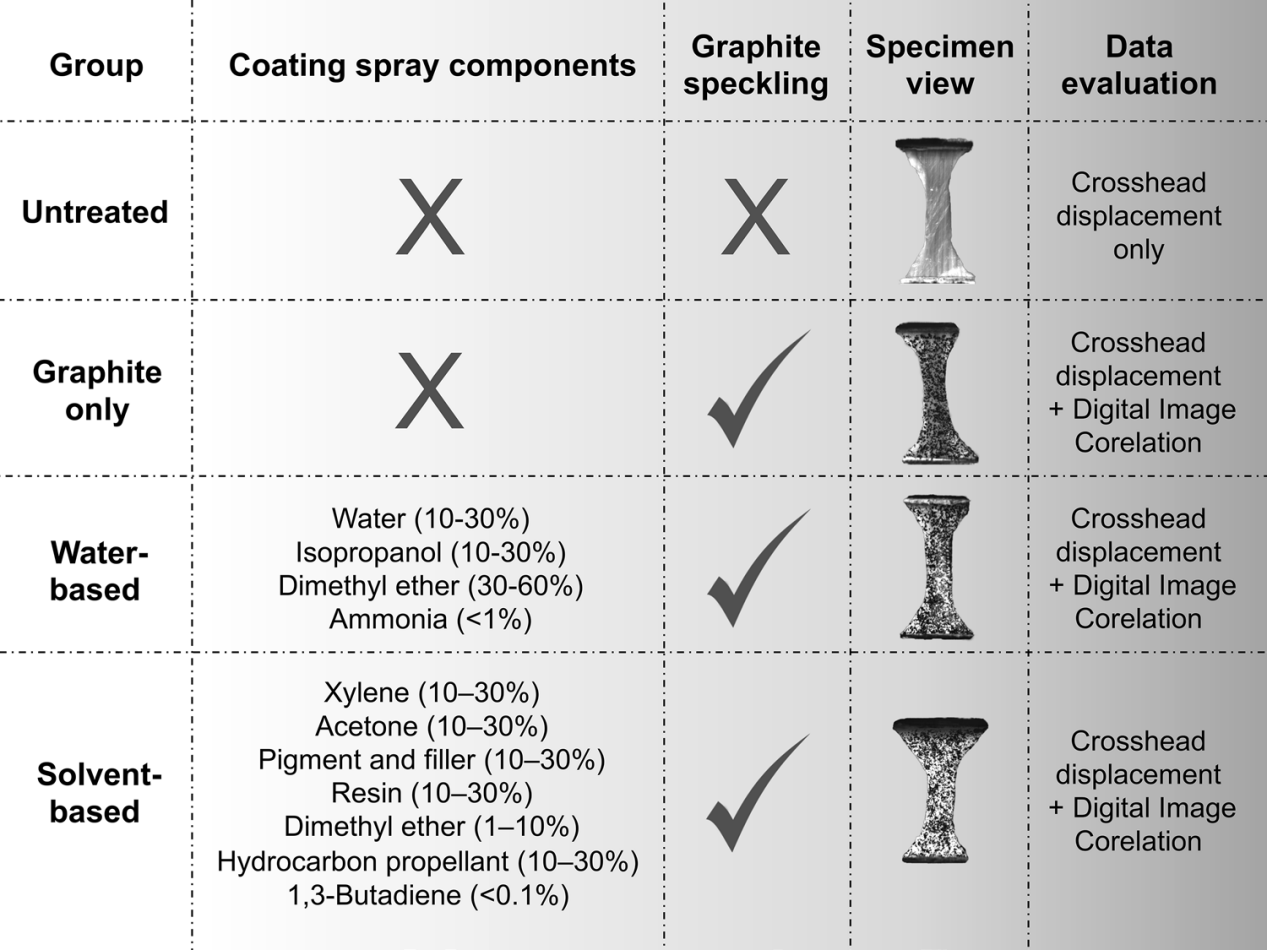
This figure depicts the individual preparation of the four different groups for the mechanical tests and whether crosshead displacement data or digital imaging correlation data was evaluated. Special symbols: X, no coating/speckling performed; ✓, speckling performed.

Before the tensile testing, the iliotibial tract samples were allocated into four different groups. The first group of 26 samples was left uncoated and without speckles and is subsequently referred to as the ‘untreated group’. In group number two, consisting of 48 samples, a speckle pattern using graphite powder was created (‘graphite only group’). The 50 samples of group number three were coated using a white water-based spray (Dy-Mark; Spray & Mark aerosol paint; Wacol, QLD, Australia) before the graphite speckling pattern was created (‘water-based group’). The last group of 44 samples was coated using a white solvent-based spray (Dy-Mark) before the speckle pattern creation with graphite powder (‘solvent-based group’). Spraying and speckling was done as stated previously^1^. The samples were mechanically tested within approximately three minutes after they had been spray-coated. Figure 1 summarizes the treatment of the four different groups before mechanical testing.

### Uniaxial quasi-static mechanical testing

**Figure 2:**
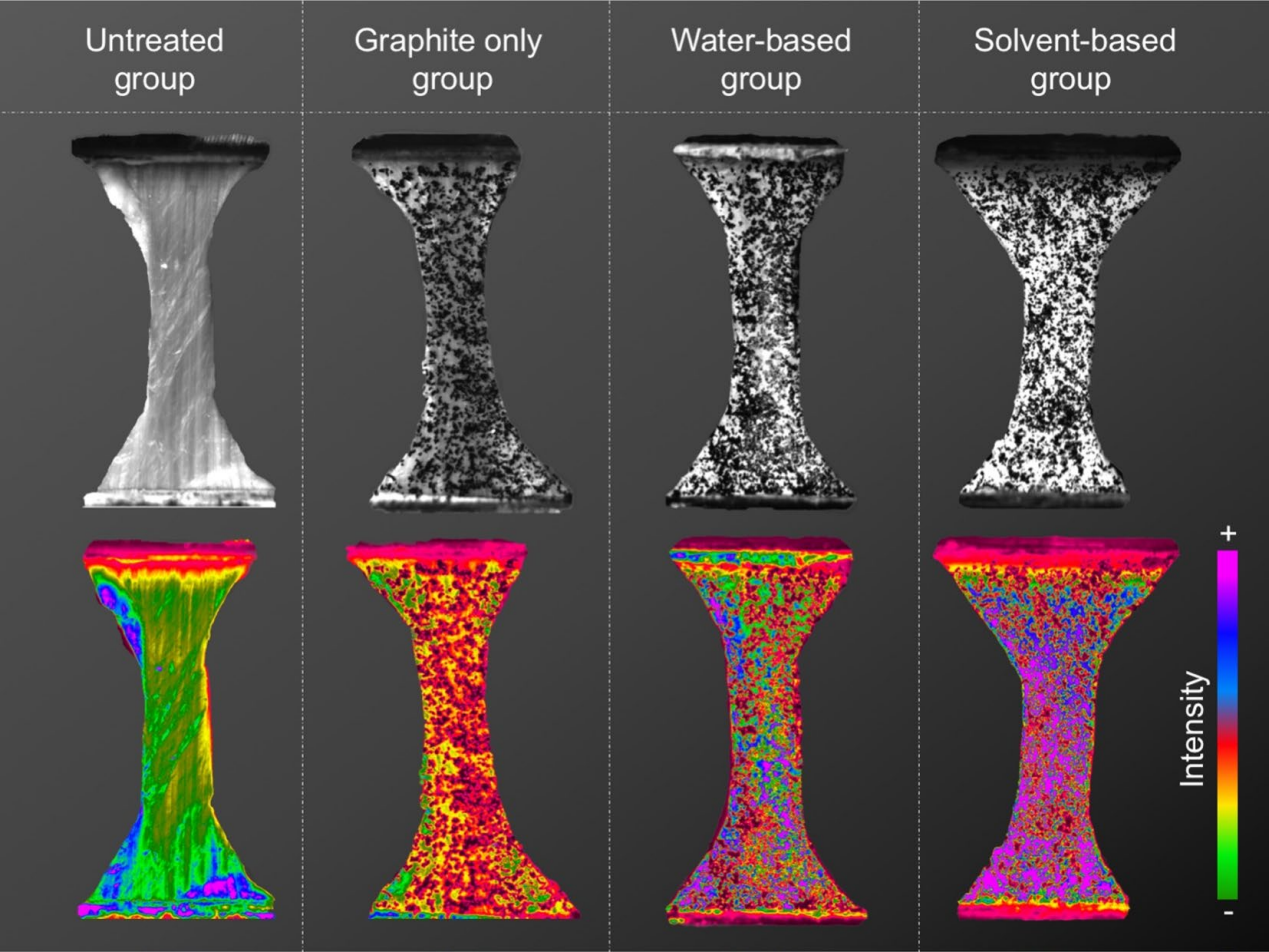
Images of representative samples of the four different groups in this study. Each sample is depicted as seen with the naked eye (upper row) and when using the Istra mode of the digital image correlation (lower row), which uses a special colour table to highlight intensity variations.

The cross-sectional areas of the samples were determined by the creation of polysiloxane (medium-bodied, Exahiflex; GC Corporation, Tokyo, Japan) cross-section casts, which were subsequently scanned (Perfection 7V750Pro; Seiko Epson Corporation, Suwa, Japan) and computed using the Measure 2.1d software (DatInf, Tübingen, Germany). Self-3D-printed clamps composed of polylactic acid were used as described previously^8^. A uniaxial testing machine (Allround Table Top Z020; Zwick Roell, Ulm, Germany) with an Xforce P load cell (2.5 kN; Zwick Roell) was used to perform the tensile tests at room temperature. The testControl II software (Zwick Roell) was used. Before the samples were stretched until material failure occurred, twenty load-unload preconditioning cycles with a force range of 0.5–2.0 N were applied. All tissues were strained in the longitudinal axis according to the samples’ predominant collagen orientation as can be observed in the untreated sample in Fig. 2 (leftmost image top row). The displacement rate was 20 mm/min (initial strain rate of 0.03 s^−1^) with a sampling rate of 100 Hz. A single-charge coupled camera (Q400, 2.8 Megapixels; Limess, Krefeld, Germany) and the ISTRA 4D software (VRS 4.4.1.354; Dantec Dynamics, Skovlunde, Denmark) were used for strain data evaluation of the mechanical tests. Using the “Istra” mode of the ISTRA 4D software, the greyscale was converted into pseudo colours of a “rainbow” colour map, similar to the Scilab programming language (ESI group, Orsay, France).

### Data processing and statistical analysis

Synchronized force readings combined with the DIC were used to calculate engineering stress-strain curves using MATLAB R2017b software (Mathworks, Natick, MA, USA) for the graphite only, the water-based and the solvent-based group. The crosshead displacement data obtained via the testControl II software were used to calculate mechanical parameters for all tested groups. The elastic modulus (E_mod_) was evaluated in the linear region of each stress-strain graph, ultimate tensile stress (UTS) corresponds to the maximum stress of each graph and strain at maximum force (SF_max_) to the engineering strain value when UTS/F_max_ is reached. For statistical evaluation, Excel version 16.15 (Microsoft Corporation, Redmond, WA, USA) and GraphPad Prism version 7 (GraphPad Software, La Jolla, CA, USA) were deployed. A Kruskal-Wallis test followed by an uncorrected Dunn’s test was applied. Mean values ± standard errors of the mean are reported in the text. The outlines of the boxes in the box plots indicate the 25th and 75th, Whiskers the 10th and 90th percentile, the solid black horizontal line the median. Outliers (values below the 10th or higher than the 90th percentile) are depicted as small black circles. For all statistical tests in this study, *p*-values equal to or smaller than 0.05 were considered significant.

## Results

### Surface coating does not affect quasi-static load-deformation properties of the human iliotibial tract

**Figure 3:**
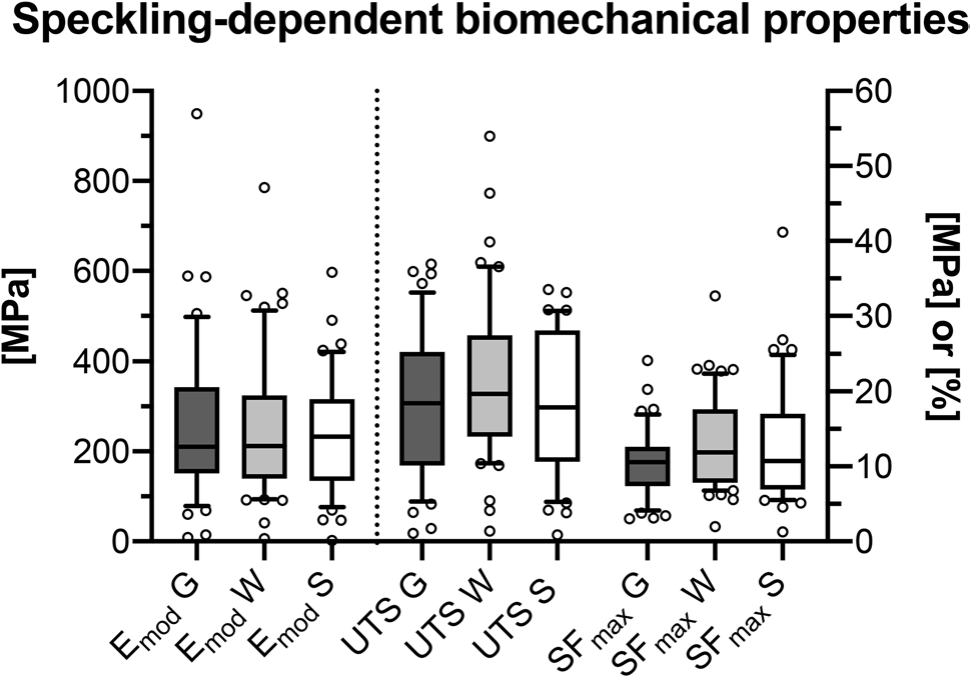
The digital image correlation based biomechanical parameters are depicted separated for the three different speckling methods. The dotted line separates left and right y-axis. E_mod_, elastic modulus [in MPa]; UTS, ultimate tensile strength [in MPa]; SFmax [in %], strain at maximum force; G, graphite speckling only; W, water-based coating (+ graphite speckling); S, solvent-based coating (+ graphite speckling).

The E_mod_ of only graphite speckled samples of 258 ± 25 MPa (median: 210 MPa) was similar and statistically non-different from both the E_mod_ of the water-based coated samples of 249 ± 22 MPa (median: 202 MPa, *p* = 0.99) and the solvent-based coating group of 240 ± 20 MPa (median: 233 MPa, *p* = 0.99). Equally, the elastic moduli of the two different coating groups were statistically non-different (*p* = 0.99). The groups were similar regarding their UTS with 19 ± 1 MPa (median: 19 MPa) for the graphite group, 21 ± 1 MPa (median: 20 MPa) for the water-based group and 19 ± 1 MPa (median: 18 MPa) for the solvent-based group (graphite-water coating: *p* = 0.43, graphite-solvent coating: *p* = 0.99, water coating-solvent coating: *p* = 0.43). Also, the SF_max_ of the graphite group of 11 ± 1% (median: 11%), the water-based group of 13 ± 1% (median: 12%) and the solvent-based group with 13 ± 2% (median: 11%) were similar and statistically non-different (graphite-water coating: *p* = 0.30, graphite-solvent coating: *p* = 0.55, water coating-solvent coating: *p* = 0.69). Figure 3 graphically summarises the DIC-based biomechanical parameters.

### Crosshead displacement data showed that graphite coating does not influence the biomechanical parameters of the iliotibial tract when loaded quasi-statically

The machine-based biomechanical parameters revealed that the E_mod_ of the untreated group was comparable and statistically non-different to the graphite only group (*p* = 0.54), the water-based coated group (*p* = 0.55) as well as the solvent-based coated group (*p* = 0.60). Likewise, the UTS of the untreated group did not statistically differ from the other three groups (graphite only group: *p* = 0.96, water-based group: *p* = 0.65, solvent-based group: *p* = 0.70). Also, the SF_max_ values of the untreated group were similar and statistically non-different compared to the graphite group (*p* = 0.50), the water-based group (*p* = 0.15) and the solvent-based group (*p* = 0.31).

### The features of the speckling differed qualitatively in optical intensity for the four coating groups

Qualitatively, the four groups investigated here differed regarding the occurrence of high-intensity areas throughout the entire tested sample. The occurrence of high-contrast areas increased in the following order (lowest first): untreated, graphite speckling only, water-based coating with graphite speckling and solvent-based coating with graphite speckling.

## Discussion

The DIC-based deformation quantification allows calculating biomechanical parameters of tissues based on surface deformations in a contactless manner^6^ at high accuracy. Thereby, DIC obviates test device-related inaccuracies such as material slippage^23^ or the influence of the test devices’ own elasticity^1^. At the same time, the DIC software requires the recognition of a stochastic pattern with different grey values on the samples’ surface, which might be obtained by the creation of a speckle pattern only or in cases of low contrasts the preceding coating of the surface using an oppositional colour compared to the speckles. Artificial speckling patterns have been applied to various human soft tissues to enable the use of DIC for the determination of mechanical parameters before, including the aorta^24^, dura mater^4^, the hip capsule^14^ iliotibial tract^18,19,23^, sclera^25^ and temporal muscle^3^ or the vocal ligament^26^. However, the influence of different speckle techniques on the biomechanical properties of soft tissues was scarcely studied to date. Thus it remained unclear if osmotic changes or chemical denaturation resulting from the colours and dyes may have significantly affected the tissue mechanics. Studies so far exclusively investigated animal soft tissues such as porcine ligaments^27^ or murine arteries^28^. Moreover, previous studies did not independently investigate the effects of several different components to create the speckle pattern such as the simultaneous use of coating sprays and graphite particles. The former provided the rationale for the two hypotheses stated above.

This study for the first time confirmed that the load-deformation parameters of the iliotibial tract as a well-investigated and representative model for collagen-rich human soft tissues remain unaffected by the use of water- and solvent-based coating sprays. Therefore, the first hypothesis must be rejected and it is stated that a potential dehydrating or damaging effect of coating sprays is statistically insignificant regarding their biomechanical properties. To avoid dehydration of the samples to the best possible extent, each sample was coated and subsequently speckled immediately before the tensile tests. From the authors' own experience, the typical time frame between the first contact of the coating spray with the sample and the conclusion of the tensile test can be accomplished in less than three minutes. According to the manufacturer the drying time of both used sprays in this study is five minutes, the coating most likely had not been dried completely by the end of the here performed mechanical test. For biomechanical tests as conducted in this study, a moist coating does provide two advantages: Firstly, the obtained results reveal that the biomechanical parameters of the here tested iliotibial tract were not influenced by the wet coating, which may be different after the coating dried completely. Secondly, the moist coating surface provides a sticky adhesion surface for the graphite particles^6^, assuring the speckle pattern with abundant areas of high contrast differences as seen in this study. Even during the quasi-static tensile tests with continuous deformation of the samples, the undried coatings in this study provided stable patterns that remained on all tested samples until the respective test had been finished allowing for an uncomplicated DIC analysis in every individual case.

According to the manufacturer's datasheet^1,^ the water-based coating spray used in this study consists of 10–30% water, 10–30% isopropanol, 30–60% dimethyl ether and less than 1% ammonia. Isopropanol is a drying agent that extracts water between the tropocollagen molecules leading to a collapse of the hydrogen bonds hampering the collagen fibril rearrangement hence causing an increased stiffness^17^. The propellant dimethyl ether dissolves fat^29^, which has to be considered when adipose surfaces are coated. The solvent-based coating spray contains acetone, hydrocarbon propellant, pigment/filler, resin, xylene (all 10–30%), 1–10% dimethyl ether and less than 0.1% 1,3-butadiene. Despite having a dehydrating effect on collagens, even in high acetone concentrations, the collagens were found to remain intact^30^. In short, two effects of the here used coating sprays can be crystallized: specimen damage and dehydration. As the here tested iliotibial tract is mainly composed of collagens^19^ potential specimen damage following the dissolving of adipose tissue by the coating can be neglected in the here performed study. Even though not investigated microscopically, the collagens in this study seemed to be unaffected by the speckling from a biomechanical point of view as the UTS was statistically non-different in coated compared to uncoated samples. Another important aspect is that the averaged 12 ± 6 μm-thick coating^31^ that is created by experienced DIC users is only present on the surface of the sample. Therefore, aggressive coating components most likely affect the most superficial collagen layers rather than covering all load-bearing sample components at the same time. Even in the unlikely event that this superficial layer was damaged by water- or solvent-based coatings, this was shown to be biomechanically negligible when compared to the untreated state in this given study. Regarding the potential dehydrating effect of the used coating sprays, it can be concluded that the contact time of the used coatings with the sample has been too short to biomechanically reflect signs of dehydration such as an increased E_mod_^13^. Moreover, in the here presented experiments the portion of the dehydrating component might have been below a certain threshold before a dehydration is reflected in the here obtained biomechanical parameters. Also, hydrating spray components such as water or hydrocarbon, might have counterbalanced the dehydrating effect of acetone or isopropanol. With regards to the potential impact of solvents on the biomechanical properties of the soft tissue sample, it was suggested to use water-based paints instead of solvent-based ones^6^. This study reveals that both paints can be applied to the human iliotibial tract and, therefore, potentially other collagen-rich soft tissues as neither of them altered the biomechanical properties of the here tested samples. Even though not quantified, the here used white solvent-based paint seemed to create larger contrast differences to the grey graphite speckle particles compared to the white water-based paint and might be preferred especially when the illumination of the tested area is low.

As the mechanical parameters were independent of graphite speckling the second hypothesis can be accepted. Graphite powder only seems the best suited of the three speckling methods for biomechanical tests, as it is inexpensive, provides a high contrast on bright surfaces due to its grey colour and interacts with the sample in a way that can be neglected from a biomechanical point of view. Graphite is insoluble^32^ and therefore does not diminish on moist surfaces during the performed mechanical tests. The attachment of the graphite particles to the sample’s surface is chemically explained by the attraction between graphite and water. This given study revealed that bright and moist surfaces such as the one of the iliotibial tract provide a reasonable attachment for the graphite particles and do not require to be coated per se to result in good contrast. Soft tissue biomechanics critically depends on the tested samples’ hydration state, which forms the rationale to adjust soft tissues to the condition in situ using an osmotic stress protocol prior to the biomechanical test^2,13^. Consequently, soft tissue surfaces generally provide reasonable dampness for the attachment of graphite particles as confirmed in a broad variety of tissues before reducing the application of coating sprays predominantly to the creation of areas differing in contrast.

## Limitations

The sample size of the study was limited due to the restricted amount of human tissues that could be obtained for the given project. In this study, the iliotibial tract was used as a model for collagen-rich human soft tissues. However, collagen-rich soft tissues of other body sites might reveal different results. Even though not visible to the naked eye, coating thicknesses may have differed between the individual samples with potential influence on the load-deformation properties obtained in this study. An attempt was made to keep the time between removal of the tested sample from the osmotic fluid and the end of the tensile test to a minimum (less than three minutes according to the experience of the authors) to prevent drying, as changes in water content would have altered the elastic modulus^13^. Conducting the mechanical tests in a humidity chamber might have prevented the specimens from uncontrolled drying^33^. Poisson's ratio might have provided additional valuable data on the here stated hypotheses. However, Poisson's ratio could not be calculated here due to a low number of facets that were available in the transverse plane.

## Conclusion

The load-deformation properties of the human iliotibial tract as a model for collagen-rich human soft tissues were unaffected by surface coating and graphite speckling. Water- and solvent-based colour coatings may equally be applied to speckle the human iliotibial tract and potentially other collagen-rich soft tissues for DIC purposes without significant effect on their quasi-static load-deformation properties (Supplementary Information S1).

## Supplementary information

Supplementary Information
