# Supplementary material for: Surface coating and speckling of the human iliotibial tract does not affect its load-deformation properties
Source: Sci Rep. 2020 Nov 27;10:20747. doi: 10.1038/s41598-020-77299-1 (PMC7695729; doi:10.1038/s41598-020-77299-1)
Supplement: Supplementary file 1 — Supplementary Information [file 41598_2020_77299_MOESM1_ESM.pdf]

Surface coating and speckling of the human iliotibial tract does not affect its load-deformation properties

Johann Zwirner[1*], Benjamin Ondruschka[2,3], Mario Scholze[4,5] & Niels Hammer[5-7]

[1] Department of Anatomy, University of Otago, Dunedin, New Zealand

[2] Institute of Legal Medicine, University Medical Center Hamburg-Eppendorf, Hamburg, Germany

[3] Institute of Legal Medicine, University of Leipzig, Leipzig, Germany

[4] Institute of Materials Science and Engineering, Chemnitz University of Technology, Chemnitz, Germany

[5] Department of Macroscopic and Clinical Anatomy, Medical University of Graz, Graz, Austria

[6] Department of Orthopaedic and Trauma Surgery, University of Leipzig, Leipzig, Germany

[7] Fraunhofer IWU, Dresden, Germany

* Correspondence and requests for materials should be addressed to J.Z. (e-mail: medijo@gmx.de)

DANTEC DYNAMICS

ISTRA 4D

Software Manual

Q-400 System

Manual Version: 4.4.7 v3
Software Version: 4.4.7.507
Date: 17. Sep 18
© Dantec Dynamics GmbH

NOVA INSTRUMENTS
a Nova Instruments company

CONTENTS

1. Starting ISTRA 4D. 1

1.1 Program Start 1

1.2 The License 1

2. The Repository 3

2.1 Activation of a Repository 3

2.2 The Repository Explorer 4
2.2.1 Modification of Elements 5

3. Import of Images or Data 7

3.1 Import of Images of a Correlation Series 8

3.2 Import of Images for a Calibration Series. 10

3.3 Import of Images for Manual Projection Calibration 14

3.4 Integration of external data 18
3.4.1 Conditions for integration 18
3.4.2 Import of data 18
3.4.3 Definition of Time Offset 19

4. Activation of Hardware 21

4.1 Select Hardware 21
4.1.1 Selection of an existing Hardware Configuration 22
4.1.2 Import of a Hardware Configuration 22
4.1.3 Creation of a new Hardware Configuration 23
4.1.4 Edit a Hardware Configuration 24

5. Acquisition of Images 28

5.1 Live Image View 29
5.1.1 Status Bar 30
5.1.2 Image Display 31
5.1.3 Acquisition Settings 33
5.1.4 Acquisition of Images. 44

5.2 Recording Procedure 49
5.2.1 Basic Elements of a Procedure 50
5.2.2 Load and Save Procedures 55
5.2.3 Edit Recording Procedure 55

6. Calibration . . . 56
6.1 Calibration Targets . . . 56
6.1.1 List of Calibration Target . . . 57
6.1.2 User Defined Calibration Targets . . . 58
6.2 Calibration Live Image . . . 61
6.3 Perform a Calibration . . . 63
6.3.1 Prepare the Calibration . . . 63
6.3.2 Positioning of the Calibration Target . . . 64
6.3.3 Selection of Calibration Target . . . 65
6.3.4 Calibration Mode . . . 66
6.3.5 Calibration Parameters . . . 71
6.3.6 Calibration Process . . . 73
6.3.7 Calibration Result . . . 77
6.3.8 2D Calibration . . . 77

7. Evaluation . . . 78
7.1 Create New Evaluation . . . 78
7.2 Start a Universal Correlation Evaluation . . . 82
7.3 The Control panel . . . 83
7.4 Input Data Selection . . . 84
7.5 Correlation Settings . . . 89
7.6 Mask Definition . . . 96
7.7 Startpoint search . . . 99

8. Visualization . . . 104
8.1 Multi Image Control View . . . 104
8.1.1 Select images . . . 104
8.1.2 Change Zoom Factor . . . 105
8.1.3 Arrangement of images . . . 105
8.2 Visualization of Measured Data . . . 105
8.2.1 Display of additional information . . . 106
8.3 Visualization of Evaluated Data . . . 108
8.4 2D Image Overlay . . . 109
8.4.1 Graphics . . . 109
8.4.2 Smoothing . . . 115
8.4.3 Gauge . . . 116
8.4.4 Coordinates . . . 128
8.4.5 Stencil . . . 132
8.4.6 Modules . . . 133
8.5 3D Model Visualization . . . 134
8.5.1 Orthographic projections . . . 135

8.6 Additional Features for Multi Camera Systems 137
8.6.1 Cylindrical Objects 137
8.6.2 Bi-Plane Objects 139

9. Real-Time functions 143

9.1 Live Display Control 144

9.2 Live Image 144

9.3 Positioning Assistance 145

9.4 Real-Time Correlation 147
9.4.1 Start a Real-Time Correlation 148
9.4.2 Visualization 149

9.5 Pattern Quality Evaluator 152

10. Export Functions 154

10.1 Export of Images 154

10.2 Export of Evaluated Data 156

11. ISTRA 4D Options 160

11.1 Environment 160
11.1.1 Unit System 160
11.1.2 Key Shortcuts 161
11.1.3 UI 162
11.1.4 Cache 162

11.2 Properties and Descriptions 162
11.2.1 Properties 163
11.2.2 Description 164

11.3 Mask Editor 167

11.4 Acquisition 168

11.5 Visualization 168
11.5.1 Appearence 169
11.5.2 Vector Data Style 169
11.5.3 Plot Style 170
11.5.4 Gauge Style 170
11.5.5 Show/Hide 171

11.6 Extensions 172

12. Miscellaneous 173

12.1 Additional View Options 173

12.2 Help Menu 173

12.3 Samples of Recording Procedures 174

1. Starting ISTRA 4D

The “**ISTRA 4D**” software is designed for controlling the measurement system Q-400 and for evaluating the data. After reading the following chapters you will be able to conduct measurements with the Q-400 system.

NOTE: ***ISTRA 4D*** *version 4.4.7 supports Windows 7 (64-bit) and Windows 10. Older Windows version are not supported.*

NOTE: *The installation of the software is described in the Installation and Update notes, which you can find on the software installation media.*

1.1 Program Start

After successful installation of the program the file “**ISTRA 4D**.exe” is located under the installation directory in the folder \bin. To start the software use the shortcut on the desktop or open the software using the Windows Start button and select **ISTRA 4D** in the Dantec Dynamics section.

Having started the program, the dialog “Select repository” comes up and a Data Repository needs to be selected. Open an existing repository or create a new one (2.1) and confirm by clicking the “OK” button. The program starts and provides the user interface for performing a new measurement, evaluation and visualization of existing data.

1.2 The License

A license consists always of a USB Dongle and the corresponding license file. Only this combination gives a valid license. It is possible to install the software and one license file on multiple computers, nevertheless only the version with the USB dongle is able to work.

If the software starts it looks for the last used license and checks for the corresponding dongle. If the dongle is not present an error message comes up explaining that the dongle for the active license is missing. The *License Explorer* is open (Figure 1). In the *Active License* section information about the actual license is given, like the *Owner*, the *Type*, the *Dealer* who creates the license and the *Date* when it was created. It also shows if the *Licence File* is *Valid* and the corresponding *Dongle* is attached to the system and matches to the license file. For special licences a *Password* might be *Required*. The password can be changed using the **CHANGE** button.

The Licenses section is showing all installed licenses on the computer and the active one (marked with an asterisk). The last used license is marked as the active license.

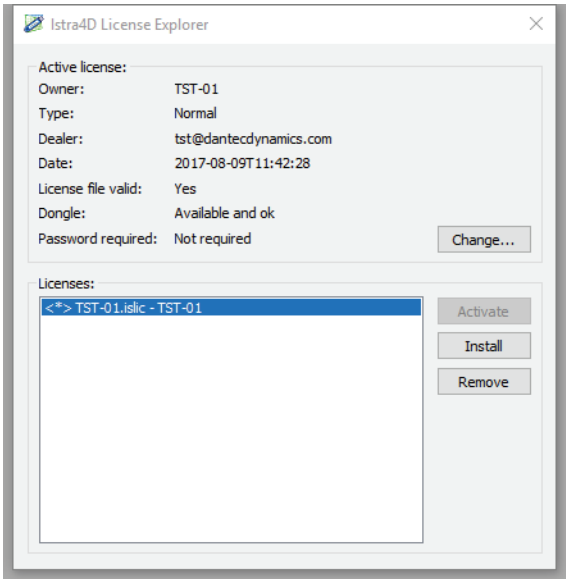

Figure 1 Dialog License Explorer

To install a new license on the computer use the **INSTALL** button and select the license file with the extension *islic* using the standard open file dialog. The new license appears in the list.

The selected license can be removed from the list using the **REMOVE** button. Activating a new license is done by selecting the license and pressing the **ACTIVATE** button. The software checks if the found dongle and selected license file matches and gives the feedback in the *Active License* section.

NOTE: *The license files are stored in the directory [Program files]\Istra 4D 4.4.7\config\licenses\ .*

*You need to run **ISTRA 4D** in administrator mode to modify licenses.*

2. The Repository

A repository contains references to several types of data and parameters which belong to a measurement. These are for example:

- Acquisition and evaluation parameters
- Calibration data
- Recorded images
- Evaluated data

A single repository may contain multiple measurements, evaluated data and calibration data. Selecting a repository will activate the data and parameters connected with this repository.

2.1 Activation of a Repository

ISTRA 4D provides a dialog “*Change Repository*” for activating an existing or a new repository. Only one repository can be loaded and edited at the same time. The dialog appears automatically after starting the program, however, you can change to another repository at any time during the operation of **ISTRA 4D**:

Select *Change Repository* under the *File* menu or using the Icon (Figure 2)

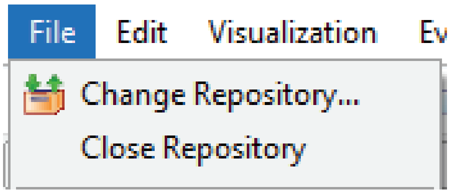

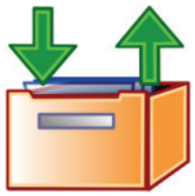

Figure 2 Change Repository

An existing Repository can be selected from the list or with the help of the Windows *Browse* button (Figure 3).

Alternatively a new repository can be created by entering the complete name of the repository directory to the input line. In the lower line the software gives the information whether this folder does exist. If not it will be created automatically.

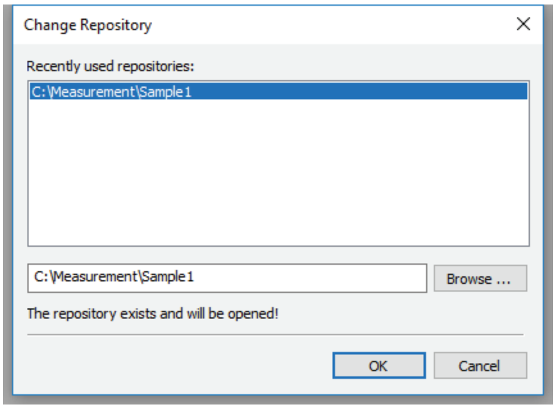

Figure 3 Dialog Change Repository

2.2 The Repository Explorer

The repository explorer manages measurement and evaluation data. It is represented as a folder in the Windows file system. The elements of the repository explorer are measured and evaluated data.

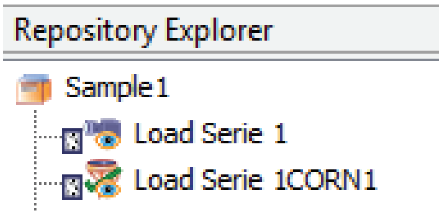

Figure 4 Repository Explorer

The type of the elements is indicated by different symbols:

Description	Icon
Measured data (series of images)	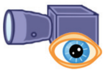
Evaluated data	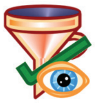

Table 1: Repository Icons

Description	Icon
No ISTRA4D data	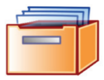
Not identified	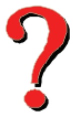

Table 1: Repository Icons

For the known entries in the *Repository Explorer* a *Tool Tip* window appears, if the mouse is moved over the element (Figure 5). The information depends on the type of data. E.g. for a series of measurement images the date of acquisition and type of measurement are displayed.

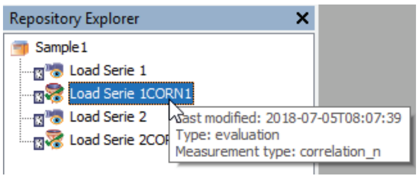

Figure 5 Repository Explorer Tool Tip

2.2.1 Modification of Elements

The elements in the Repository Explorer can be modified or special actions can be selected. Using the right mouse button a menu appears (Figure 6).

DEFAULT VISUALIZATION is equal to a *double-click* and starts the visualization of the selected data (see *Visualization*).

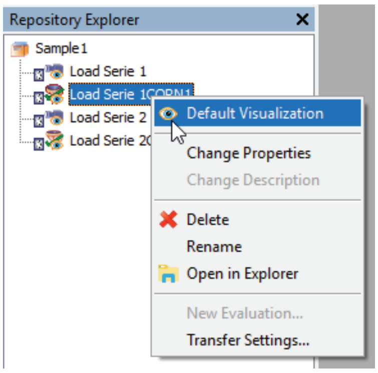

Figure 6 Repository Explorer Modify

Depending on the type of data the *Properties* or *Description* can be modified (see *Properties and Descriptions*).

The active element can be renamed by the **RENAME** function or removed using the **DELETE** function. **OPEN IN EXPLORER** will open the directory containing the data in the File Explorer.

NOTE: *To delete an element from the list the user has to confirm this action in an additional dialog.*

If the selected data is a series of images **NEW EVALUATION** starts an Evaluation (see *Create New Evaluation*).

TRANSFER SETTINGS enables coping of evaluation settings from an existing to the selected evaluation. The selection of the existing evaluation and the definition of the parameter to be used are selected within a dialog (Figure 7). As parameters the *Projection Parameter*s, *Correlation Parameters*, *Mask*, *Start Points* and the *Gauge Objects* are available (see *Start a Universal Correlation Evaluation*).

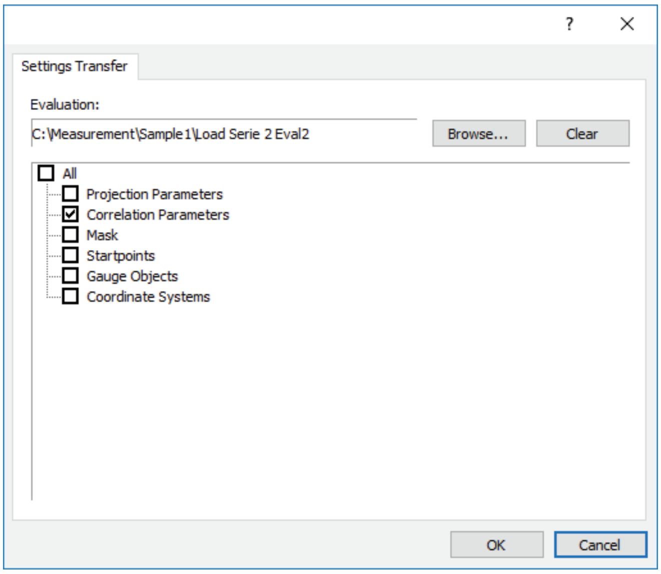

Figure 7 Transfer Evaluation Settings

3. Import of Images or Data[1]

The measurement of 3D contour, displacement and strain data requires a series of images, acquired using a stereoscopic setup. These images can be captured on-line using the **ISTRA 4D** software and hardware (*Activation of Hardware* and *Acquisition of Images*) or imported from files on the hard disk.

Tip: *This feature allows the* ***ISTRA 4D*** *software to process images form sources which are not integrated and controlled from the software package.*

The *File-Import Menu* gives the option to import images from a *Correlation Series* (images of the object at different loading conditions), images from a *Calibration Series* (images of the standard calibration target captured under different orientations) or images of an object which can be used for determination of a simple calibration (calculation of manual projection parameter) (Figure 8).

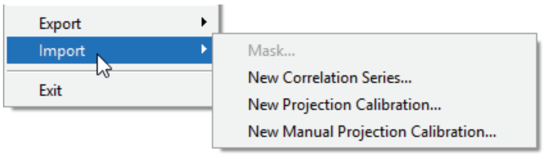

Figure 8 File-Import Series

NOTE: *A simple calibration is used if the projection parameter can't be determined by using the standard calibration procedure. Depending on the available information in the images only some of the projection parameters are calculated others are simplified.*

1. *The Import of series of Images is an optional feature and requires the corresponding licence option.*

3.1 Import of Images of a Correlation Series

A wizard for the required settings will be started. In the first page the list of images to be imported is specified (Figure 9).

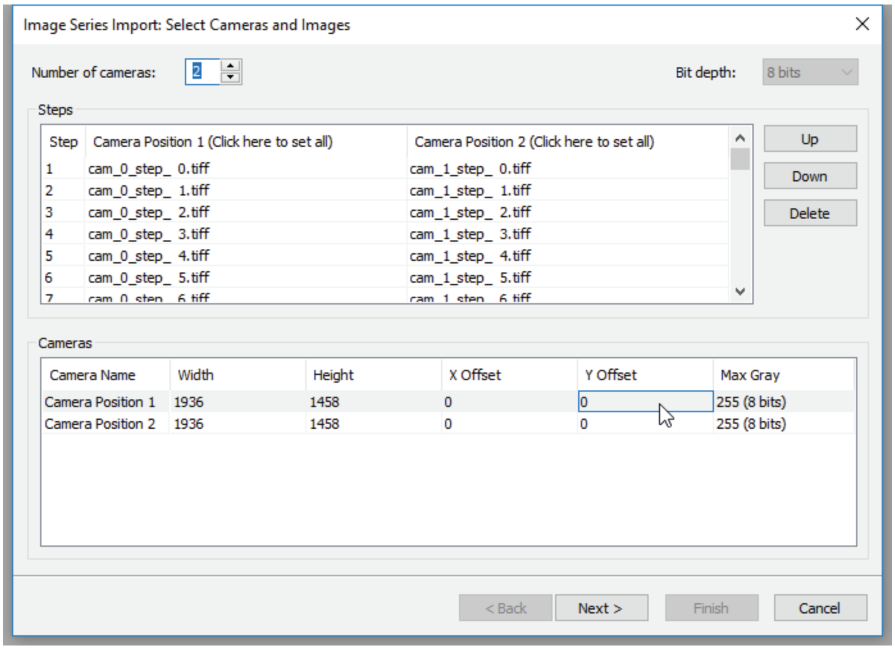

Figure 9 Dialog Import Series Select Cameras

The number of cameras used is selected in the field **NUMBER OF CAMERAS**. To insert multiple images for one camera click on the top of the column **CAMERA POSITION** this opens an *Open File Dialog* where multiple files can be selected. The selected files appear in the table. To add single images click in the corresponding field in the table.

NOTE: *The supported file types are BMP (8-bit) and TIF (8 and 16-bit) grey value images.*

The number of columns bust be a multiple of 4.

The order of the images can be changed within the list by activating the step and use the **UP** or **DOWN** button. The **DELETE** button removes the active step from the list.

The width and height of the images are displayed in the lower section. If an offset of the images is known this can be set in the field **X OFFSET** and **Y OFFSET**.

NOTE: *The dimension (width and height) of all imported images must be the same.*

In the next page the name of the entry in the repository is specified where the images are inserted (Figure 10).

In case the images are not *8bit* the bits to be used can be specified in the **BIT DEPTH** list.

If the frame rate of the recorded images is known the **ACQUISITION INTERVAL** can be used to save this information with the imported images. The frame rate can be selected from the list or time between the acquisition of two images can be typed in directly.

In addition the **DATE AND TIME** of the recording can be specified.

NOTE: *This information is saved in the HDF file structure.*

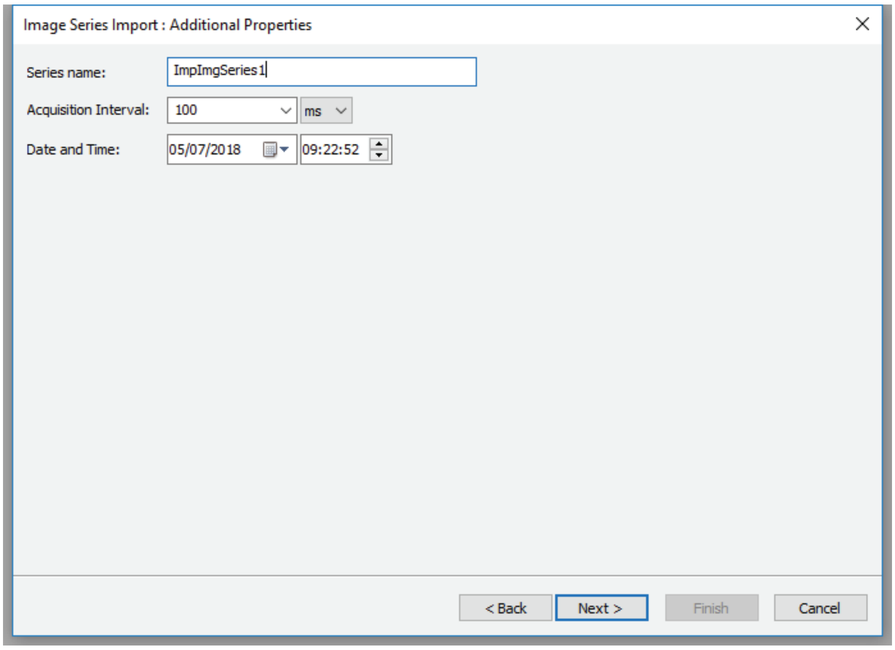

Figure 10 Dialog Import Series Select Name

On the last page the progress of importing of the images is displayed (Figure 11). After the successful import the wizard is closed by pressing the **FINISH** button.

Now a new entry with the imported series of images appears in the repository. This data can be used like any other series of acquired series of images

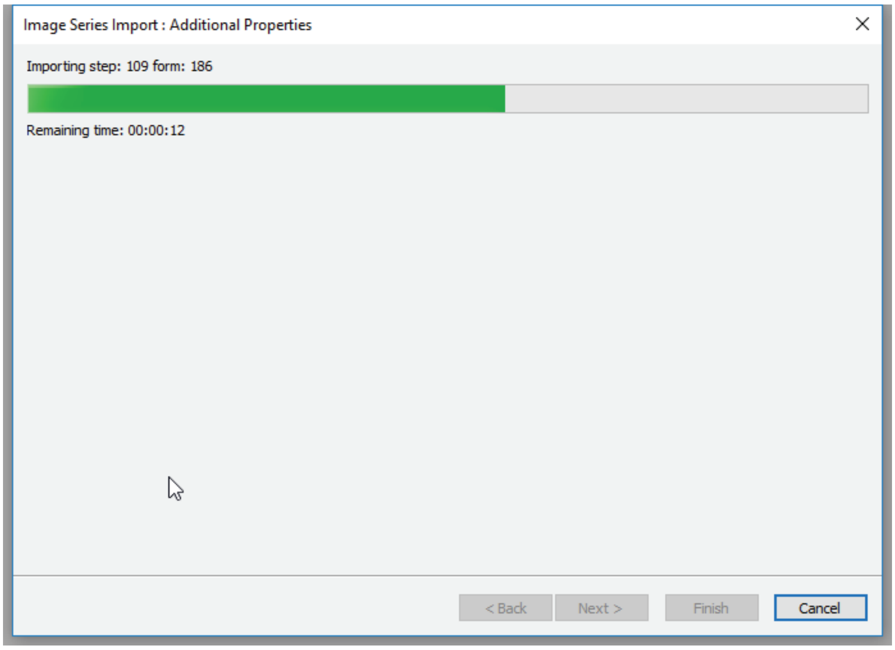

Figure 11 Dialog Import Series Progress

3.2 Import of Images for a Calibration Series

When using the supported cameras the calibration process for calculating the projection parameter is an automatic real time function (see *Perform a Calibration*). To be able to process images recorded with sources other than the supported cameras one can import images of a calibration target and use these images to calculate the projection parameter.

NOTE: *For the choice of the calibration target and the orientation during the calibration process the same requirements apply as with the automatic on-line calibration process (see Perform a Calibration).*

A wizard for the required settings will be started. In the first page the list of images to be imported is specified (Figure 9). Its use is the same as described in the previous Chapter 3.1.

The second page includes functions to perform the calculation of the projection parameter using the list of images selected before (Figure 12).

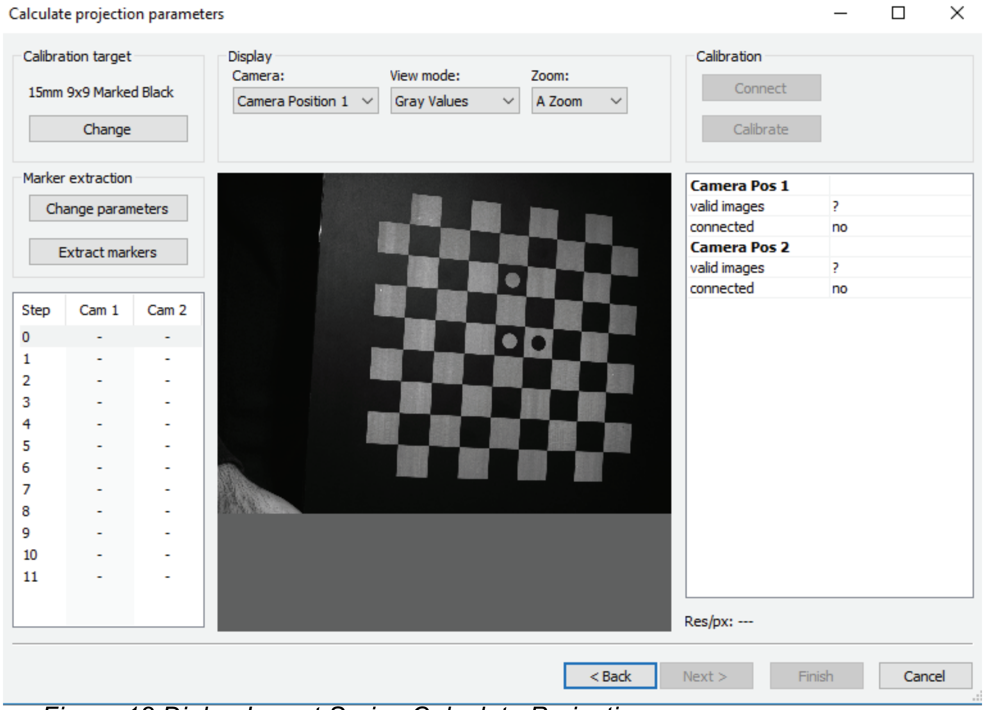

Figure 12 Dialog Import Series Calculate Projection

The software detects the calibration target. The display indication of the found markers is the same as in the automatic calibration mode (*Perform a Calibration*).

In the section *Calibration target* the actual used type of calibration target can be selected. The **CHANGE** button will open a dialog to select the type of target used. For more detail see also *Selection of Calibration Target*.

In the *Display* section the **CAMERA** option selects the camera which is displayed. Within the **VIEW MODE** option the mode of the image display can be changed. *Gray Values* shows the image as it is. In *BW Values* mode the segmentation of the image in black and white areas is displayed. The *Seg Line* mode shows the segmentation lines between the black and white areas. These dispay can be used

for checking the feature extraction (Figure 13). If required the parameters for the extraction of the markers can be adapted in order to improve the identification of the targets.

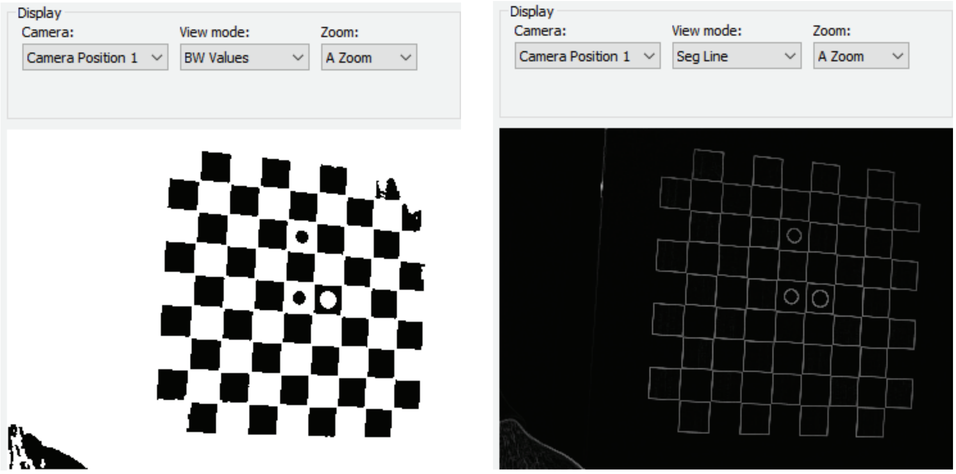

Figure 13 Display of imported images in BW (left) and Seg Line mode (right)

As a first step the markers in all images are detected and extracted. In the *Marker extraction* section the parameters for the detection of the markers can be adjusted in the section *Parameters*. Again the **CHANGE PARAMETER** button opens a dialog as described in *Calibration Parameters*. The **EXTRACT MARKERS** button starts the detection of the markers in all images (Figure 14). During the process the actual image is displayed. In the table on the left side for each step and camera the number of detected markers is shown.

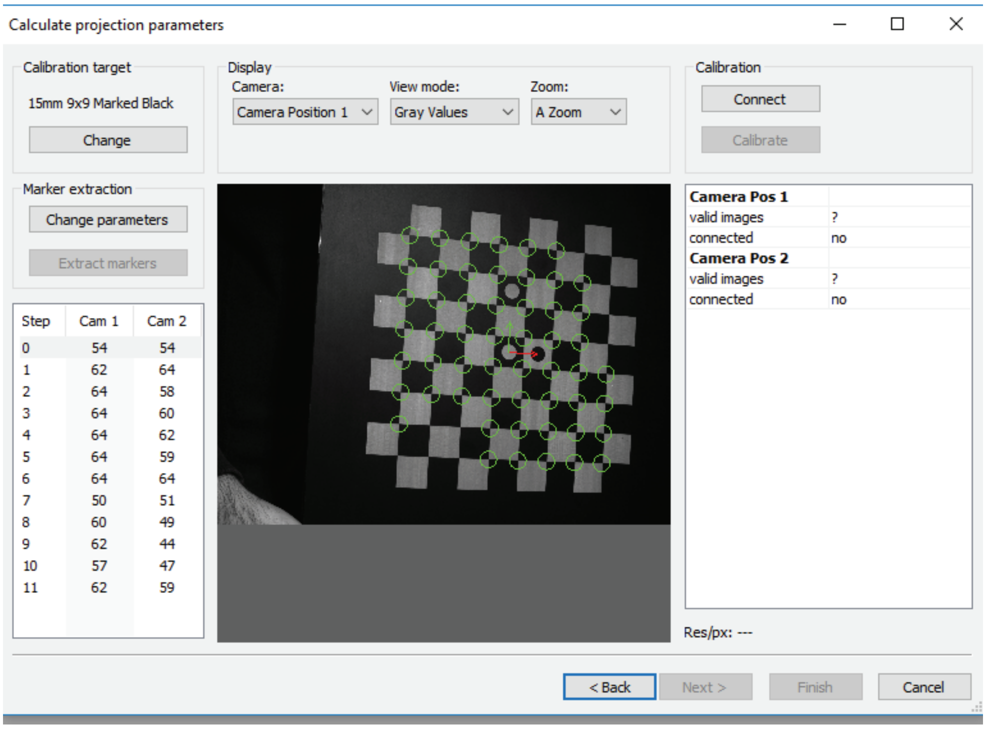

Figure 14 Import Series Projection Calculation Extract Markers

Now the calculation of the projection parameter can start. The next step is to check in which cameras the calibration target is visible for each step. The **CONNECT** button starts looking for each camera which steps the images can be used for (Figure 15).

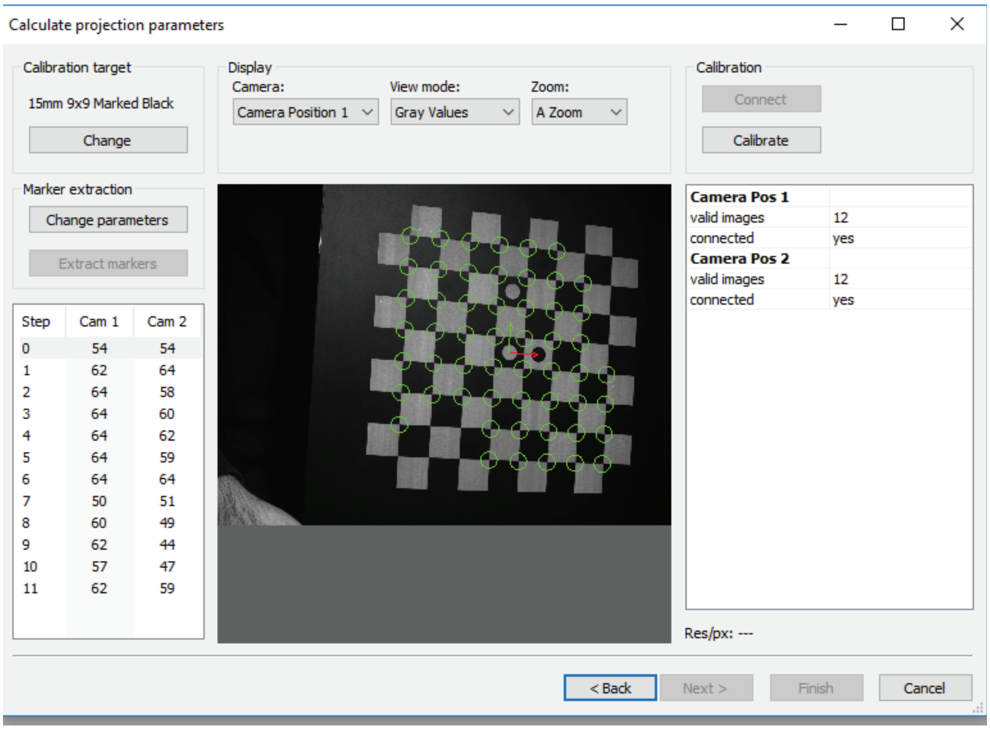

Figure 15 Import Series Projection Calculation Check Connectivity

NOTE: *The Check Connectivity step is required for the calculation of the projection parameter using multiple cameras.*

As the last step the calculation of the projection parameter for all cameras is performed with the **CALIBRATE** button. If the calculation of the projection parameter is finished the quality of the result is

indicated by the *Res* parameter (see also *Calibration Result*). The corrected residuum takes the uncertainty of the calibration target into account.

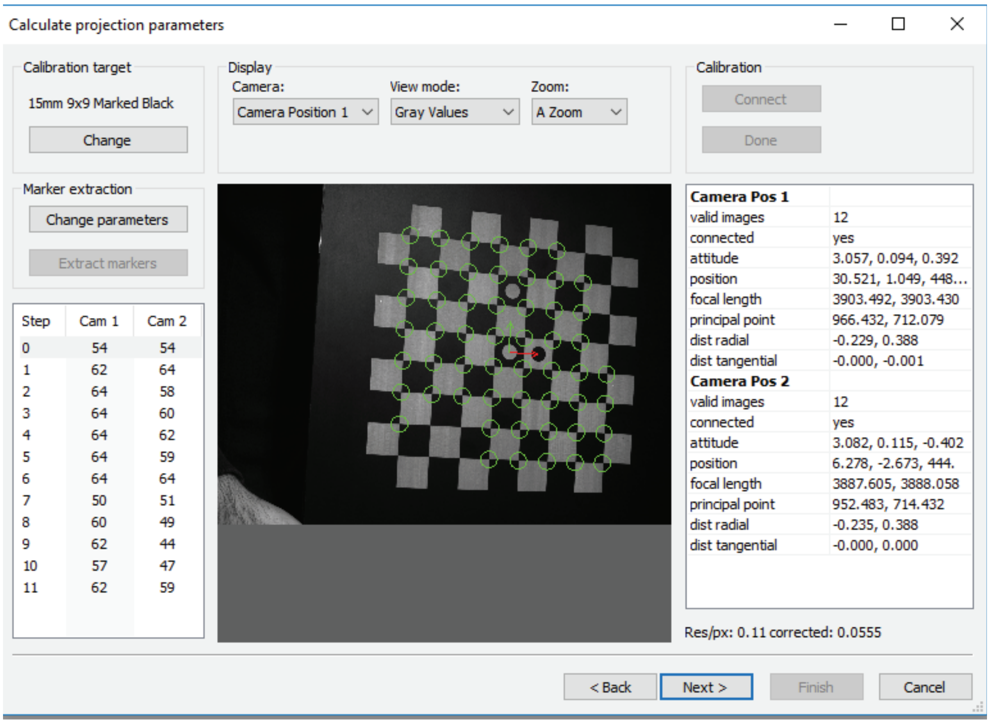

Figure 16 Import Series Projection Calculation Result

The **NEXT** button opens a page showing the calculated projection parameter and allows the selection of the file name for the data (see also *Calibration Result*).

3.3 Import of Images for Manual Projection Calibration

In case a proper calibration can't be performed, the manual projection calibration is designed to generate a projection parameter definition with reduced information (see also *Perform a Calibration*).

A wizard for the required settings will be started. In the first page the list of images to be imported is specified (Figure 9). Its use is the same as described in the previous Chapter 3.1.

The second page shows the first image with a line or markers. These elements are used the calculation of the projection parameters (Figure 17).

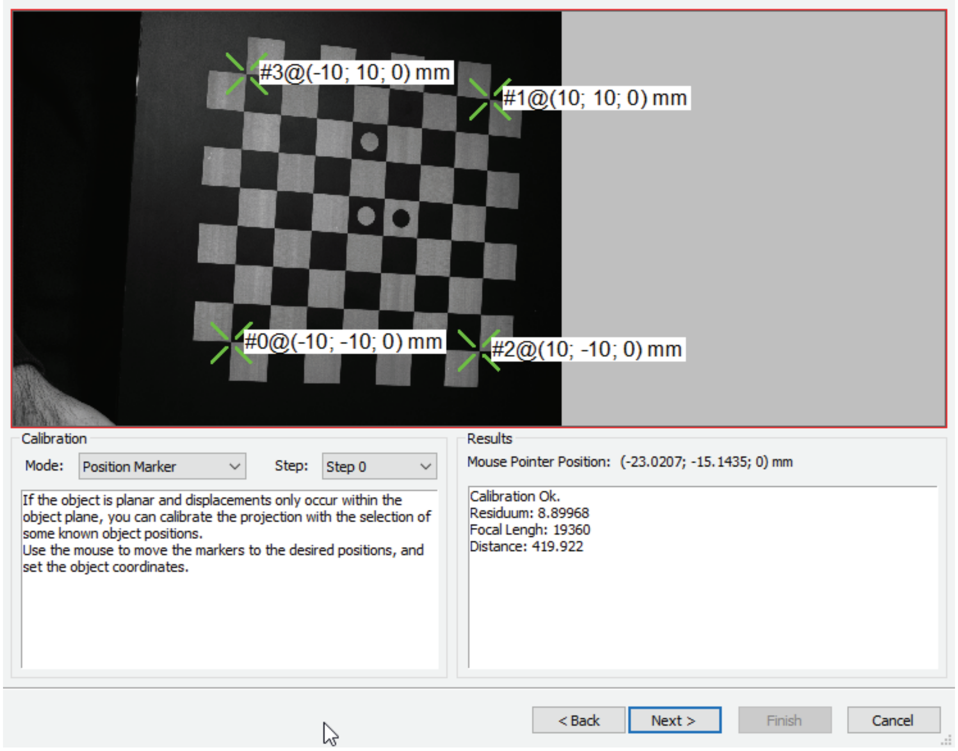

Figure 17 Dialog Define Marker Positions

In the **CALIBRATION** section the **MODE**, where the calculation is based on, is defined. The available modes depend on the number of images. If multiple steps are used the actual **STEP** is selected from the step list. An explanation for the mode is given in the text field below.

If possible the projection parameter, for the given marker position, is displayed in the **RESULTS** section. If the distance of two points is known for example, the focal length and the distance are assumptions and the scaling is given by the ratio of these values. At actual coordinate of the corresponding position on the object of the mouse is shown.

NOTE: *In general the value of the residuum is much higher than using a proper calibration. The reason is that here much less information is available and not all parameter, like distortions, are calculated. Therefore it's highly recommended using a proper calibration if possible.*

Depending on the number of images and the difference in distance of the marker to the camera the proper values can be calculated. Otherwise these values remain as dummy.

In the following the different modes for calculation of the projection parameter are explained.

Perpendicular Plane Mode

This mode is based on a plane object which is placed perpendicular to the direction of view. In this case the parameters are calculated by defining the distance between two points in the image (Figure 18).

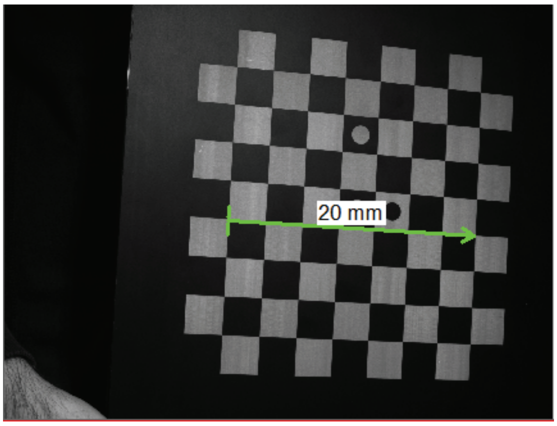

Figure 18 Definition of the position of the line

If the mouse is at one of the ends of the line a red cross is visible. By using the left mouse this point can be moved.

Tip: *The zoom function might be used to zoom into the image and fine positioning the marker.*

The distance between the ends of the line is defined by a click on the line. The color changes to red and a dialog pops up. Here the distance and the corresponding unit are selected (Figure 19).

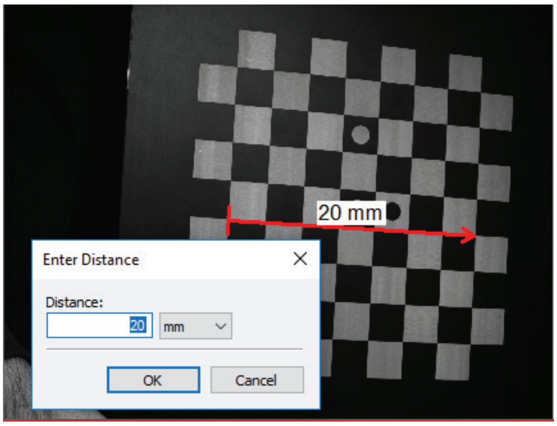

Figure 19 Definition of distance between two points

The start point of the line defines the origin and the end point the y-direction of the coordinate system.

Position Marker

This mode is based on the knowledge of the coordinates of multiple marker points on the object (Figure 20).

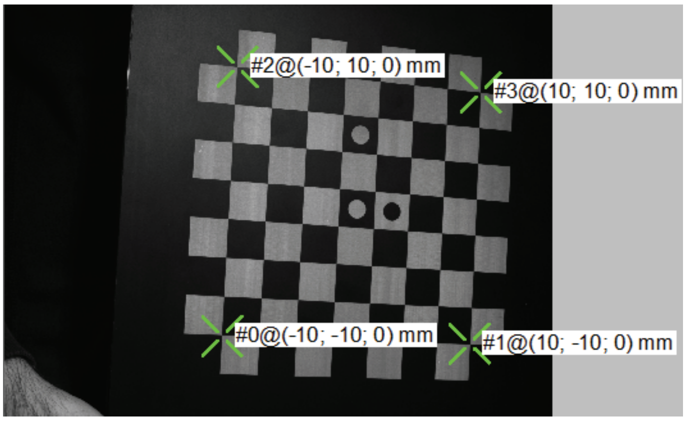

Figure 20 Position of marker in the image

If the mouse is at the marker, it changes red color and a right mouse opens a dialog, where this marker can be *deleted* or *setting coordinates* of this marker (Figure 21).

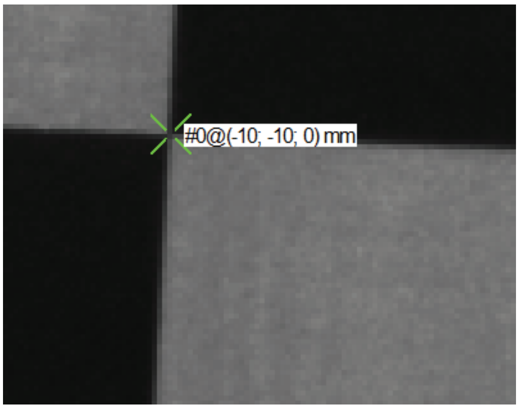

Figure 21 Position of marker in the image (zoomed)

The coordinates of the position of the marker on the object and the unit can be defined by using the corresponding dialog (Figure 22).

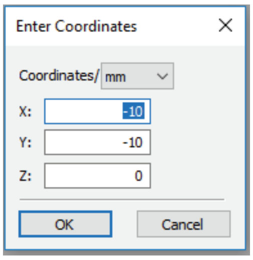

Figure 22 Dialog Entering Marker Coordinates

A right mouse click in the image opens a menu which allows adding another marker (*Append Marker*) or loading the position of markers from a previous manual projection parameter calculation (*Load Marker Positions*).

NOTE: *Only by using one camera and one image, the Perpendicular Plane Mode and the Marker Mode are available. All other configurations require the Marker Mode.*

3.4 Integration of external data

The integration of external data is a feature which allows the import of a time series of data channels into an existing evaluated data set. This can be used adding to a set of evaluated data external analog signals like force, path, temperature or similar.

3.4.1 Conditions for integration

The information of the external signal is expected to be in an ASCII file format.

NOTE: *At this time TRA files are supported. These file are generated typically from Zwick tensile testing machines. Other formats can be integrated on request.*

In order to add the external data to an existing evaluated data series each measurement step needs to have a time stamp. If the images are recorded using Istra4D the data have this time information automatically. If the images are imported via the Import wizard (Figure 10) it is essential that a time stamp has been generated.

NOTE: *In the case that the images are imported with an old version without time stamp the import and evaluation of the images has to be repeated.*

3.4.2 Import of data

The import is initiated by a right mouse click on the *Gauge Entries* (8.4.3) element in the gauge tree (Figure 23). The file with the data to be integrated is selected.

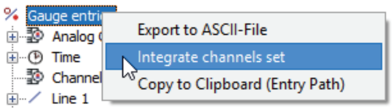

Figure 23 Menu Integrate External Channels Set

As first the file is analyzed and the columns with the time information is selected from the list (Figure 24).

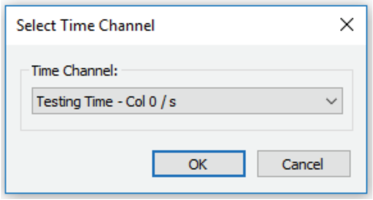

Figure 24 Dialog Select Time Channel

Following the other columns of the file are imported. They show up in the Gauge tree under the *Channels Sets* element (Figure 25).

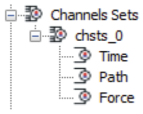

Figure 25 Integrated Channels Sets

3.4.3 Definition of Time Offset

In order to match the imported data with the evaluated data the time offset between these data sets has to be defined. In general imported data starts at a time of 0 second. A *Base Time Offset* is calculated from the time stamp of the evaluated data. If the time offset is known the user can add the exact or approximate value as Additional Offset from the context menu using a right mouse click on the Gauge Tree element (Figure 26).

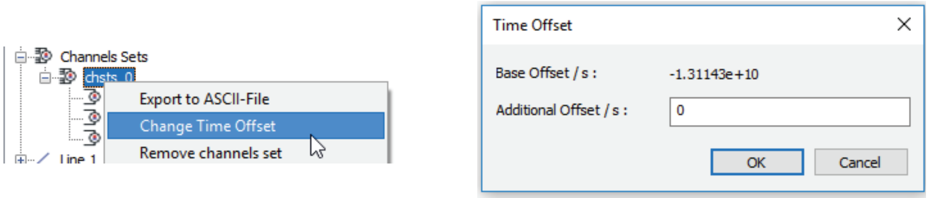

Figure 26 Menu Set Time Offset

Alternatively the determination of the time offset of the evaluated and imported can be performed by a visual match. For this matching the user shall select a significant measurement result, like displacement or strain of a point, where a match with the imported data is possible.

At first the imported data of the channel set are displayed as temporal plot. Using a right mouse click the data are displayed over time. Now the element of the evaluated data is selected and added to the temporal plot as second line(Figure 27).

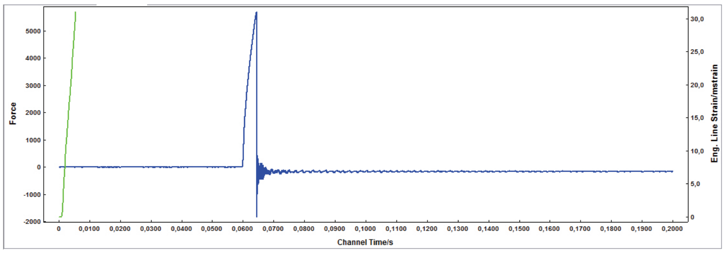

Figure 27 Temporal Plot Integrated External Channel (blue) and evaluated data (green).

NOTE: *In case you see you displayed the gauge element first and add the imported data, you will see the time range of the measurement only. In this case you can toggle the displayed time range by right mouse click on the graph between Full Time Range and Steps Time Range.*

In general the time range between the imported and evaluated data is different. Using the zoom function of the temporal plot (Figure 149) the remaining time offset can be determined and entered in the Time Offset dialog (Figure 26) so both curves of data match time vise (Figure 28).

Tip: *The user can use an iterative process of setting Offset Time and using zoom function in order to optimize the time offset.*

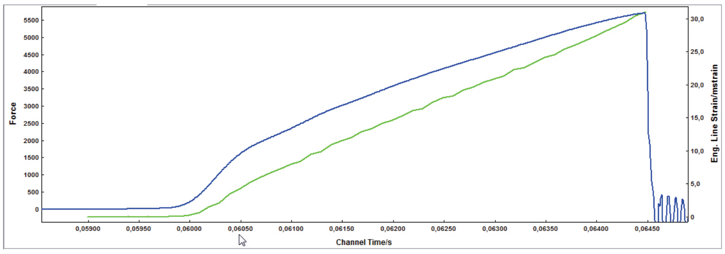

Figure 28 Temporal Plot Integrated External Channel (blue) and evaluated data (green) with optimized Time Offset.

4. Activation of Hardware

The hardware has to be activated explicitly in order to enter the image acquisition mode. Select *Hardware Activate* the Icon or the function from the *Acquisition Menu* (Figure 29).

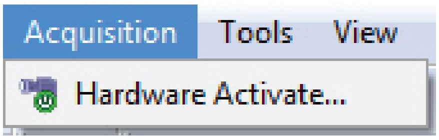

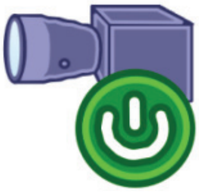

Figure 29 Hardware Activate

4.1 Select Hardware

A dialog with a list of available hardware configurations will be opened (Figure 30).

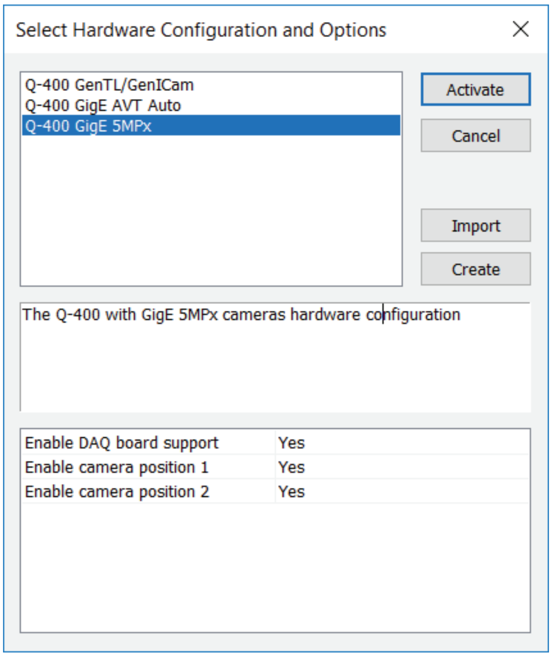

Figure 30 Dialog Select Hardware

Using this dialog an existing hardware configuration is selected or new configurations can be created.

NOTE: *The* **IMPORT** *and* **CREATE** *button are only accessible if* ***ISTRA 4D*** *is running as administrator mode. Otherwise the user can select an existing hardware configuration only.*

4.1.1 Selection of an existing Hardware Configuration

Select a configuration from the list In the upper part of the dialog. The text field below offers a description for the selected configuration. Additional external analog channels can be activated in the lower panel by setting **ENABLE DAQ BOARD SUPPORT** to **YES**.

If there is no DAQ BOARD installed, set the **ENABLE DAQ BOARD SUPPORT** to **NO**.

NOTE: *For the use of FireWire (IEEE 1394), Phantom HighSpeed and most GenTL/GenICam Cameras the DAQ board must be activated.*

USB3 Cameras and some GigE Cameras can be operated in Master/Slave mode without DAQ board. In Master/Slave mode the recording of analog signals and the use of external trigger signals is not possible.

Confirm the chosen configuration by pressing the **ACTIVATE** button. The system will initialize the hardware. This can take up to a few minutes.

4.1.2 Import of a Hardware Configuration

Using the **IMPORT** button the user can select a configuration which was exported before. The extension of the hardware configuration files is *ishcb* (Figure 31).

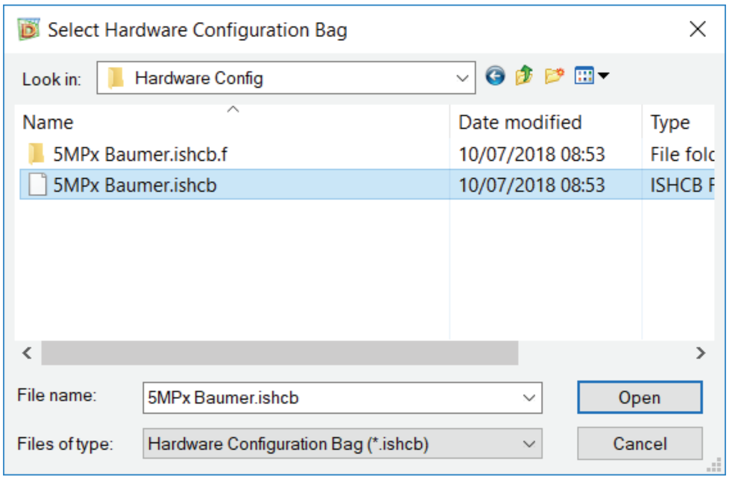

Figure 31 Dialog Import Hardware Configuration

Tip: *The Hardware Configurations of the system are exported before shipment and can be found on the installation medium delivered with the system.*

You may use these data, if you need to install the software on a new computer.

4.1.3 Creation of a new Hardware Configuration

A new Hardware Configuration is created by using the **CREATE** button. A list of available hardware templates is displayed. Select the type of hardware you want to create a configuration for and press the **OK** button (Figure 32).

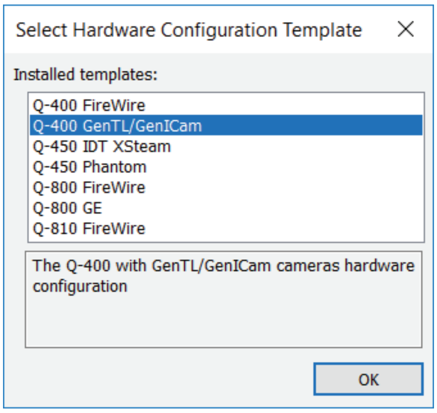

Figure 32 Dialog Select Hardware Configuration Template

NOTE: *The number and type of available templates depends on the modules selected at the installation procedure of the software*

Within the next dialog the user can change the display name and description of the configuration (Figure 33). This information will be displayed in the *Select Hardware* dialog (Figure 30).

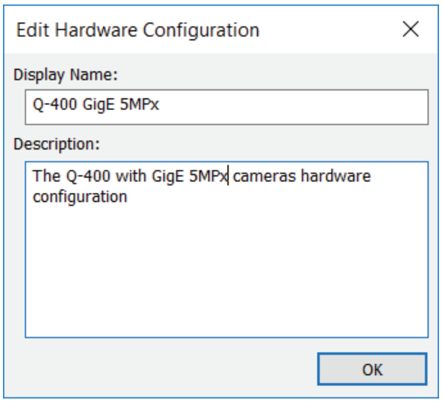

Figure 33 Dialog Edit Hardware Configuration

4.1.4 Edit a Hardware Configuration

Next step is the adjustment of the configuration files to the hardware to be used. A right mouse click on the Hardware Configuration in the Select Hardware dialog (Figure 34) opens a menu.

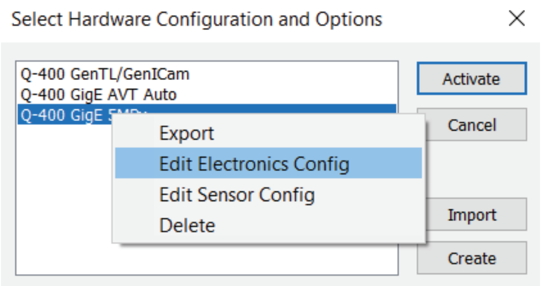

Figure 34 Menu Edit Configuration Files

Within this menu the Hardware Configuration can be exported (*Export*) as *ishcb* file, deleted (*Delete*) from the list or the configuration files can be edited. The type of Configuration files are for the electronics (*Edit Electronics Config*) and for the camera or sensor (*Edit Sensor Config*). The content of these files depend on the type of Hardware.

FireWire System

- Electronics Configuration file

  The Edit item opens the corresponding configuration file within notepad (Figure 35).

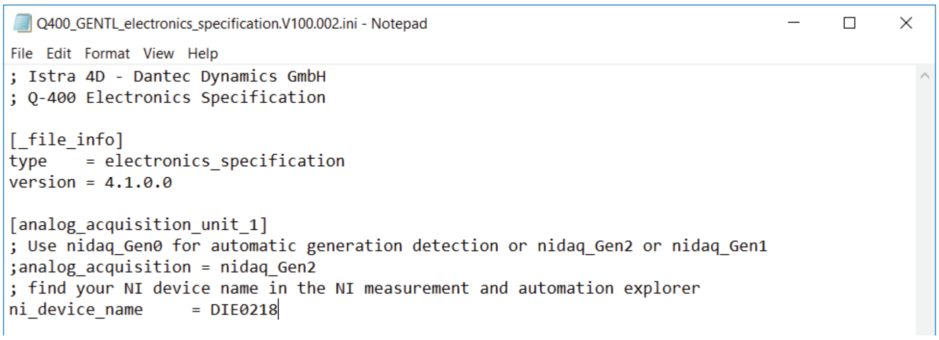

Figure 35 Edit Electronics Configuration File FireWire

In general only the last line including *ni_device_name* needs to be edited. Here the name of the control electronic should be assigned to the *ni_device_name*. The name is noted in the System

Configuration sheet, which is delivered with the manuals of the system. The entry is activated by removing the semicolon at the start of the line.

Tip: *In case the System Configuration sheet is not available, you may find the name in the in the section Devices and Interfaces of the NI Measurement & Automation software. If the electronics is connected via USB you find the name of the active device as the green icon and e. g. NI USB-6211.*

The changes in the file needs to be saved manually (*File-Save*)

- Sensor Configuration file

  The Edit item opens the corresponding configuration file within notepad (Figure 36).

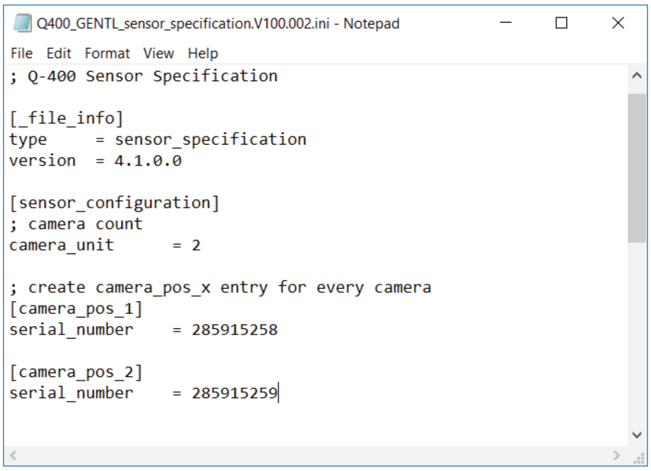

Figure 36 Edit SensorConfiguration File FireWire

Depending on the cameras in use the value of the *camera_unit* has to be adopted. Corresponding to this number the sections *camera_pos_n* needs to be changed. For each camera a serial number is required and must be assigned to *serial_number*. You find this information in the System Configuration Sheet.

Tip: *In case the System Configuration sheet is not available, you may leave the default values and try to activate the hardware. You will receive an error message. In this error message you find the serial numbers of the available cameras.*

The changes in the file needs to be saved manually (*File-Save*)

GenTL/GenICam System

Using the *GenTL/GenICam* intaerface different camera interfaces are supported. At this time *USB3*, *GigE* and *CoaXPress* interfaces are implemented.

- Electronics Configuration file

  The Edit item opens the corresponding configuration file within notepad (Figure 35).

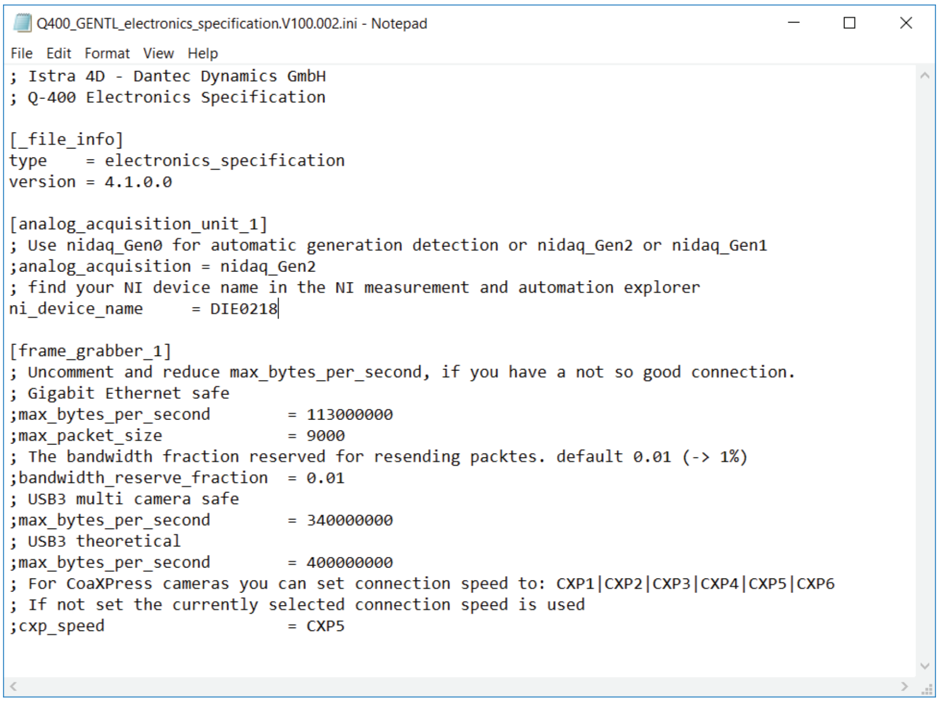
Q400_GENTL_electronics_specification.V100.002.ini - Notepad

File Edit Format View Help

```
; Istra 4D - Dantec Dynamics GmbH
; Q-400 Electronics Specification

[_file_info]
type    = electronics_specification
version = 4.1.0.0

[analog_acquisition_unit_1]
; Use nidaq_Gen0 for automatic generation detection or nidaq_Gen2 or nidaq_Gen1
;analog_acquisition = nidaq_Gen2
; find your NI device name in the NI measurement and automation explorer
ni_device_name     = DIE0218

[frame_grabber_1]
; Uncomment and reduce max_bytes_per_second, if you have a not so good connection.
; Gigabit Ethernet safe
;max_bytes_per_second        = 113000000
;max_packet_size             = 9000
; The bandwidth fraction reserved for resending packtes. default 0.01 (-> 1%)
;bandwidth_reserve_fraction  = 0.01
; USB3 multi camera safe
;max_bytes_per_second        = 340000000
; USB3 theoretical
;max_bytes_per_second        = 400000000
; For CoaXPress cameras you can set connection speed to: CXP1|CXP2|CXP3|CXP4|CXP5|CXP6
; If not set the currently selected connection speed is used
;cxp_speed                   = CXP5
```

Figure 37 Edit Electronics Configuration File GigE

In general only the *ni_device_name* entry in the *analog_acquisition_unit* section needs to be edited. Here the name of the control electronic should be assigned to the *ni_device_name*. The name is noted in the System Configuration sheet, which is delivered with the manuals of the system. The entry is activated by removing the semicolon at the start of the line.

Tip: *In case the System Configuration sheet is not available, you may find the name in the in the section Devices and Interfaces of the NI Measurement & Automation software. If the electronics is connected via USB you find the name of the active device as the green icon and e. g. NI USB-6211.*

In the frame_grabber section parameter controlling the data transfer of the GigE interface can be adjusted.

Tip: *In general the cameras shall be connected via a GigE connection which is exclusively used by the cameras.*

In case of a poor GigE connection you may reduce the max_bytes_per_second parameter to a smaller number. The bandwidth_reserve_fraction can be increased in case other services may use this GigE connection as well.

With USB3 the max bandwidth can be reduced in case there are some problems with the USB connection.

For CoaXPress systems the CXP speed can be set to a fixed value. Othervise the system uses the maximum available speed.

The changes in the file needs to be saved manually (*File-Save*)

- Sensor Configuration file
  The Edit item opens the corresponding configuration file within notepad (Figure 36).

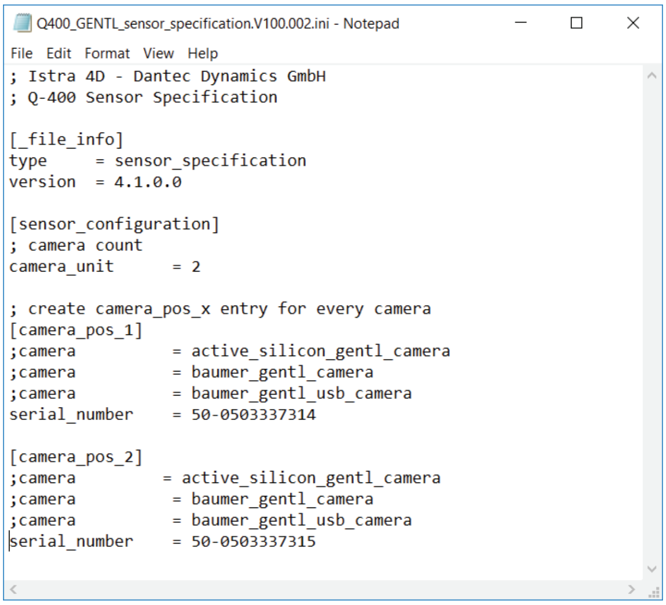

```
Q400_GENTL_sensor_specification.V100.002.ini - Notepad
File  Edit  Format  View  Help
; Istra 4D - Dantec Dynamics GmbH
; Q-400 Sensor Specification

[_file_info]
type      = sensor_specification
version   = 4.1.0.0

[sensor_configuration]
; camera count
camera_unit       = 2

; create camera_pos_x entry for every camera
[camera_pos_1]
;camera           = active_silicon_gentl_camera
;camera           = baumer_gentl_camera
;camera           = baumer_gentl_usb_camera
serial_number     = 50-0503337314

[camera_pos_2]
;camera          = active_silicon_gentl_camera
;camera           = baumer_gentl_camera
;camera           = baumer_gentl_usb_camera
serial_number     = 50-0503337315
```

Figure 38 Edit Sensor Configuration File GigE

Depending on the cameras in use the value of the *camera_unit* has to be adopted. Corresponding to this number the sections *camera_pos_n* needs to be changed. For each camera a serial number is required and must be assigned to *serial_number*. You find this information in the System Configuration Sheet.

Tip: *In case the System Configuration sheet is not available, you may leave the default values and try to activate the hardware. You will receive an error message. In this error message you find the serial numbers of the available cameras.*

Depending on the type of cameras used, the corresponding *camera* entry must be used. For GigE systems the defualt value can be used and the camera entry is deactivated. For Bauerm GigE cameras *baumer_gentl_camera should be used.* In case CoaXPress cameras are used, the camera entry with *active_silicon_gentl_camera* has to be used. For USB3 cameras the camera entry should be *baumer_gentl_usb_camera*.

The changes in the file needs to be saved manually (*File-Save*)

5. Acquisition of Images

Once the hardware is activated, the content of the *Acquisition Menu* changes (Figure 39) and an additional *Acquisition Toolbar* appears (Figure 41).

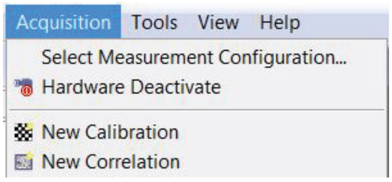

Figure 39 Acquisition Menu

The top-level *Measurement Configuration* allows the definition and selection of a configuration containing all parameters connected to the image acquisition. Like frame rate, shutter time, region of interest, analog input, ...

After activating the hardware, but before selecting a new calibration or correlation, the corresponding measurement configuration is selcetd.

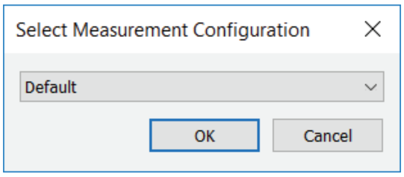

Figure 40 Select Measurement Configuration

From now on all measurement relevant settings are stored under this name. In order to recall the settings, the corresponding measurement configuration is selected.

NOTE: *In case of measuring objects under the same conditions, measurement configurations can be defined accordantly and can be loaded later.*

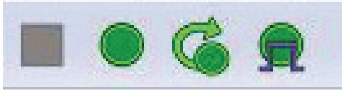

Figure 41 Acquisition Toolbar

To activate the display of the live image of the cameras click on the corresponding *Live Image* Icon.

Description	Icon
Deactivating the current hardware	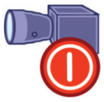
Start a new calibration setup	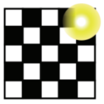
Open the Live Image view	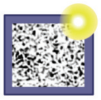

Table 2: Acquisition Mode Icons

5.1 Live Image View

Image acquisition and recording is controlled and managed within the Live Image mode. It can be activated with a click on the icon or the corresponding entry in the *Acquisition Menu* (Figure 39). The software open a window and displays the live images (Figure 42) in a Multi Control Image View (*Multi Image Control View*). As default all cameras are displayed.

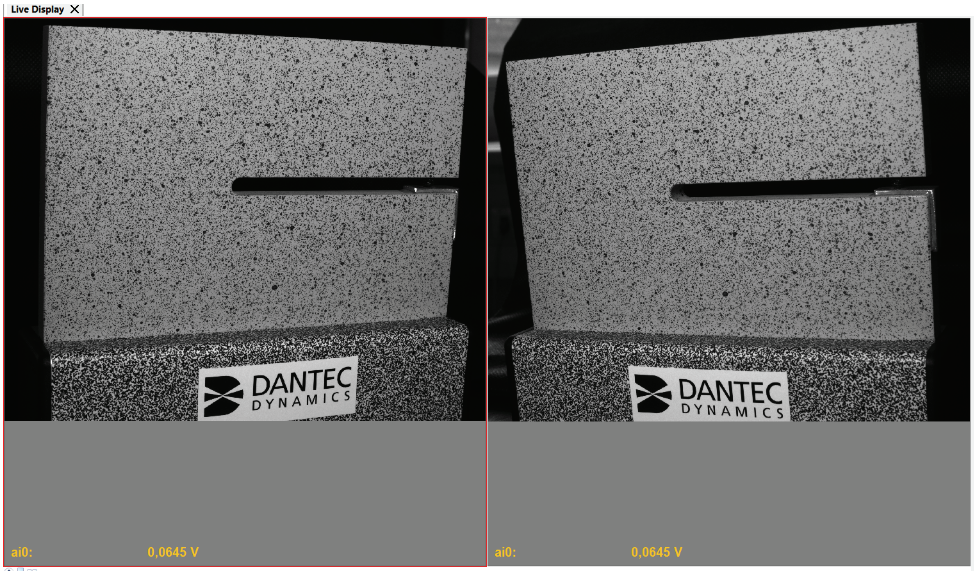

Figure 42 Live Image View

If multiple images or data are displayed as default only one is shown as full field. By using the View Menu or the View Toolbar a display of multiple data at the same time is possible (Figure 43). The arrangement can be switched between horizontal or vertical arrangement.

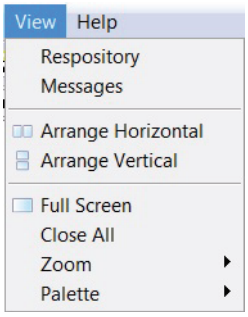

Figure 43 Arrange Windows

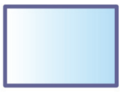

The *Full Screen* mode displays the program in a mode where only the program window is visible; all other elements including the Window Task Bar are hidden by the program. Using the *Full Screen* Icon the user can toggle between the full screen and the normal mode.

Close All will close all open windows. The *Zoom* and *Palette* selection defines magnification and the colors to be used for the display of the active window (see also *Image Display*)

5.1.1 Status Bar

The staus bar shows various information about the actual hardware in use and acquisition parameter. The name of the active hardware configuration is display (see *Select Hardware*). Beside this information the actual image size (see *Definition of Region of Interest (ROI)*) and the used frame rate (see *Acquisition Clock Setup*) are shown.

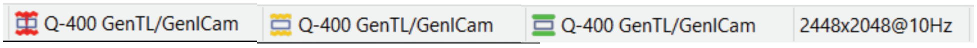

Figure 44 Status Bar

Once the hardware is activated, the software tries to monitor the temperature of the connceted camers. Depending on the temperature change rate different icons are displayed in the status bar reflecting the temperature stability.

NOTE: *If the camera does not support this feature, no icon is displayed*

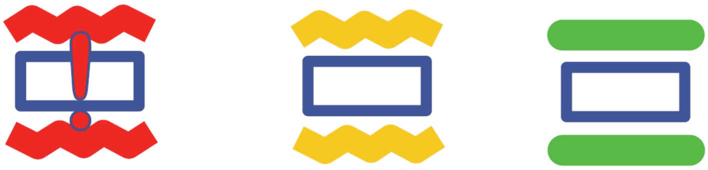

Figure 45 Camera Temperature Status

A red icon indicates, that the camera is not in a stable temperature stage. In this case the camera should not be used for image acquisition or calibration. If the icon turns into green color, the camera reached a stable state. Yellow indicates an in-between stage.

NOTE: *It is important, that the cameras have reached a stable temperature stage before acquire images for calibration or measurement. A temperature change may change the condition of the cameras in a way, that the projection parameter are not valid anymore.*

The camera temperature is stored with the calibration data and acquired images.

Additional toolbars appear for providing the full functionality of image acquisition:

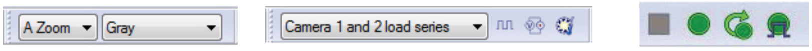

Figure 46 Acquisition Toolbar

The *Image Display* toolbar provides editable settings for a zoom factor and the color table for the active live image view (5.1.2).

Image acquisition can be configured using the *Acquisition Settings* toolbar (5.1.3).

Image recording is activated using the *Image Acquisition* toolbar (5.1.4).

5.1.2 Image Display

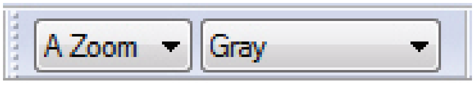

Figure 47 Image Display Toolbar

Description	
Selection of zoom for the display.<br><br>**A Zoom** is an automatic zoom function.	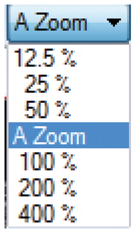
Select the color table for the display of the images.	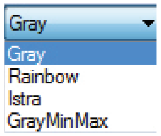

Table 3: Image Display Toolbar

Tip: *The zoom factor can also be adjusted by using the scroll wheel of the mouse (Change Zoom Factor)*

For the display of the images the user can choose one out of four different color tables:

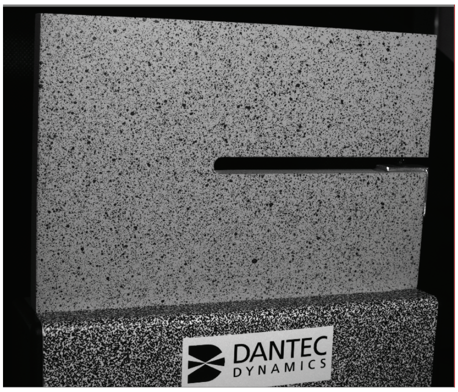

Gray: The images are displayed as gray color image

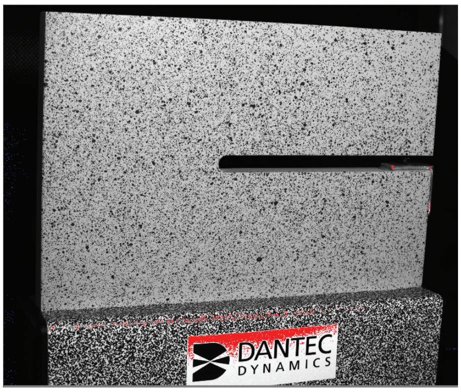

GrayMinMax: The images are displayed as gray color image, where the maximum value (white) is displayed in red and the minimum color (black) in blue color.

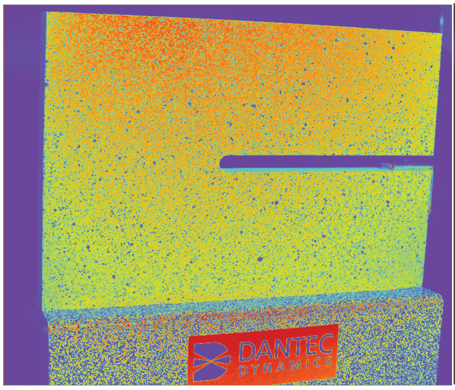

Rainbow: The image is displayed using a rainbow color table.

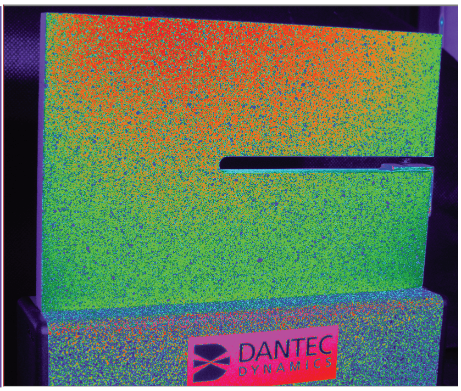

ISTRA: The image is displayed using a special color table which shows variations in the intensity easier.

Tip: *Using the GreyMinMax display is recommended for the adjustment of the focus. Open the aperture, adjust the intensity by the use of the illumination and/or the shutter speed in a way that parts of the object are displayed red. Then the position of the focus can be identified and positioned in the center of the object.*

5.1.3 Acquisition Settings

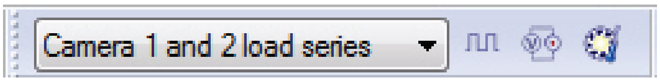

Figure 48 Acquisition Settings Toolbar

In the list box the user can select which camera configuration is used for the acquisition of the images for the measurement of the load series. For 3D measurements two cameras are required.

Description	Icon
Acquisition Clock Setup	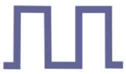
Analog Input Setup[a]	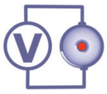
Brightness Setup	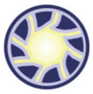

Table 4: Acquisition Settings Icons

a. The Analog Input Setup is only available if the DAQ board was activated (4.1.1)

In addition to the functions in the toolbar more functions are available in the *Acquisition* menu:

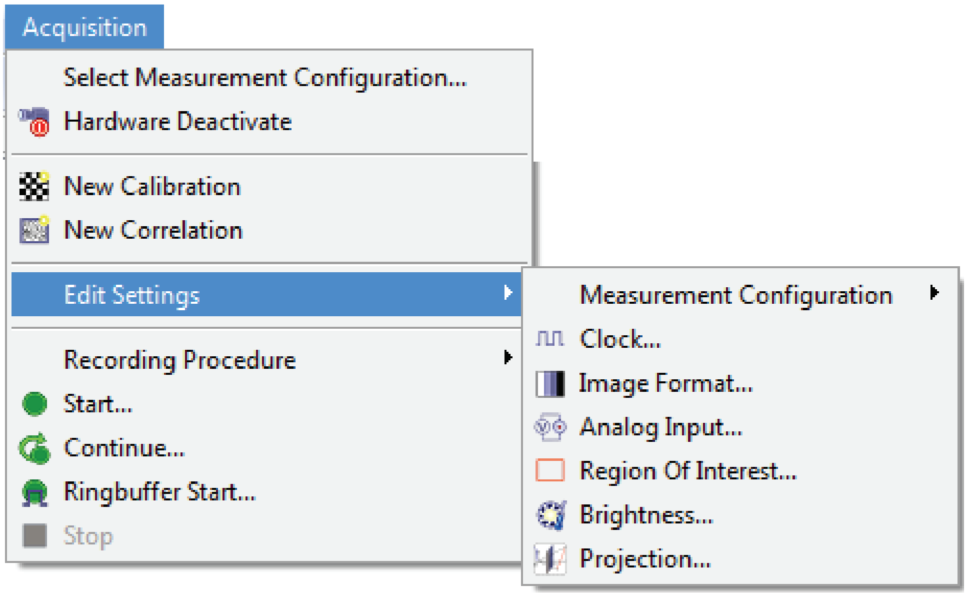

Figure 49 Acquisition Correlation Menu

With the *Measurement Configuration* in the *Edit Settings* section, the user can select a combination of cameras within the available cameras in the actual hardware configuration (Figure 50). The measurement configuration can also be selected using the drop down list from the settings toolbar (Figure 48).

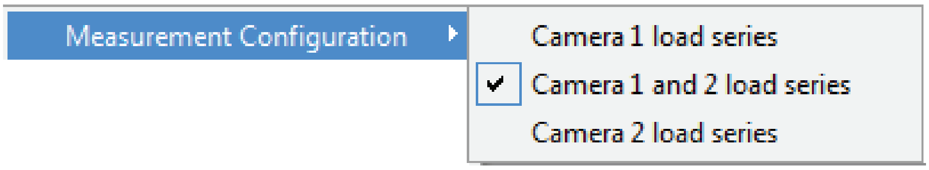

Figure 50 Acquisition Measurement Configuration

Description	Icon
Selection of image format	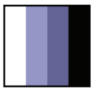
Definition of Region of Interest	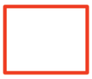
Setting for the default Projection parameter	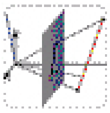

Table 5: Acquisition Settings Menu

Acquisition Clock Setup

The *Acquisition Clock Setup* defines the synchronization of the cameras and the timing of the image acquisition.

Depending on the hardware (4.1) different selections are possible. Within the **CLOCK SOURCE** list possible modes are listed.

In the *Master/Slave with internal clock* one of the cameras acts as a master. In this case the clock signal, which determine the acquisition of each image, is generated by one camera, the master.

This signal is given to the other cameras. In this case the image acquisition of the cameras is synchronized with each other but not with any external event.

NOTE: *The Master/Slave mode does not require the DAQ board.*

The recording of analog signals is not supported.

This mode is possible with USB3 and Baumer GigE cameras only.

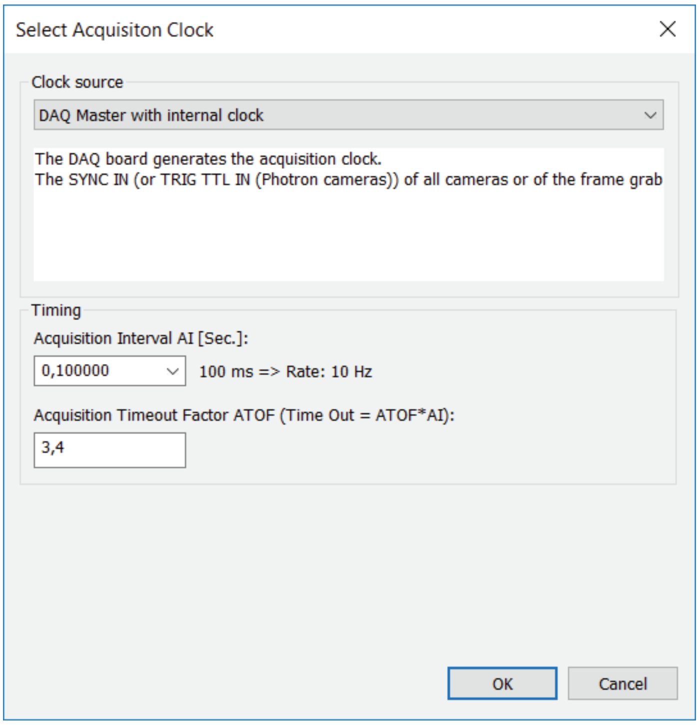

Figure 51 Select Acquisition Clock Dialog DAQ with internal clock

In the *DAQ Master mode with internal clock* the clock signal is generated from the DAQ board which acts as the master. The trigger in of each camera (BNC or orther) must be connected to the *Sync Out* of the timing box.

NOTE: *The DAQ board generates a periodic clock signal with a fixed frequency. This mode supports the acquisition of external analog signals.*

A DAQ Master mode is required to run the system with FW, GigE or CoaXPress cameras.

The **ACQUISITION INTERVAL** sets the time between the acquisition of two images and determines the frame rate. E.g an acquisition interval of 0.5 sec is a frame rate of 2 Hz.

The **ACQUISITION TIMEOUT FACTOR** defines the occurrence of a timeout message during the running image acquisition: If there is a lack of image transfers from the camera to the computer for a time interval longer than ATOF*Acquisition Interval AI, image acquisition stops with a timeout signal.

The *DAQ Master mode with external clock* uses the clock signal from an external source. This allows the determination of the acquisition of each image by an external signal. The external signal is connected to the *Sync In* connector of the timing box. The trigger in of each camera (BNC or others) must be connected to the *Sync Out* of the timing box. In this mode the shortest acquisition time must be specified (Figure 52).

NOTE: *The external clock signal is not limited to periodic signals. The time between two signals should not fall below the specified Minimal acquisition interval. The length of the signal should be about half of the Min Acquisition Interval.*

This mode supports the acquisition of external analog signals.

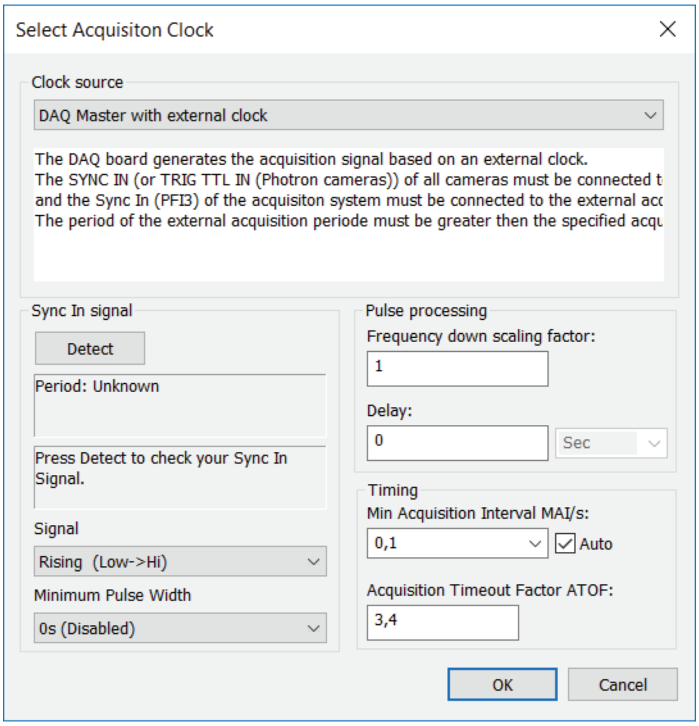

Figure 52 Select Acquisition Clock Dialog DAQ with external clock

If the *DAQ Master mode with external clock* is activated the frequency of the signal at the Sync In connector of the timing box can be calculated by using the **DETECT** button in the section *Sync In signal*. If successful the measured frequency is displayed in the field below the button. In the field below additional information is displayed. In the **SIGNAL EDGE** selection the user can define if a rising or falling edge of voltage defines the event. value of detected frequency. The **MINIMUM PULSE WIDTH** allows the selection of a minimum pulse width for the detection of the external clock signal. Within this time no additional trigger will be counted.

Tip: *The Minimum Pulse Width function helps detecting the correct frequency if the clock signal is not perfect but has some noise. It works as a debounder.*

Using the **FREQUENCY DOWN SCALING FACTOR** in the *Pulse processing* section the frequency of the Sync In signal can be down scaled. The **DELAY** value selects the delay between the occurrence of the Sync In signal and the generation of the Sync Out signal. The user can define if the delay is given in seconds or degrees.

NOTE: *The Frequency Scalar "n" can be any integer number n>3. Using this value the system will scale down the incoming frequency and not generate a Sync Out signal for each incoming Sync In signal but for each* n^{th}.

The Delay is related to the downscale frequency.

In the *Timing section* the **MIN ACQUISITION INTERVAL (MAI)** is specified. The MAI value is the shortest time between two valid signals. The reciprocal of the MAI is the maximum allowed frequency in Hz. If **AUTO** is activated the MAI value is automatically set by the detected frequency. The **ACQUISITION TIMEOUT FACTOR (ATF)** determines the maximum time the system is waiting for a Sync In signal before giving a timeout message.

Tip: *The value of the MAI should be smaller than the corresponding Analog Input Setup*

To use this function the DAQ board must be activated in the Activate Hardware dialog.

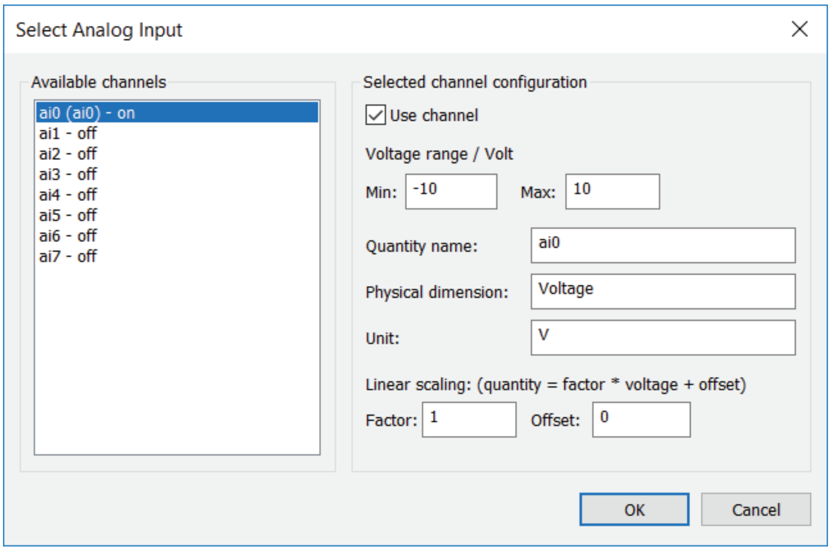

Figure 53 Select Analog Input Dialog

Open the Analog Input Setup dialog (Figure 53) to configure the analog channels in use. Each analog channel (A0 – A7) can be defined individually:

- Choose a channel from the list *Available channels*
- Activate selected channel by setting *Use channel*

- Edit the desired analog channel configurations. The **MIN MAX** values determine the sensitivity of the channel. The **QUANTITY NAME**, **PHYSICAL DIMENSION** and **UNIT** are used for display. With the **FACTOR** and **OFFSET** the recorded voltage can be scaled to another quantity.
- Confirm by clicking OK

The activated analog channels are displayed with the live images (Figure 54).

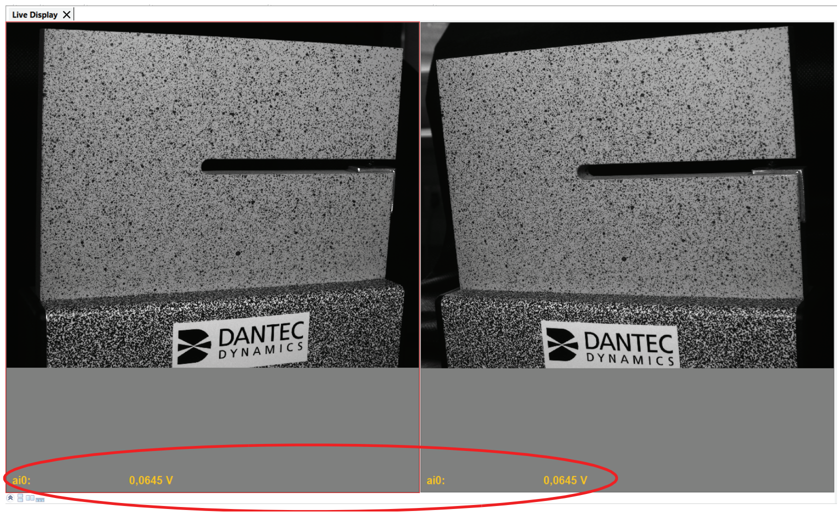

Figure 54 Live Image with Analog Channel

NOTE: *If the value of the recorded analog signal is floating at a constant input the ground level of the analog converter and the external ground are too different. In this case connect the external ground to the ground contact of the system.*[1]

1. Depending on the system configuration the ground contact is on the timing hub or the computer.

Brightness Setup

In order to adjust the intensities of the acquired images, the exposure or shutter time as well as the gain of the cameras can be set with the dialog *Brightness Setup*. (Figure 55)

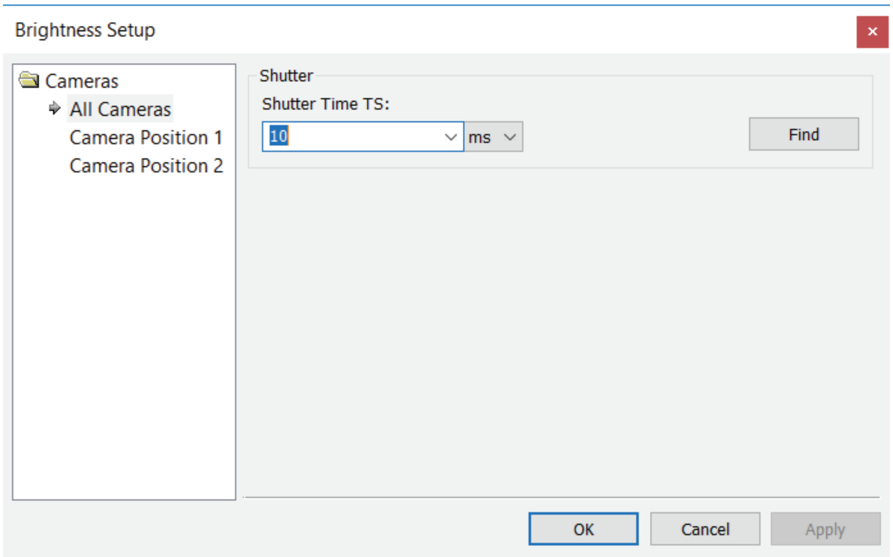

Figure 55 Brightness Setup Dialog[1]

In the control for **ALL CAMERAS** the user can define a common **SHUTTER TIME** which can be applied to all cameras. The **SHUTTER TIME** can be selected from a list or alternatively can be typed in. The maximum *Shutter Time* is determined by the used *Acquisition Interval*.

1. The layout of this dialog varies with the used cameras

The **FIND** button starts an automatic adjustment of the *Shutter Time* to reach a good intensity distribution in the images.

Tip: *The use of a common Shutter Time for all cameras is recommended for the measurement of dynamic events.*

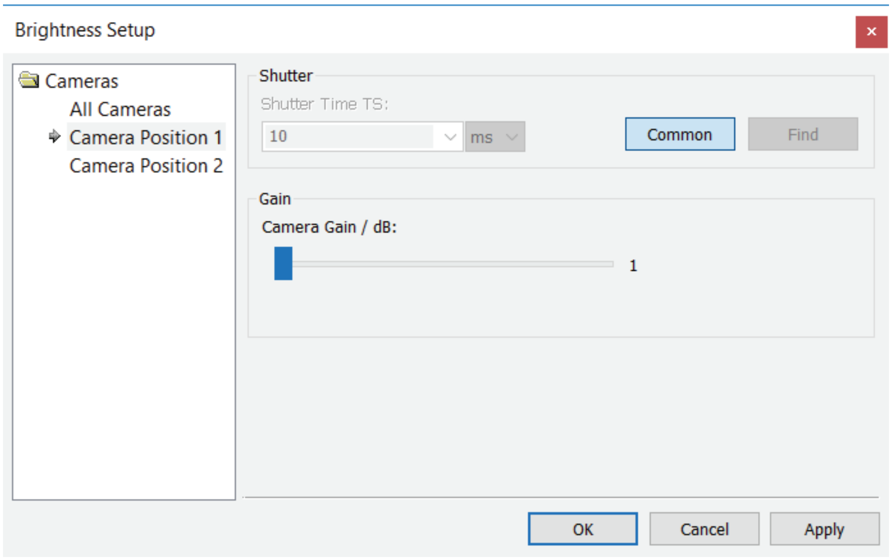

Figure 56 Brightness Setup Dialog[1]

In the control for each camera the **COMMON** button selects if the shutter global **SHUTTER TIME** is used for this camera or the Shutter Time is defined individually. The actual camera can be selected in the section **CAMERAS** in the left part of the dialog.

NOTE: *In order to use for all cameras the common shutter time the common function has to be activated for all cameras.*

If the function is supported by the actual camera the **GAIN OF THE CAMERA** can be changed.

NOTE: *It is strongly advised not using the gain, as this will increase the noise level of the cameras.*

1. The layout of this dialog varies with the used cameras

Selection of image format

Depending on the type of camera, images can be stored in different formats, e.g. a camera may acquire 10-bit images but only 8-bit images are used (Figure 57).

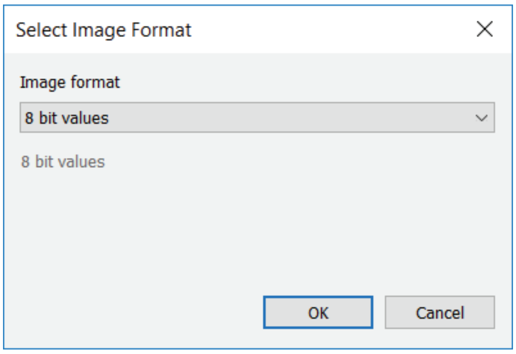

Figure 57 Select Image Format Dialog

The display of the images is always in 8-bit format, even if the images are saved as 16-bit.

Definition of Region of Interest (ROI)

Optionally the size of the field of view for image acquisition can be diminished from the whole camera field of view to a smaller window.

The region of interest can be defined interactively using the ROI setup dialog. In this case the system won't store the complete images but merely the windowed area. The size of the field of view must be the same for both cameras, the position in the image may be different.

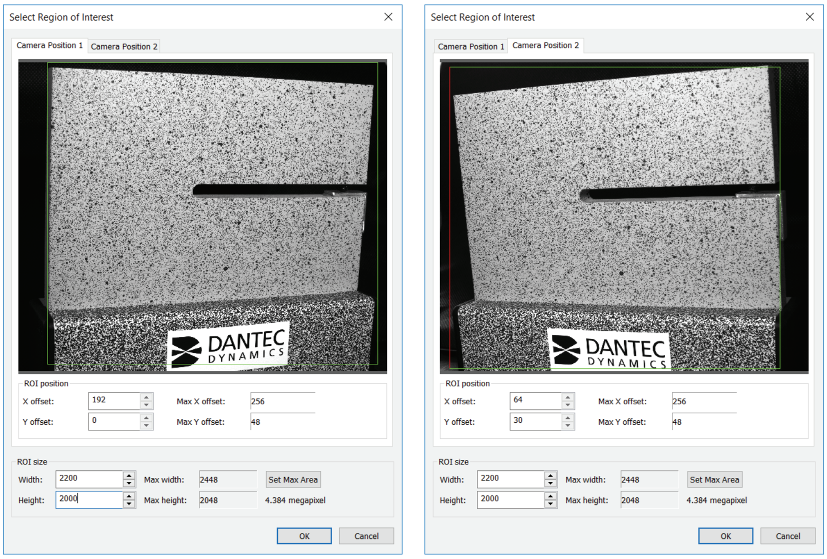

Figure 58 Definition of Region of Interest Dialog

Depending on the image acquisition frame rate the number of available pixels in vertical and horizontal direction may be limited to a number smaller than the camera CCD pixels. The number of pixel in horizontal (**WIDTH**) and vertical (**HEIGHT**) direction can be set in the section *ROI Size*. The maximum possible ROI size is displayed as **MAX WIDTH** and **MAX HEIGHT** (Figure 58) and can be selected by the **USE MAX** button.

In the upper part of the dialog the actual selection of the ROI is shown as a rectangle overlay. The position can be adjusted by setting an offset in horizontal (**X OFFSET**) and vertical (**Y OFFSET**)direction.

The size and the position can be adjusted by changing the values or by using the mouse. If the mouse is over the left side or the top line of the rectangle the color will change to red (Figure 58, left). Now this line can be dragged by a left mouse click. If the mouse cursor is inside the rectangle the rectangle will turn into red color and can be moved (Figure 58, right) by the mouse.

Since the size of the ROI needs to be same in all images a change of the size of the ROI in one image will change the rectangle in all other images too.

NOTE: *The number of displayed cameras depends on the number of active cameras.*

Depending on the camera type not all parameters may be available.

Projection Parameter

Within the software one set of projection parameters can be selected as default. A description of the actual parameter used is displayed in the upper part of the dialog. Using the **BROWSE** button another file can be selected (Figure 59).

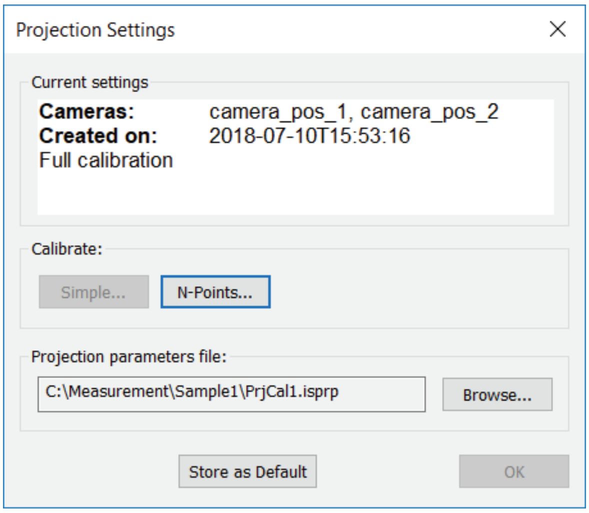

Figure 59 Select Projection Parameter Dialog

NOTE: *The N-Point method for the determination of the projection parameter is not implemented fully at this time.*

5.1.4 Acquisition of Images

The acquisition of images is started by clicking on the corresponding icon in the *Image Acquisition* toolbar (Table 6:). There are different configurations for the timing and triggering of the acquisition depending on the hardware.

Manual Acquisition

The first step is to set the type of acquisition and name for the set of images (Figure 60). At this time only *Load Series* can be acquired. The second tab allows the settings of the properties for the measurement series (see also *Properties and Descriptions*).

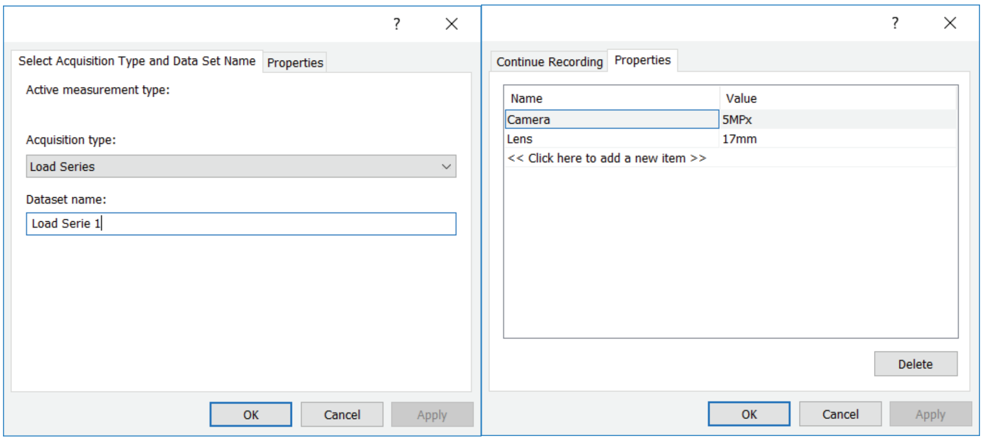

Figure 60 Select Acquisition Type Dialog

The **DATA SET NAME** is the name which appears in the repository explorer and where all the acquired images are stored.

If **CONTINUE RECORDING** is selected a dialog with the existing series appears. The series which is to be continued is selected.

NOTE: *The following settings must be identical to the existing series: Image size, ROI settings, Analog Input settings.*

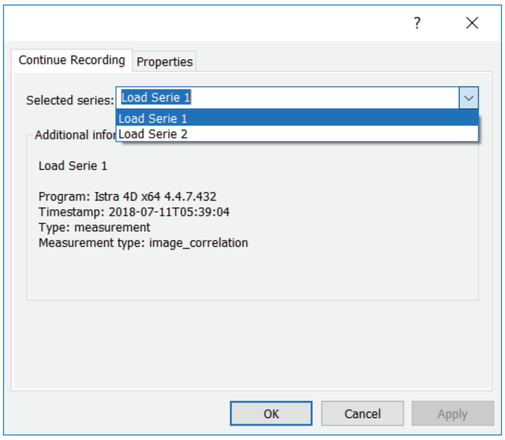

Figure 61 Continue Recording Dialog

The Image Acquisition toolbar is then expanded to icons controlling the acquisition (Figure 62).

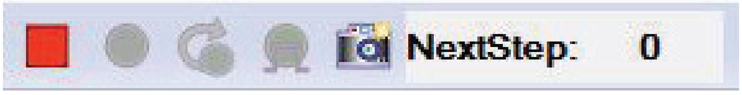

Figure 62 Image Acquisition toolbar

Clicking on the *Take Snapshot* icon will acquire images from the cameras and save them in the active repository. For a visual feedback the display of the live images is inverted when the image is acquired. The number on the right side indicates the number of the next step.

The acquisition is also started if a TTL signal is detected at the *Trigger-In* connection of the timing box.

Description	Icon
Start Recording	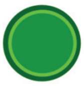
Continue Recording	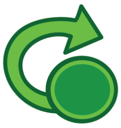
Stop Recording	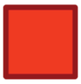
Take Snapshot of the Actual Acquisition	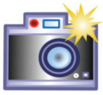
Start Triggered Recording	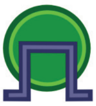
Set Manual Trigger	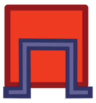

Table 6: Acquisition Icons

Pre- Post Trigger Acquisition

Start a new *Pre-Post Trigger* acquisition series by selecting the corresponding toolbar icon. In that mode the cameras continuously capture images in an internal ring buffer. After the occurrence of a single trigger signal a configurable number of frames before and

after the signal are read out. A corresponding dialog for setting the number of images before and after the trigger event pops up after activating the Pre-Post Trigger mode (Figure 63)

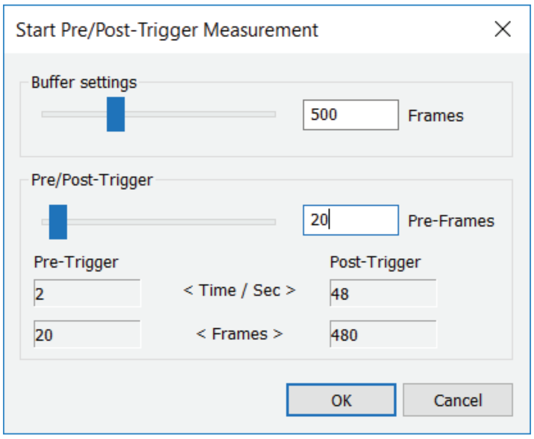

Figure 63 Dialog Pre-Post Trigger Settings

In the *Buffer Settings* section the size of the ring buffer is defined by the use of the slider. In the *Pre-Post Trigger* section the position of the trigger within the ring buffer is defined by setting the slider to the corresponding position. The position is displayed in the lower part as the time, and number of frames before and after the trigger event.

NOTE: *The maximum size of the ring buffer is limited by the hardware used. The time before and after the trigger event depends also on the frame rate selected in the Acquisition Clock Setup.*

Confirm your settings by pressing the **OK** button. Now the system enters the camera live image mode. Press the *Manual Trigger* icon to initiate the trigger event. The trigger can also be activated by an analog TTL signal at the *Trigger In* connector of the timing box.

NOTE: *The software allocates the required memory after leaving the dialog. In case that not enough memory was available an error message is shown and you need to restart the software.*

Depending on the version of the timing hub the software can check if a trigger was detected and deactivate the trigger icon.

The trigger icon is active if at least the number of pre-images is recorded.

The images, captured according to the Pre-Post Trigger configuration, are displayed. In order to scan through the captured images, use the *Video Display* Toolbar (Figure 64).

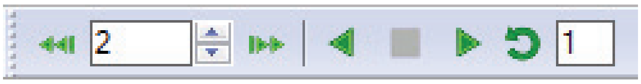

Figure 64 Video Display Toolbar

The left side elements control the display of a single step. Here the number of the step to be displayed can be entered manually or by using the up-/down arrows. The other elements control the display of the images as a movie.

Description	Icon
Display the first image	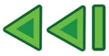
Display the last image	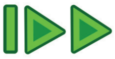
Select the number of image to be displayed	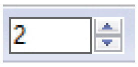
Starts to play the images as a movie forward	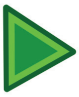
Starts to play the images as a movie backwards	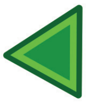
Plays the movie in a loop	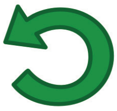
Set the number of images per second	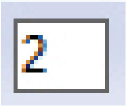

Table 7: Video Display Icons

At that state the images are still kept in the memory of the cameras or respectively the memory of the computer. The readout has to be initiated explicitly by the user. Click on the *Save* icon or the Save entry in the *File Menu* to open a dialog for the configuration of the images to be read out (Figure 65).

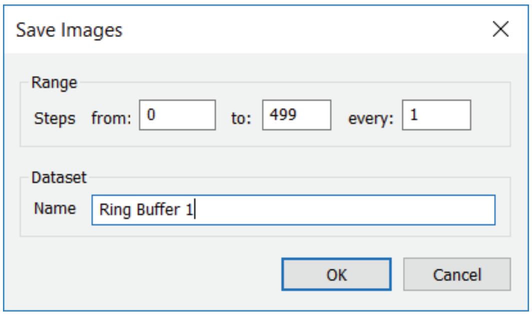

Figure 65 Dialog Save Images from the Buffer

In the *Range* section the images to be saved are defined. The first images is **FROM** up **TO** the last image. The increment is given as **EVERY**. After confirming the dialog settings with **OK**, a new measurement series, containing the captured data, will be created. A corresponding series name can be defined in the *Data Set* section of the dialog as **NAME**. The new series appears as an entry in the repository explorer.

NOTE: *Saving the images does not delete the images from the buffer. Until the acquisition mode is stopped new sequences of images from the buffer can be saved as different series in the repository explorer.*

5.2 Recording Procedure[1]

The *Recording Procedure* function is designed for automatic controlled acquisition of images.

An acquisition can be triggered by several different events like image number, time or changes of analog signals. The acquired images are saved directly on the hard disk. As the maximum storage rate limits the speed of this feature it is specified to be used for slower events (<1Hz).

Tip: *Depending on the hardware used and the size of the images it‘s possible to use the procedures up to several 10‘s of Hz.*

NOTE: *The Recording Procedures is based on the standard recording mode (Manual Acquisition). Before a procedure can start the recording must be active.*

1. The Recording Procedure function is an optional feature and requires the corresponding licence option.

If the hardware is activated the functions assigned to the *Recording Procedure* are available via the Acquisition menu (Figure 66).

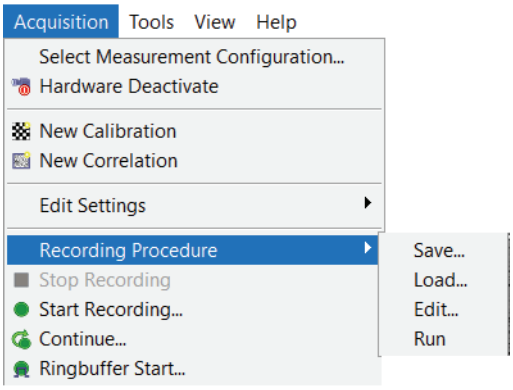

Figure 66 Acquisition Menu Recording Procedure

5.2.1 Basic Elements of a Procedure

The basic principle is that the images from the camera are streamed into the computer at the specified frame rate. To each image specific features are assigned. For example:

- image index
- time stamp
- analog input values
  optional, if the analog input is activated (*The value of the MAI should be smaller than the corresponding Analog Input Setup*)

A recording procedure contains so called *loops* and the images are streamed into these loops. A trigger event causes either a break to stop that loop (**BREAK ELEMENT**) or an action (**ITEM ELEMENT**). A *Recording Sequence* can be built out of several loops. One loop may contain multiple Break or Item Elements.

With these base elements a large variety of different automatic acquisition sequences can be built.

Break Element

A Break Element is generating a trigger to exit the loop. This trigger can be generated by the following conditions:

- **FRAME INDEX TRIGGER**
  Number of images streamed through the loop.
  If the number of images which have entered the loop reaches the defined limit the loop exits

Parameter	Description	Default
**INDEX**	Maximum number of images streamed in this loop	100

- **STORED FRAMES COUNT TRIGGER**
  Number of saved images.
  If the number of images which have been saved within the loop reaches the defined limit the loop exits

Parameter	Description	Default
**COUNT**	Maximum number of images saved in this loop	10

- **ANALOG EDGE TRIGGER**
  Edge Signal on an analog Input Channel.
  If the signal of an analog value oversteps the defined limit the loop exits. To use this function the recording of at least one analog channel must be activated (see *The value of the MAI should be smaller than the corresponding Analog Input Setup*).

Parameter	Description	Default
**CHANNEL_NAME**	Name of the analog input channel to use (selectable from list)	
**LEVEL_VALUE**	Threshold value in the unit specified in the settings of this channel	0
**EDGE**	Selects if the trigger works on a rising or falling slope (selectable from list)	

NOTE: *The values for definitions of analog signals are in respect to the transformations defined. E.g. if the value is transformed from voltage to force you have to use the value of the force and not the voltage.*

- **PHASE CHANGED TRIGGER**
  Phase Change between Sync In and Clock signal.
  It is designed for use in the DAQ Master mode with external clock (see *Acquisition Clock Setup*). If the phase change or delay between the input timing signal and the output clock signal controlling the camera acquisition has changed or obtained a defined value the loop exits.

Parameter	Description	Default
**MODE**	Selects if the trigger is active when the phase has changed or is equal to a defined value	
**TRIGGER_PHASE_DELAY**	Threshold value in the unit specified in the settings of this channel	0

Tip: *The Phase Changed Trigger option is designed for the acquisition of images following the phase stepped approach. Using this trigger method it is possible to perform measurements of objects at resonance frequencies using non high speed cameras (see Change Phase Action).*

- **TRIGGERS OR**

Build a selection of trigger elements with a logical OR link.

With the right mouse click on *Trigger* a selection of available trigger elements appears (Figure 66). These can now be added to this item.

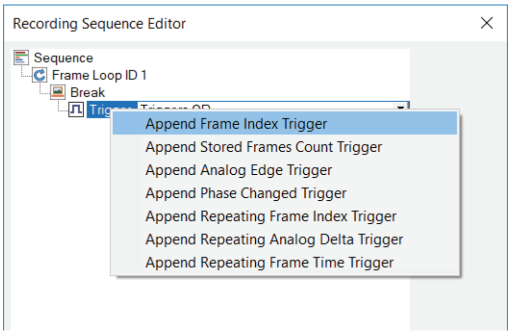

Figure 67 Acquisition Menu Recording Procedure

NOTE: *With the Trigger OR elements are more elements available as with the Break Element. These are implemented for the use with the Item Element.*

The sequence to add a Break Element is to

- append it to a loop,
- select the trigger option and
- define the trigger parameter.

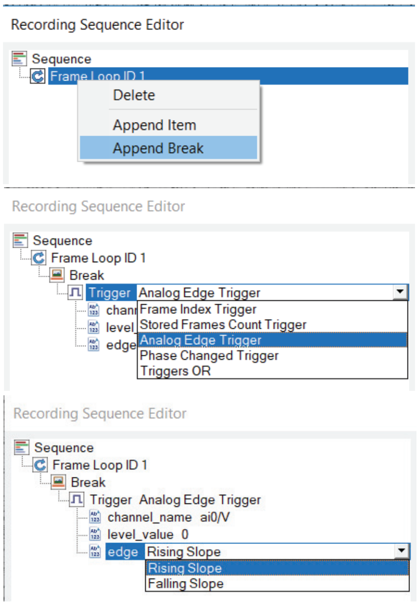

Figure 68 Recording Procedure Add a Break Element

Item Element

An Item Element is generating a trigger to perform an action. This trigger can be generated by the same conditions as a break element (see *Break Element*) and the following additional conditions:

- **REPEATING FRAME INDEX TRIGGER**
  Every n-th image starts an action

Parameter	Description	Default
**EVERY_N-TH_FRAME**	Steps in image index between two actions	10

- **REPEATING ANALOG DELTA TRIGGER**
  Defines steps of analog signals which generates a trigger. Whenever the analog value changes in positive or negative direction more than the defined delta an action is started

Parameter	Description	Default
**CHANNEL_NAME**	Name of the analog input channel to use (selectable from list)	
**DELTA_VALUE**	Change of analog input in the unit specified in the settings between two actions. The change can be positive or negative	0.5

- **REPEATING FRAME TIME TRIGGER**
  Defines the time steps when a trigger is generated. The next available image after the defined point in time is used

Parameter	Description	Default
**DELTA_TIME**	Time in seconds between the actions	1.1

- **TRIGGERS OR**
  Build a selection of trigger elements with an logical OR link.

With the right mouse click a selection of available trigger elements appears (Figure 66). These can now be added to this item.

As an action for an Item Element the following selections are available (Figure 66):

- *Storage Frame Action*
- *Change Phase Action*

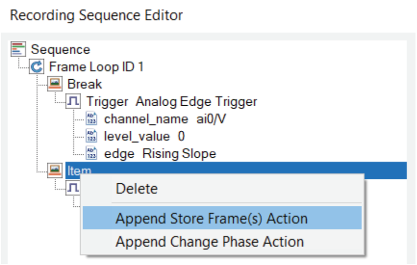

Figure 69 Acquisition Menu Recording Procedure

Storage Frame Action

A **STORAGE FRAME ACTION** is triggered by an Item Element and stores images on the hard disk.

One Storage Frame Action can store a single or multiple images. In this way multiple images can be saved on a single trigger event. It can be used as pre-(begin >0) or post-trigger (begin<0).

Parameter	Description	Default
**BEGIN**	Defines an offset of the image index relative to the triggered image	0
**COUNT**	Defines the number of images which are stored by this action	1
**STEPS**	Determine the index step of the image to be stored	1

Change Phase Action

The **CHANGE PHASE ACTION** is designed for the acquisition of images following the phase stepped approach. Using this technique the acquisition of the images is triggered by an external signal. In general this is the excitation signal of a vibrating object. If the object vibrates at a resonance frequency and the delay between the excitation and acquisition signals is constant the object is imaged always at the same state of deformation during the vibration cycle. A change of the delay will now capture the object at different position of the vibration cycle. In this way the complete cycle of an object vibrating in resonance frequency can be measured using acquisition rates much slower than the frequency of vibration.

In order to use this function the system needs to run in the *DAQ Master mode with external clock* (see *Acquisition Clock Setup*).

This action item can be used in different modes.

- *Absolute set to*
- *Relative increment*
- *Relative decrement*

Parameter	Description	Default
**MODE**	Defines what this action does (selected from a list)	
**PHASE_DEGREE**	Defines the absolute phase or the relative change of the phase	45

5.2.2 Load and Save Procedures

Using the Load and Save functions a Recording Procedure can be loaded or saved. As standard the files are saved in the actual repository. In each repository a default procedure exists.

5.2.3 Edit Recording Procedure

The Edit Recording Procedure opens a graphical editor to build or modify a Recording Sequence Procedure. If no procedure is specified the edit function opens the default procedure.

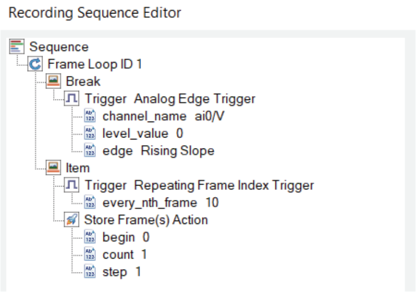

Figure 70 Default Recording Sequence Procedure

The default procedure has one loop. This loop contains a Break element which exits the loop after 10 saved images. The Item Element generates every 10th image an action. This action stores a single image.

See also *Samples of Recording Procedures* for more details.

6. Calibration

In order to evaluate the object geometry from captured camera images, knowledge of the imaging or projection parameters are required. The determination of these parameters is made in a process often called a calibration[1].The parameters describing the imaging have to be determined, i.e. the imaging parameters of each camera (Intrinsic Parameters) have to be determined as well as the relative position and orientation of one camera with respect to the other one (Extrinsic Parameters). Whenever a camera is moved or one of the camera image settings is changed (e.g. focus or aperture changed), a new projection parameter has to be determined.

It is required to perform a calibration for a 3D measurement setup (two or more cameras). In the case of using a 2D setup (one camera) it is possible to calculate the deformation and strain values without knowing the imaging parameters. In this case the information is based in pixels and the assumption that object is perpendicular to the viewing direction. Any kind of distortions are also neglected. In the case of a 2D setup **ISTRA 4D** offers the option to determine the projection parameter and take any distortion of the imaging or tilting of the object into account.

NOTE: *The calibration is always performed with the maximum number of camera pixels, independent of the selected ROI (Definition of Region of Interest (ROI)).*

Tip: *In case a proper calculation of the projection parameter, using the standard calibration procedure, is not possible, the projection parameter can be approximated by giving the known coordinates of object points. This method is broadening the use of the DIC technique to more extreme situation like civil engineering or microscopically applications. The method is based on the import of images and it's described in Import of Images for Manual Projection Calibration.*

6.1 Calibration Targets

The calibration targets used with **ISTRA 4D** are based an assembly of black and white squares. Where the intersection between the squares are the known feature positions.

1. Even though this process is in the strict sense of the word not a calibration we use this word for this process as it is commonly used in the community. We like to call the results of this process projection parameter instead of "calibration result"

*Within the **ISTRA 4D** software a list of predefined calibration targets will be installed. These targets cover a range of field of measurements from about 0.5x0.5 mm^2 up to about 1x1 m^2. They can be ordered directly with the system. In case other dimensions or specifications are required, user defined specifications can be generated.*

NOTE: *Different generations of calibration targets exist. All targets based on squares, where newer targets contain a QR-code and have serial numbers.*

The Software Version ≥ 4.4.7 can recognize the QR code and read the target specification from the QR code directly. If the target does not contain a QR code, the targets has to be selected manually.

Software Versions < 4.4.7 can not read the QR code and the specification has to be selected manually. The software can work with QR code targets as well but it is limited to the standard predefined targets.

6.1.1 List of Calibration Target

The corresponding function *Calibration Targets* in the *Tools Menu* opens a dialog with a list of the installed calibration target definition (Figure 71).

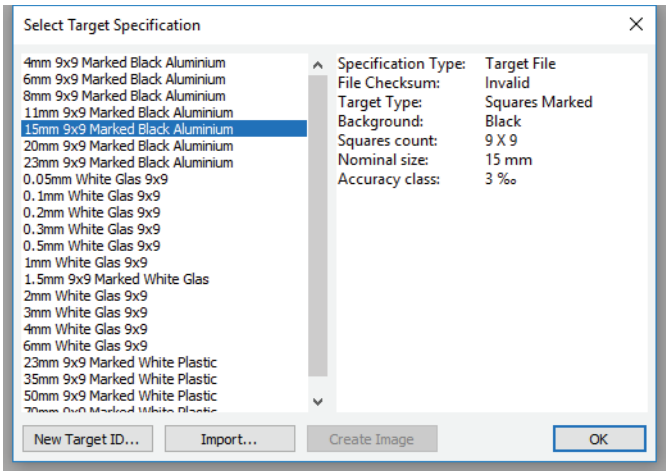

Figure 71 Dialog Select Target Specification

For predefined targets the information is read form the target specification file.

6.1.2 User Defined Calibration Targets

For the definition of a user defined target specification, a **NEW TARGET ID** is created. A dialog for the definiton of a new calibration target ID is shown (Figure 72).

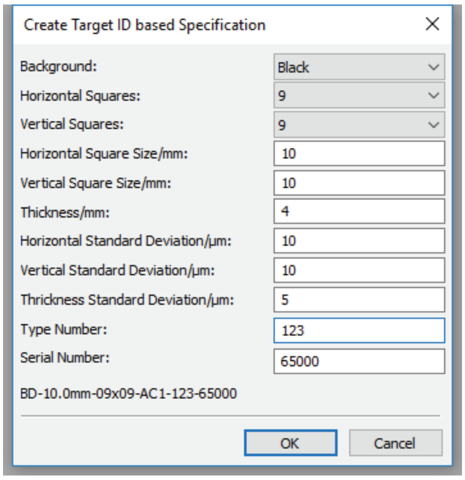

Figure 72 Dialog Create Target ID

A selection of black or white for the background color is available. The number squares in horizontal and vertical direction must be odd and can be selected from a list. The size of the squares is defined in the unit mm.

NOTE: *The size of the square in horizontal and vertical direction should be the same. However, if small deviations due to the process of manufacturing are known, the size in horizontal and vertical direction might differ < 5% of the nominal size.*

The thickness is measured from the surface of the target to the backside. It is used in case of the calibration of a 2D setup (*2D Calibration*). The standard deviations are defined from the manufacturing process and should be known. This information is used for the optimization process during the calibration.

The *Type Number* and *Serial Number* are user defined information describing the individual target.

NOTE: *The user can define Type Numbers < 1000 only. Type Numbers ≥ 1000 are reserved for manufacture use.*

The Serial Number must be ≤ 65000.

The target name, based on the selected specification, is displayed in the lower part of the dialog.

The new defined target appears in at the end of the available target specifications (Figure 73) with the coresponding features.

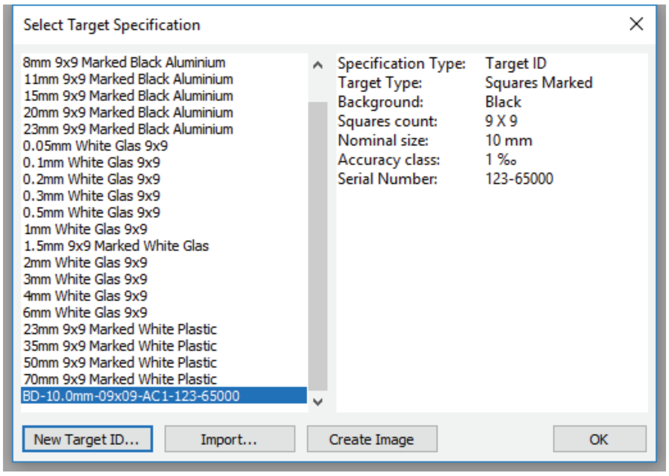

Figure 73 Dialog Target Specification New

CREATE IMAGE will open a dialog with additional parameters for the calibration target (Figure 74).

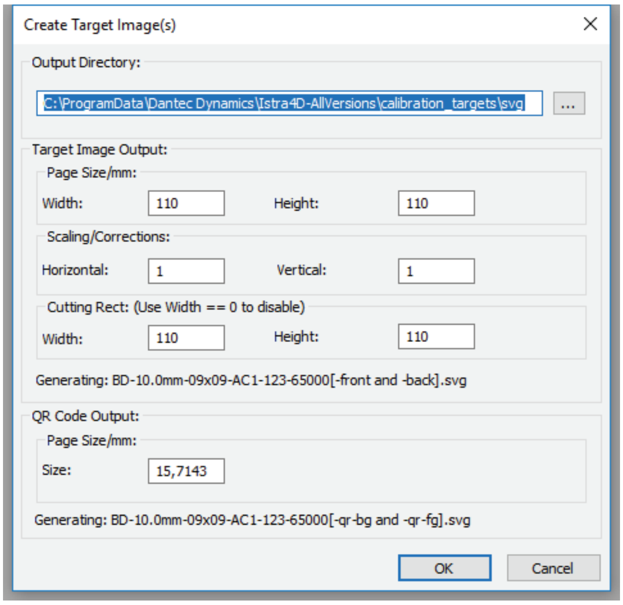

Figure 74 Dialog Create Target Images

The *Output Directory* defines the storage location of the images. The *Page Size* defines the outer dimensions of the image. By default this size is set to a minimum size.

Tip: *In case the target should be printed on paper, the Width and Height have to be changed to the paper size.*

If a scaling factor of the manufacturing process is known, the correction factor in horizontal or vertical direction can be defined, so the size of the squares of the final target match as close as possible to the defined dimensions.

Optional a frame can be drawn around the calibration pattern.

The base for the generation of the images is the Scalable Vector Graphics (SVG) format (Figure 75). To be able to produce double-sided calibration targets, the pattern for the front and back side are printed. Because some production methods require an inverted color pattern, images with inverted color are created as well.

NOTE: *If a version of the software package Inkscape (V≥ 0.92.3) is installed on the PC, in addition to the SVG files, PDF versions are generated.*

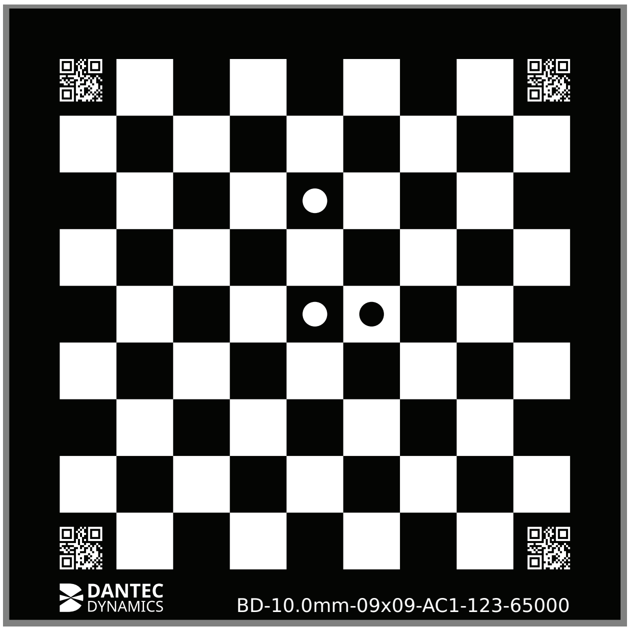

Figure 75 Example of User Defined Calibration Target

In addition images with the QR code only are generated. The default size is idetical to the size on the target, but the size can be changed.

Tip: *By generating QR codes for existing targets, the automatic detection feature of the new software can be used with existing targets.*

NOTE: *In case you use a QR code reader, the content will not be the target name, as the content is coded.*

The unique Target ID of a user defined target specification can be copied as 35 digit number into the clipboard with a right mouse click on the Target Specification. If a valid ID is in the cilpboard, a new target specification can be created, based on the data in the clipboard.

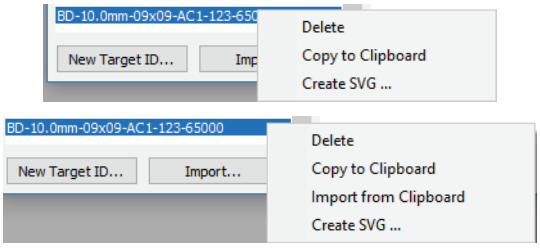

Figure 76 Copy Target ID via Clipboard

Tip: *This Target ID number can be saved in an extra list and from this number the traget specification file and target image can be reproduced anytime.*

6.2 Calibration Live Image

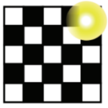

The calibration can be started by activating the *Calibration Setup* icon from the *Acquisition Toolbar*, where upon the calibration live image views are opened.

The Calibration live Image view is in principle the same as the *Live Image* view (5.1.2). The *Zoom,* and *Color Table* display work in the same way.

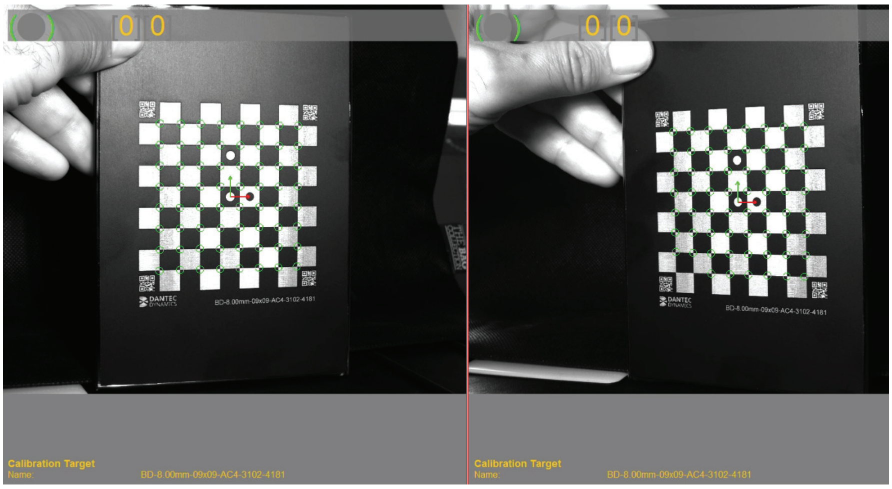

Figure 77 Calibration Live Image

In the calibration live image view a colored ball indicates the status of the calibration (*Calibration Process*).

NOTE: *The functions for the Acquisition Clock and Brightness Setup also exist in the Calibration Live Image mode. But the settings in this mode are completely independent from the settings in the Live Image View (5.1.3).*

The *Acquisition* menu changes to:

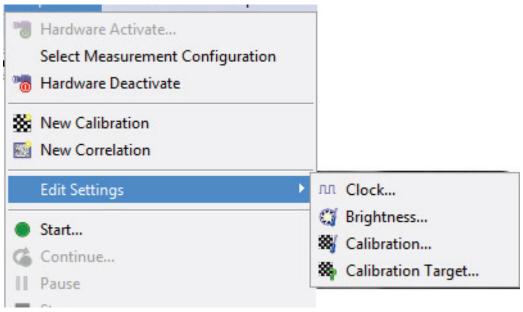

Figure 78 Acquisition Calibration Menu

**Description**	**Icon**
Acquisition Clock Setup	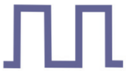
Brightness Setup	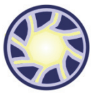
Change the Calibration Settings	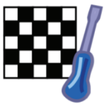
Select the Calibration Target	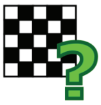

Table 8: Acquisition Calibration Settings Menu

Tip: *Because the intensity of the scene may change due to the different orientations of the calibration target it's recommended to use the Auto Gain function in the Brightness Settings dialog during the calibration process (Brightness Setup).*

6.3 Perform a Calibration

The calibration target should be in the full field view of both cameras. Choose an appropriate calibration target and hold it in the field of view of both cameras simultaneously. The software identifies the corners of the squares in the calibration pattern online and marks detected corners with circles (Figure 77).

The orientation of the calibration target is recognized by three additional circles in the center of the pattern. If the software is able to determine the orientation, a blue arrow is drawn in the live image from the central circle to the circle in the neighboring square.

The circles at the corners are drawn either in green, red or blue

Green:<br><br>Sufficient number of corners are detected	
Red:<br><br>The coordinate system is detected but not enough corners detected	
Blue:<br><br>The coordinate system can not be detected	

Table 9: Indication of found corners during on-line calibration

6.3.1 Prepare the Calibration

In order to perform the calibration procedure it is necessary to capture images of the calibration target with different orientations (*Positioning of the Calibration Target*) and evaluate the detected corners of the squared pattern. Thus before starting the calibration process, as described in *Calibration Process*, check, whether it is

possible to hold the target in different orientations (*Positioning of the Calibration Target*) with a sufficient number of corners detections (circles are displayed in green color).

Tip: *If the corners are not displayed in green, the reason may be one of the following:*

There are not enough corners in the field of view:

- *Change the target position (Positioning of the Calibration Target), but pay attention that the target stays in focus,*
- *choose another calibration target (Selection of Calibration Target) or*
- *change the calibration parameters (Calibration Parameters)*

The target is out of focus:

- *change the target position (Positioning of the Calibration Target)*

The image contrast is too low

- *change the illumination setup or*
- *change the shutter time (Setting of Exposure Time and Camera Gain)*
- *change the calibration parameter (Calibration Parameters)*

6.3.2 Positioning of the Calibration Target

The calibration target must be in the field of view of both cameras simultaneously and it has to be in focus in both cameras. Thus it should be positioned at the same distance as the measurement specimen. The target must still be in focus, even if it is tilted in different orientations.

The target should fill the camera field of view as much as possible. Choose a corresponding target size.

Tip: *If the depth of focus is sufficient, you might put the target just in front of the specimen. In most of the cases it's better to rotate the rail with the mounted cameras to a different position away from the object where there's enough space to hold the target in different positions. As long as you don't change the relative position of both cameras with respect to each other the calibration is valid.*

6.3.3 Selection of Calibration Target

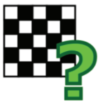

The *Select Calibration Target* icon opens a dialog for selecting the specification of the Calibration Target to be used. Choose a target to meet the calibration requirements of your setup (*Prepare the Calibration*). The available list entries usually correspond to the Calibration Targets which are delivered with the system.

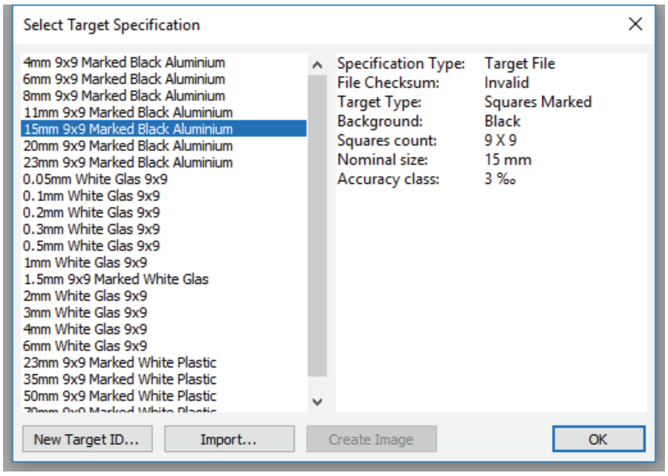

Figure 79 Select Calibration Target Dialog

The description contains the size of the square in mm, the number of squares in horizontal and vertical direction and the color and material of the background of the target.

For the naming of the corresponding files, the first two letters identify the material of the top surface of the target, followed by the pattern size (the size of a single square in mm), a (2- or 3-letters) target-specification-code and finally the number of squares in the pattern.

NOTE: *Explanation of the target-specification-code:*

1. Letter: (B or W) indicates black or white background color.

2. Letter: (M or not present) indicates whether target orientation marks are present.

3. Letter: (B or F) defines, whether the corner squares show background or foreground color.

Double Sided Calibration Targets

If Double Sided Calibration Targets are used, each target has it’s serial number printed on the target. For each target a special description file is delivered and this has to be selected for the calibration with this target.

The active type of calibration target is shown in the lower left corner of the live image.

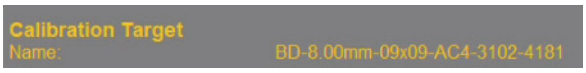

Figure 80 Selected Calibration Target

Automatic Target Detection

If a target with a QR code is used, the software can detect the target specifications automatically. At the beginning of the calibration process the target shall be position perpendicular to one of the cameras. The software will try to detect the QR codes and analyze the information. If one QR code is detected successfully, it will be marked green and the corresponding target information is displayed.

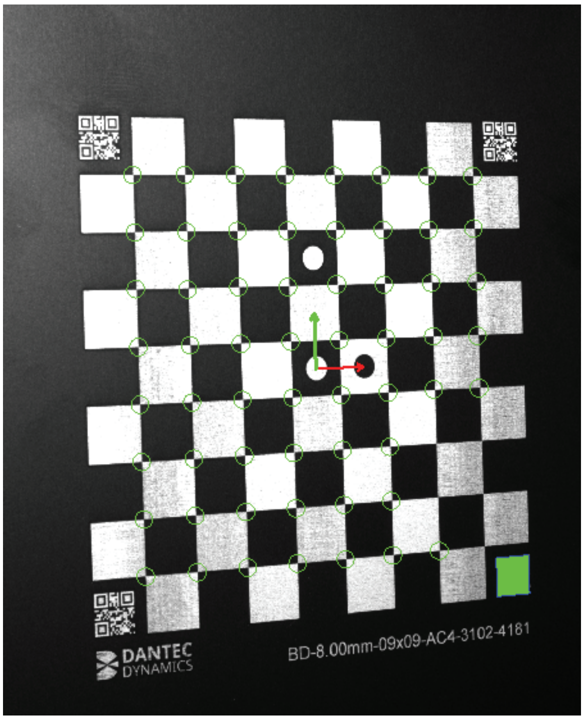

Figure 81 Calibration Target with detected QR code

NOTE: *In order to use this function, the Target Auto Detect function needs to be active and a corresponding QR code needs to be in the image.*

The QR code can be integrated in the squares of the target or printed by using the Create Image function (User Defined Calibration Targets).

6.3.4 Calibration Mode

The calibration can be done in different ways. The most common way is to calibrate both cameras at the same time. Here the Intrinsic and Extrinsic Parameters for both cameras are calculated at the

same time. For some applications it can be necessary to determine the Intrinsic and Extrinsic Parameters independently or the parameters for both cameras in a separate procedure.The calibration mode can be selected from the drop down lists (Figure 82)[1].

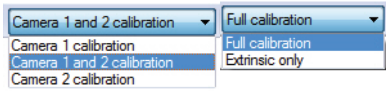

Figure 82 Calibration Mode Selection

Simultaneous calibration

In this calibration mode within one calibration all parameters are calculated. For this mode all cameras and the *Full calibration* must be activated (Figure 82).

Tip: *The simultaneous calibration is the best and easiest way to calculate all required parameters. Therefore we recommend this method as the standard procedure to calibrate the system.*

Separate calibration of Intrinsic and Extrinsic Parameters

In this calibration mode the Intrinsic and Extrinsic Parameters are calculated in two steps.

In the first step a *Full calibration* is made (*Simultaneous calibration*), the Intrinsic and Extrinsic parameters are calculated. Where only the Intrinsic Parameters for both cameras are used later.

In the second step (*Extrinsic only*) only one image of the calibration target is required and from this the Extrinsic Parameters are calculated. For this step the Intrinsic Parameters of the cameras are required. If they are still active in the memory from a calibration before they can be used or they can be imported from existing data from a file (*File-Import*).

NOTE: *The calibration target for the two steps may differ. In this mode the calibration of the Intrinsic and Extrinsic Parameters are independent. E.g. for the calculation of the Extrinsic Parameters a smaller calibration target is used. Nevertheless before the*

1. The contents of the list depends on the active hardware configuration

calibration of the different parameters is started the correct specification of the target needs to be selected (Selection of Calibration Target).

Tip: *The separate calibration of the Intrinsic and Extrinsic Parameters is useful if a full calibration is not possible because the optical conditions are changing if the position of the cameras is changed and a calibration at the position of the object is not possible.*

E.g. the system looks through a window at the object in an oven. In this case the Intrinsic Parameters can be determined without the window and after only one image of the calibration target behind the window is required.

Separate calibration of the cameras

In this calibration mode first the Intrinsic Parameters for each camera are calculated separately. Then the Extrinsic Parameters are calculated.

Each camera is selected independently from the list (Figure 78), a full calibration is made and saved in a file.

In the last step the Extrinsic Parameters for all cameras are calculated. Here the parameters for each camera are imported from the existing data from the files (*File-Import*) and a *Extrinsic only* calibration is made (*Separate calibration of Intrinsic and Extrinsic Parameters*).

Tip: *The separate calibration of the cameras is useful if a full calibration is not possible e.g. if the object is too large to use one calibration target which fills the full image of all cameras. In this case the calibration target can be moved closer to each of the cameras (they still need to be in the depth of focus) and the calibration of this camera can be made. For the calculation of the Extrinsic Parameters the calibration target does not need to fill the full field of the image.*

Calibration of large field of views

In the case of large filed of views, where no proper calibration targets are available, it is possible to generate valid projection files by using intrinsic calibration for each camera and two points at a known distance on the object. At first the intrinsic parameters for each camera have to be determined and saved (*Separate calibration of Intrinsic and Extrinsic Parameters*). For this process the calibration target doesn't need to be at the position of the object but can be moved closer to the camera, so it fills the image of the camera.

If the intrinsic parameters are saved for each camera the determination of the extrinsic parameter is performed at the start point search panel (*Startpoint search*). The user has to place two start points in the images from which he knows the distance on the

object. The start points need to be placed in the reference camera (left lower image) and needs to be set manually in the other camera (right upper image)(Figure 83).

NOTE: *The two points should have a large separation in order to allow a good quality of the result.*

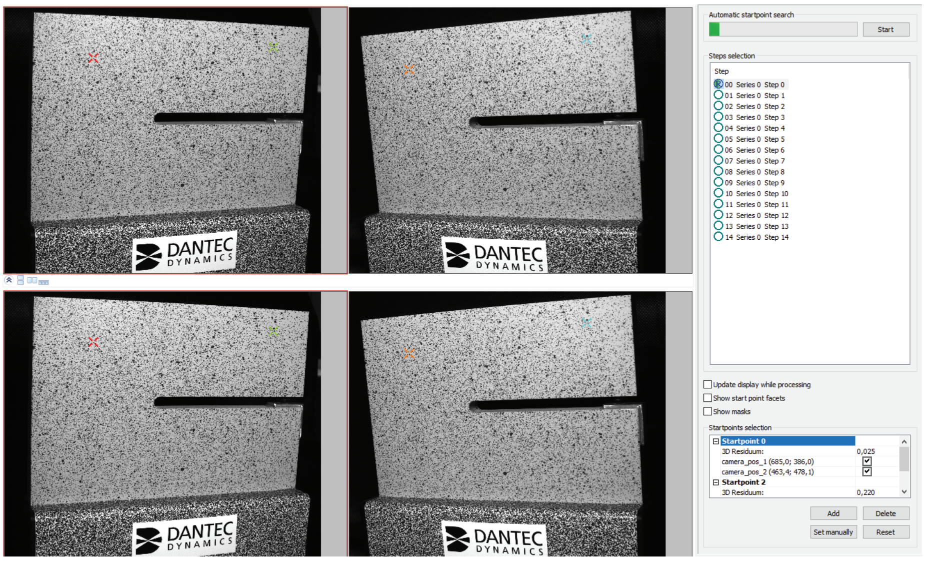

Figure 83 Calibration Set Points

A right mouse click on the step opens a menu with the entry *Determine Cameras Pose* (Figure 84).

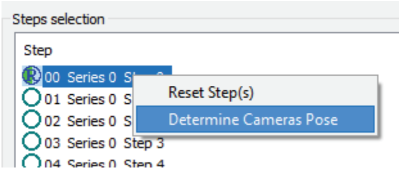

Figure 84 Calibration Determine Camera Position

Now the software will try to correlate the position of the start points and the full field data. The dialog coming up indicates the number of valid start points and the number of valid grid points (Figure 85).

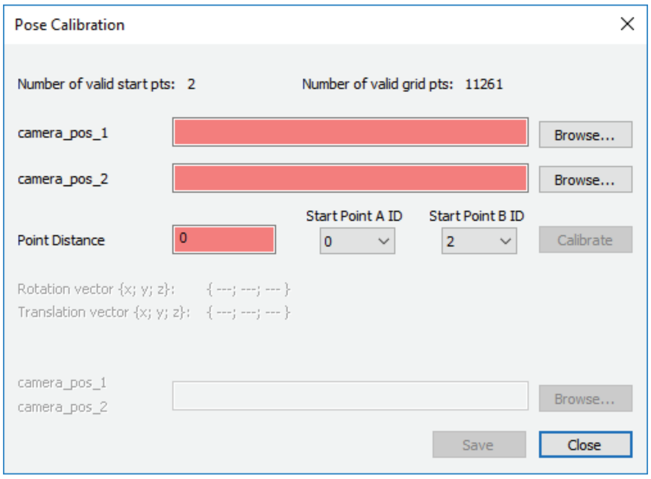

Figure 85 Calibration Pose Dialog

The name of the projection files containing the intrinsic parameters of the cameras and the distance between the two marked points on the object (in mm) needs to be entered in the dialog (Figure 86).

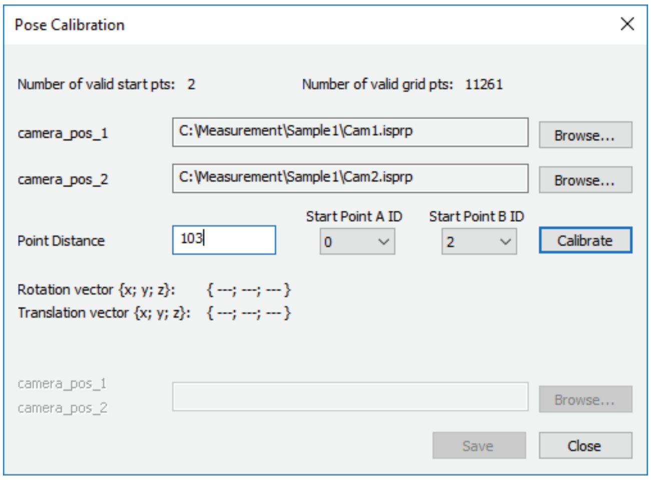

Figure 86 Calibration Pose Dialog Input

Using the *Start Point ID*’s the relevant two points are selected. Pressing the **CALIBRATE** button will start the determination of the extrinsic parameter. After a succssefull determination the complete projection parameter are saved and can be used for the evaluation process (Figure 87).

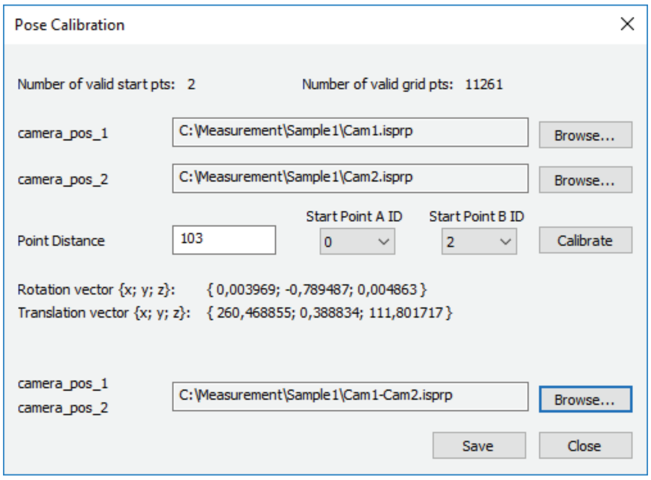

Figure 87 Calibration Pose Dialog Result

6.3.5 Calibration Parameters

The quality of the automatic corner detection can be influenced by a set of calibration parameters, which can be configured with the Calibration Settings dialog (Figure 88).

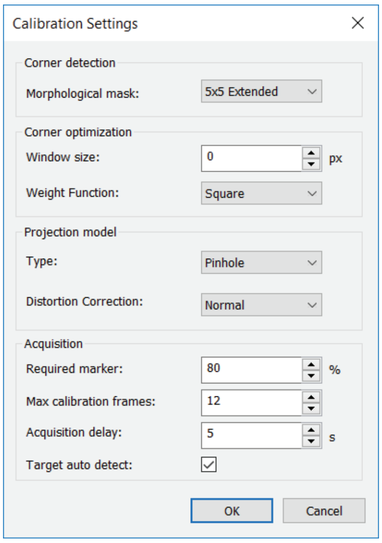

Figure 88 Calibration Settings Dialog

Tip: *The default values of the Calibration Settings are chosen in a way such that the calibration works in most cases without changes. Therefore in a situation where the automatic detection of the calibration target is not working, it is highly recommended first to try to improve as described above (Prepare the Calibration). If this does not help you may optimize the Calibration Settings for this situation.*

For the detection of the corners in the first step the images are transformed in to binary images. Then the squares are identified and the position of the corners is optimized.

The detection of the squares can be influenced by the selected **MORPHOLOGICAL MASK** in the *Corner detection* section. Here filter kernel sizes from 3 x 3 to 7 x 7 are available. For each kernel size a *normal* and an *extended* version of the filter exists.

NOTE: *The higher the resolution of the images the larger the kernel size may need to be.*

In situations with poor image quality the extended version of the filter gives better results.

Tip: *If the software is not able to detect the corners in a stable way you may increase the kernel size of the morphological filter and try the extended version of the filter.*

Once the approximated position of the corners is found an optimization is done in order to calculate the position with a higher precision.

Parameter	Description	Default
**WINDOW SIZE**	Window size for identification of corner	0
**WEIGHT FUNCTION**	Weighting of the pixel for the corner optimization.<br>*Square*: No weighting<br>*Gauss*: Gaussian weightinh	Square

NOTE: *The windows size always needs to smaller than the distance between too corners in the image. For high resolution images (> 2 MPixel) the Window Size can be increased to 9 or 11.*

Tip: *A window size of 0 will activate an automatic settings of the value, depending on the size of the calibration pattern.*

The section Projection modle configures the type of projection modle to be used.

Parameter	Description	Default
**TYPE**	Imaging modle to be used.<br>Pinhole: Pinhole modle<br>Pseudoscopic:Pseudoscopical modle	Pinhole
**DISTORTION CORRECTION**	Level of distortion parameter.<br>Normal: 2 radial and 2 tangential<br>Exdented: 3 radial and 3 tangential	Normal

NOTE: *In a Pinhole modle the entry pupille is between the object and the image; in a Pseudoscopical model the object is between the entry pupille and the image (like in many stereoscopical microscopes).*

Tip: *An extended distrotion correction might be helpfull for small focal length lenses.*

Other parameters controlling the acquisition of the images are the following.

Parameter	Description	Default
**REQUIRED MARKER**	Percentage of markers which have to be found for a valid calibration	70%
**MAX CALIBRATION FRAMES**	Maximum number of images used for the calibration	8
**ACQUISITION DELAY**	Minimum time between the acquisition of two images	4 sec

NOTE: *The number of required markers can be reduced in bad conditions but should not be smaller than 50%. Under normal conditions about 8 images is enough in order to calculate the calibration parameters with good quality, more than 15 images does not improve the quality at all.*

If **TARGET AUTO DETECT** is active, the software will search for QR codes in the image and take the information to set the target specification.

6.3.6 Calibration Process

The automatic calibration is started by clicking on the *Start Calibration* icon in the toolbar (Figure 89).

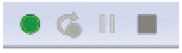

Figure 89 Calibration Process Toolbar

The icons from the toolbar allow the user to *Start* the automatic acquisition of images, *Pause* or *Stop* the process.

Description	Icon
Starts the automatic calibration process	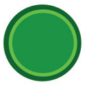
Continues the calibration	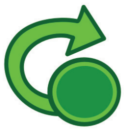

Table 10: Calibration Process Icons

Description	Icon
Pause the calibration process.	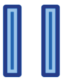
Stops the calibration process	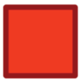

Table 10: Calibration Process Icons

A colored point in the upper left corner indicates the calibration setup. Once the calibration process has been activated the software automatically acquires the images.

NOTE: *The first captured image defines the initial coordinate system of the subsequently evaluated data (compare Calibration System). The white circular marker in the centre of the target defines the origin, the direction to the neighboring black circular marker is the X direction. This direction is indicated during the calibration with a blue arrow. The direction from the centre marker to the second white circle defines the Y direction.*

Whenever an image is acquired the display is inverted to show that the acquisition was successfully done. The number in square brackets shows the number of acquired images.

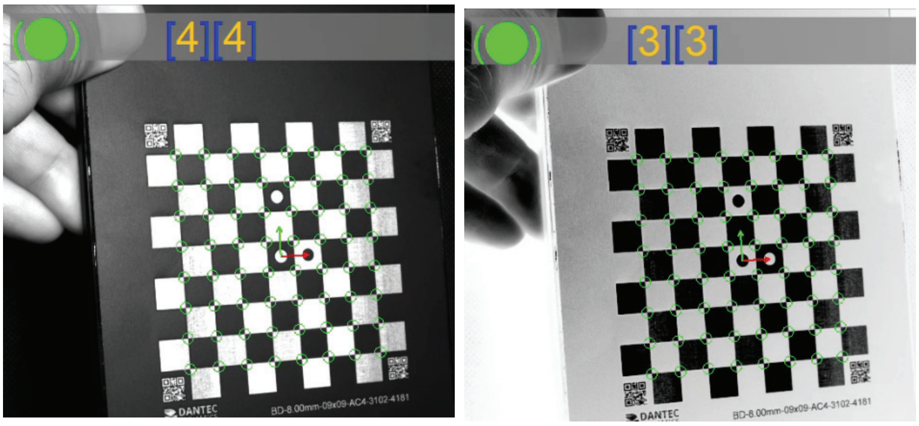

Figure 90 Calibration Acquisition of images

The color of the point shows the status of the calibration.

Grey:<br><br>Calibration setup started, but the calibration is not active.	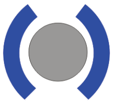

Table 11: Calibration Status Indication

Green:<br><br>The calibration is active.	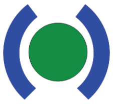
Yellow:<br><br>Attention, don’t move the calibration target because the system will try to acquire an image within the next second.	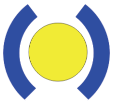
Red:<br><br>The software can't detect the necessary number of markers. Please change the position of the target in a way that more markers are detected.	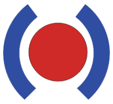
Pink:<br><br>The software is still busy processing the acquired images and calculating the calibration parameters.	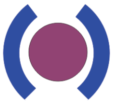

Table 11: Calibration Status Indication

The display mode of the images can be selected by drop down list (Figure 91).

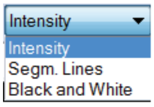

Figure 91 Calibration Display Mode Selection

As default the *Intensity* images with the found marker is shown. In case of non-sufficient quality of the marker detection, the display mode might be changed to *Segm. Lines*, where all detected lines are shown. The *Black and White* image shows the image after applying the threshold. These different display modes can help finding the optimal settings for the corner detection (see 6.3.5).

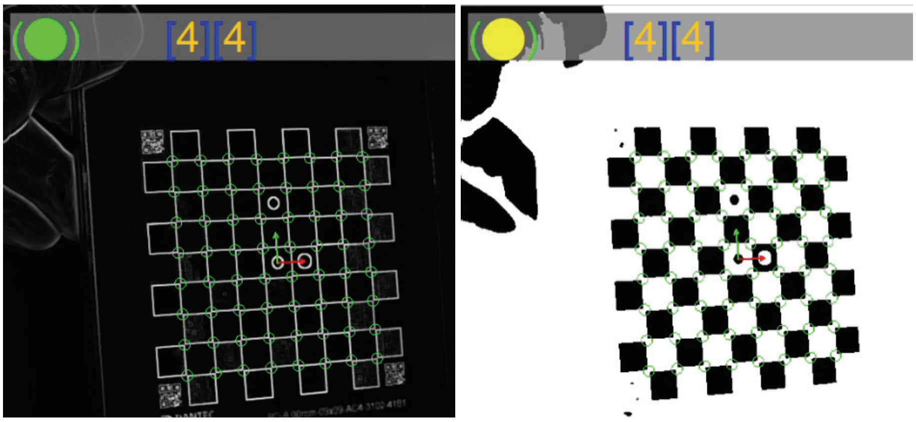

Figure 92 Calibration display mode Lines and Black and White

After four images are acquired the software starts calculating the calibration parameters and displays them in the lower part of the images.

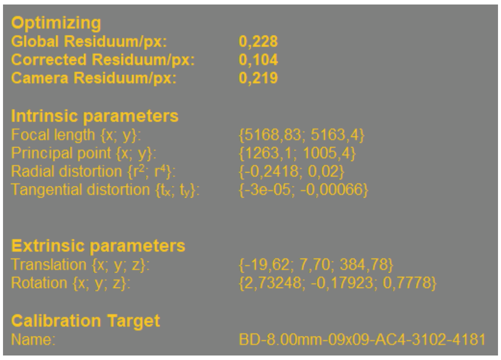

Figure 93 Calibration Parameters calculated during the calibration process

The calculation of the calibration parameters is done by an optimization algorithm. In the first section the Residuum are shown. The **GLOBAL RESIDUUM** is the average uncertainty of the found markers in all camera images in the unit of pixels. The **CAMERA RESDIUUM** is the uncertainty for the individuelle camera. The **CORRECTED RESIDUUM** is the uncertainty for the camera with the consideration of the uncertainty of the marker of the calibration target. This is a quality parameter for the calculated parameters.

NOTE: *The value for the Residuum should be less than 0.3 to 0.5 (depending on the calibration target used). If this number is more than 1.0 an error has occurred during the calculation and the calibration should be repeated.*

A small residuum is not a guarantee for a good calibration but a high residuum is an indication for a bad calibration.

Tip: *In order to get the “normal” number of the residuum for a certain calibration target it's recommended to perform three to five calibrations and look at the numbers of the residuum.*

The **INTRINSIC PARAMETERS** describe the imaging of the camera. This description is based on the pinhole model. The **FOCAL LENGTH** is separated in a X and a Y component and is the distance of the pinhole to the imaging plane. The **PRINCIPLE POINT** is the position of the pinhole in the X-,Y-Plane. The distortion is described by its **RADIAL** and **TANGENTIAL** components.

The **EXTRINSIC PARAMETERS** define the position of the camera in respect to the initial coordinate system of the first acquired calibration target image. The **TRANSLATION** is a vector between the origin of the initial and the camera origin (Principle Point). The **ROTATION** matrix determines the rotation of the coordinate system in a way that the Z-axis is in the direction of the principal axis and the X-axis is parallel to the row of pixel.

6.3.7 Calibration Result

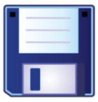

After a successful calibration process the calculated data needs to be saved. For this use the *File-Save* command from the menu or the *Save* icon from the toolbar.

A dialog appears showing the actual calibration parameters which will be saved (Figure 94). In this display the parameters for all calibrated cameras are shown. Additionally to the values of the parameters the uncertainty of each value is given.

The Stereo Parameters (**STEREO ANGLE** and **BASELINE**) are calculated and displayed below camera parameters.

If available, the calibration **TARGET** information and the **CAMERA TEMPERATURE** at the time of calibration are displayed as well.

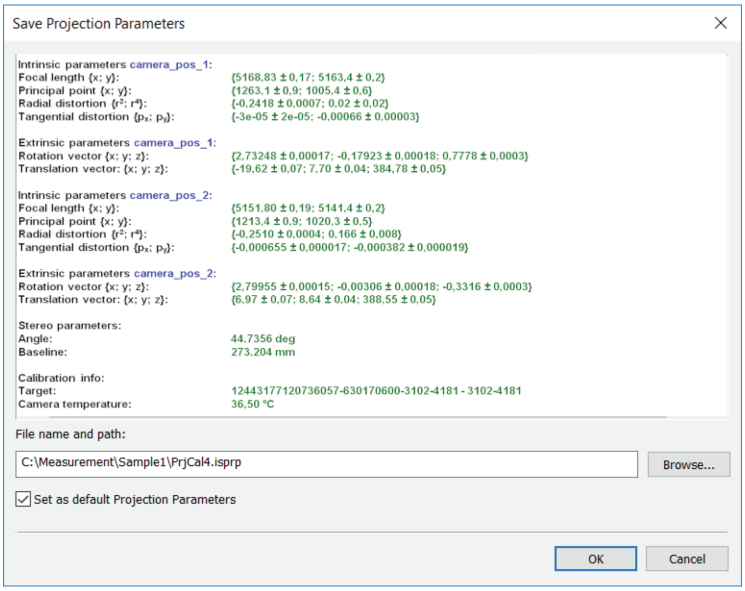

Figure 94 Save Projection Parameters Dialog

Tip: *Check the full path to make sure the file is stored in the correct directory*

Finally the user is asked if this projection file shall be used as the *Default Projection* file. This file used as the default projection file for the next evaluation (see also *Create New Evaluation*).

6.3.8 2D Calibration

Performing a 2D Calibration is similar to the 3D case. In the 2D calibration process the position of the calibration target at the first image acquisition defines the surface of the object to be measured. And therefore also the plane of deformation.

Using the full 2D calibration will give the right scaling of the measurement, correct any tilt of the object surface to the viewing direction and takes distortions of the imaging into account.

7. Evaluation

Evaluating the acquired data describes the process of determining the 3D coordinates of the object contour for each single measurement step. Each evaluation process is directly linked to a measurement data series. When a measured data set is selected the user can open a new evaluation project and a new evaluation series is created in the active repository.

NOTE: *It's possible to create from a single series of images multiple independent sets of evaluation data. Each set of data can be configured individually.*

7.1 Create New Evaluation

If a series of images element is active in the Repository Explorer (*The Repository Explorer*) a new evaluation can be started by a right click on the data set, the *New Evaluation* icon or the menu.

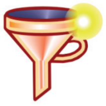

As a first step the type of evaluation needs do be defined (Figure 95).

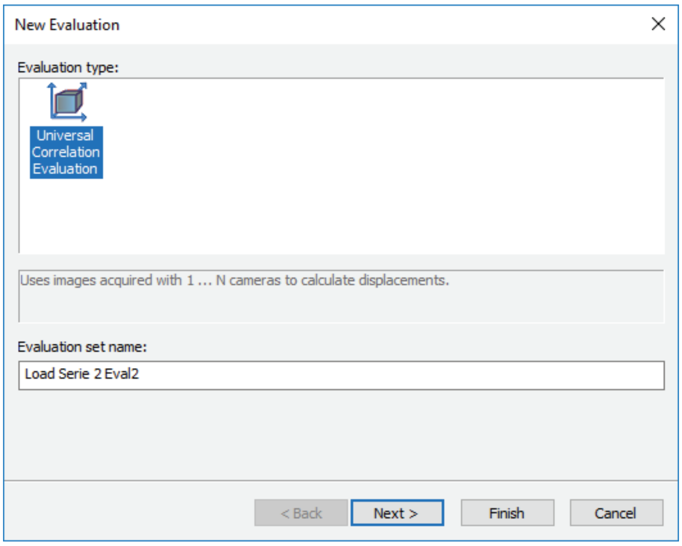

Figure 95 Dialog New Evaluation

NOTE: *In previous versions of the software in addition a 2D and 3D Evaluation were available. These are now removed and replaced with the Universal Correlation Evaluation.*

The Universal Correlation Evaluation is the most flexible way to use the evaluation process. It allows the use of

- any number of cameras[1],
- the combination of images from different measurement series
- the *One Click* evaluation
- the capability for a real-time correlation
- the output of an analog signal proportional to a real-time correlation result.

The user is guided by a wizard through the first steps.

The name of the **EVALUATION SET DATA** which appears in the *Repository Explorer* can be entered. As default it's generated from the name of the original series of images with an increasing number.

In the following step the projection parameter are selected. If defined the software uses the *Default Projection Parameter* for this evaluation (see *Calibration Result*, *Projection Parameter*). Other parameter can be selected by using the *Browse* button.

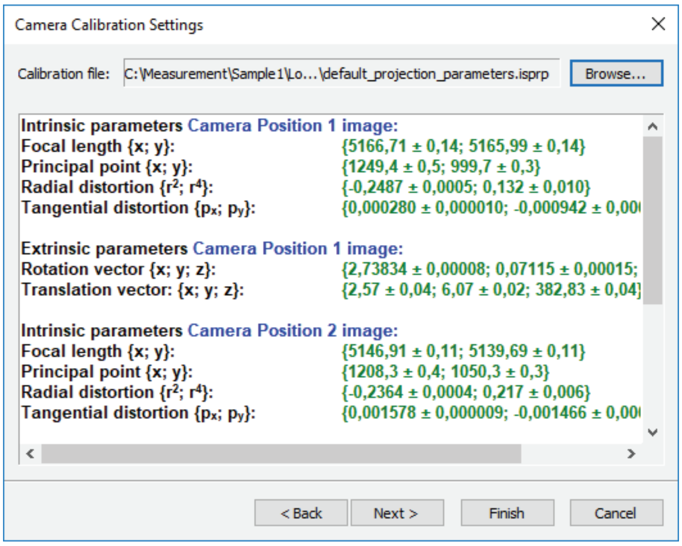

Figure 96 New Evaluation Select Projection Parameter

In the case of a 2D setup it is possible to use a simple 2D projection parameter file. If a 2D calibration is performed this projection parameter can be used.

NOTE: *The simple 2D projection parameter file is prepared for Camera Position 1 and a scaling of 1mm per pixel. The way how to change the scaling is described within the file which can be open as ascci file.*
The projection parameter file can be found at:
[Program files]\Istra 4D 4.4.7\config\projection_definitions

1. The maximum number of cameras which can be used depends on the licence

The *Next* button opens a new dialog showing a list of available cameras (Figure 97). In this the configuration of cameras used in this evaluation is defined. In the left hand list is the **SELECTABLE CAMERAS** all available cameras from the acquisition are listed. The cameras which should be used can be moved to the right hand side in the **SELECTED CAMERAS** list. The first camera in the list is the reference camera for the evaluation.

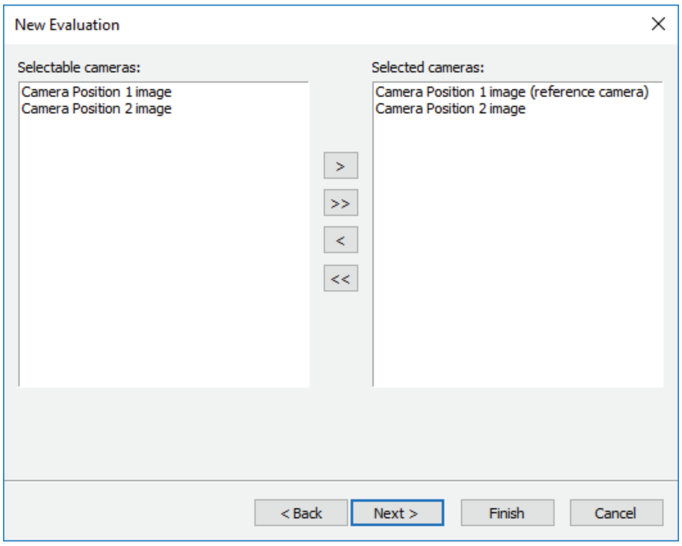

Figure 97 New Evaluation Select Camera Configuration

NOTE: *In the case of Multi Camera systems (more than two cameras) all available cameras are listed in selectable camera list.*

If only one camera is selected the system assumes a 2D[1] *measurement otherwise it will perform a 3D evaluation.*

Tip: *The camera at position 1 of the selected camera list is always the reference camera. If another camera should be the reference camera this must be in the first position of the list. E.g. removing camera 1 will bring camera 2 to the top as the reference camera. Adding camera 1 again will bring camera 1 to the second position.*

1. A 2D evaluation assumes that the deformation is in the xy plane only.

Using the *Next* button brings up a new page *Settings Transfer*. Within this page the user can copy specific settings from an existing evaluation to be used for this evaluation (Figure 98).

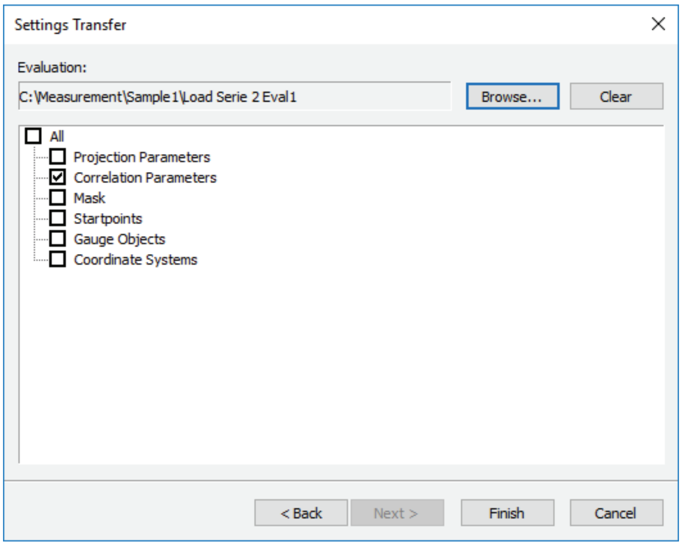

Figure 98 New Evaluation Settings Transfer

The *Browse* button in the upper area selects the directory containing the evaluation to be used as the origin of the parameters. The use of some of the parameters only makes sense if the two sets of evaluations are based on images acquired with the same measurement setup.

The following sets of parameters are available:

- *Projection Parameters*: The first available set of projection parameter of the *Input Data Selection* is used for the actual evaluation
- *Correlation Parameters*: The complete set of parameters defined in the *Correlation Settings* are used for the actual evaluation
- *Mask*: Uses the *Mask Definition* of the selected evaluation
- *Startpoints*: Copies the settings of the *Startpoint search* to the actual evaluation
- *Gauge Objects*: Uses the same objects of the *Gauge* visualization settings as defined in the selected evaluation
- *Coordinate Systems*: User defined coordinate systems are transfered to the new evaluation

The *Finish* button creates the corresponding series and entry in the repository explorer.

7.2 Start a Universal Correlation Evaluation

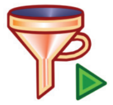

If a Universal Correlation series is activated only the *Start Evaluation* icon is accessible. This icon will display a Multi Image Control View in the center and a vertical control panel to the right (Figure 99).

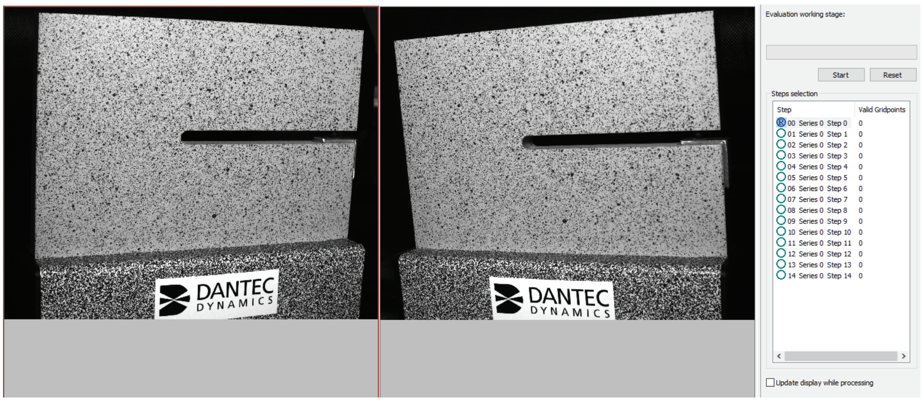

Figure 99 Control panel for Universal Evaluation

This control panel is used to perform the whole evaluation process. In the lower part the different evaluation parameter and steps are accessible by the controls. Depending on the active control in the upper part different elements are displayed.

The following controls are available (Figure 100):

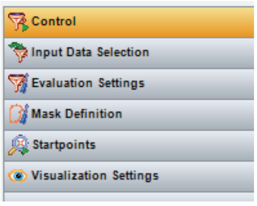

Figure 100 Controls for Universal Evaluations

In order to gain more space for the display of the elements of each control the user can drag the list down. In this case only the icons of the different controls are shown in the lowest element (Figure 101).

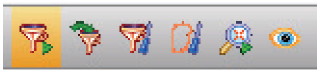

Figure 101 Controls for Universal Evaluation (minimized)

In the following the different controls are explained in detail.

7.3 The Control panel

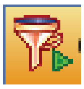

This is the main control and allows the user to start directly a complete evaluation process, the *One Click Evaluation*. In the upper part information about actual status of this evaluation is given (Figure 102).

If the default parameter are sufficient to perform the evaluation the button beside the upper progress bar is set to *Start*. Otherwise it's set to *Error*.

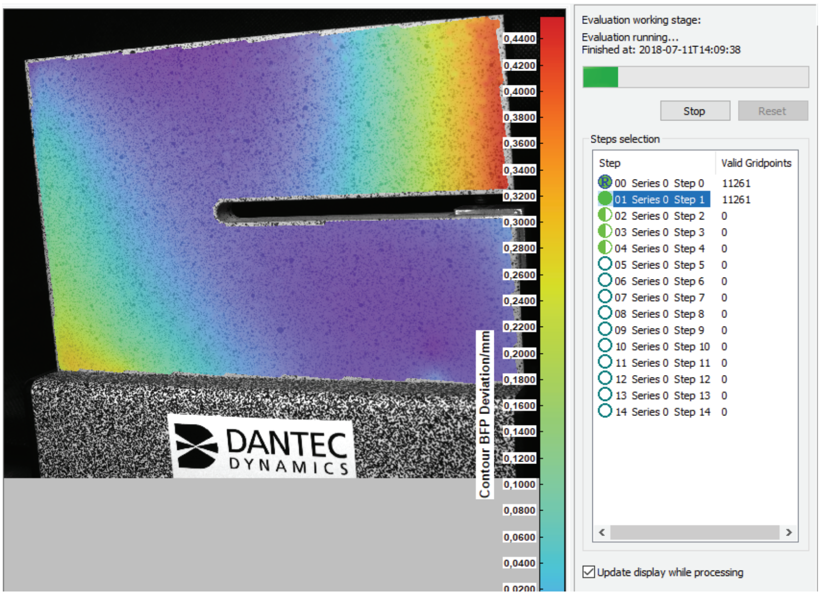

Figure 102 Universal Correlation Evaluation Control

Activating the evaluation all steps are correlated and the results are displayed as 2D overlay to the camera image in the Multi Image Control View. As default the distance to a best fit plane is shown. The status bar indicates the progress. If a step is finished the number of successfully solved facets is indicated.

If the **UPDATE DISPLAY WHILE PROCESSING** is ticked the display is updated when steps are evaluated correctly.

Tip: *If the data can't be evaluated most probably the projection parameter are not set (see Correlation Settings) or the start point might not be on the object*

7.4 Input Data Selection

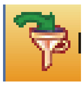

In this section the images and steps included in the evaluation series are selected. Also the projection parameters are defined here (Figure 103).

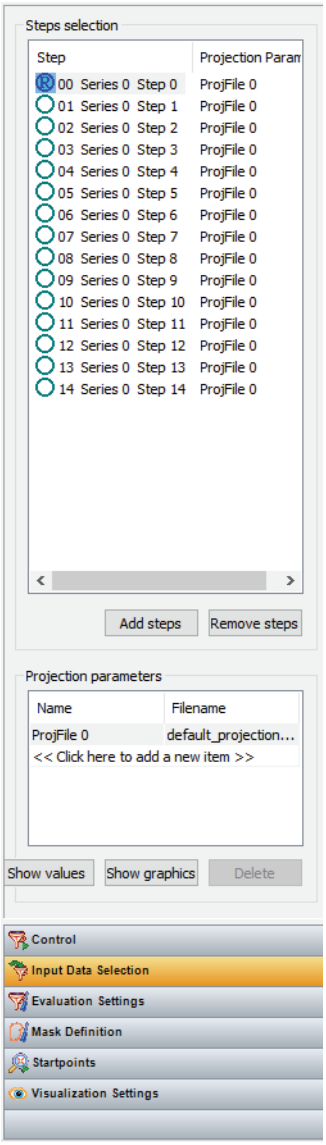

Figure 103 Universal Correlation Evaluation Input Data Selection Control

In the upper part a list with the steps and the linked projection parameter is shown. A right mouse click on each step opens a menu (Figure 104). Here the type of the step as well as the projection parameters can be defined.

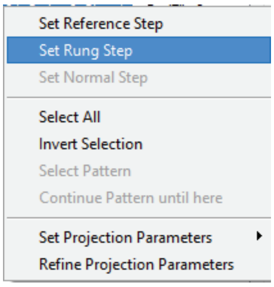

Figure 104 Menu Type of Evaluation Steps

A step can be one of the following types:

- *Reference Step*: This step is used to correlate the images between the cameras. Each evaluation series should have one Reference step only. This step is marked as *R* with a blue circle.

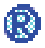

- *Rung Step*: This step is a sub-reference within the images of one camera. All following steps are correlated to this step and to the reference step. Each evaluation series can have multiple *Rung Steps*. These steps are marked as *R* with blue background.

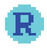

- *Normal Step*: Every step which is not a kind of reference step is called a normal step.

NOTE: *For each evaluation a single step is the reference step. In the images of the reference camera of this step the mask and the measurement grid are created. For this step also the correlation between the reference camera and the others is done. For all other steps the images of each camera are correlated to the image of their reference step. If a sub-reference step is defined the following steps are correlated to that step.*

Tip: *In case of large displacements it might be advisable to include further sub-references, as the accuracy of the correlation algorithm decreases with larger displacements. A sub-reference serves as a refresh: When a sub-reference is passed, each subsequent step will be correlated to that sub-reference. As a matter of error propagation statistical uncertainties of the resulting object contours increase with each sub-reference included. Therefore sub-references have to be used with care.*

All Steps selects all steps in the actual list. *Invert Selection* invertes the actual selected steps. Using *Select Pattern* continue the actual pattern of selection until the end of the list. *Continue Pattern until here* continues the pattern until the actual position.

NOTE: *The steps should be selected first, as second action the function is selected.*

Tip: *If you e.g. every 10th step shall be selected, mark step0 and step 9 and use the Select Pattern function.*

The entry *Set Projection Parameter* offers a list of available projection parameter. These can be linked to the selected evaluation step.

The option *Refine Projection Parameters* optimizes the projection parameter by reducing the value of the 3D Residuum for all data points.

When finishing the optimization the statistics of the 3D residuum before and after the optimization are displayed (Figure 105). By comparing the values the success of the refined process can be estimated.

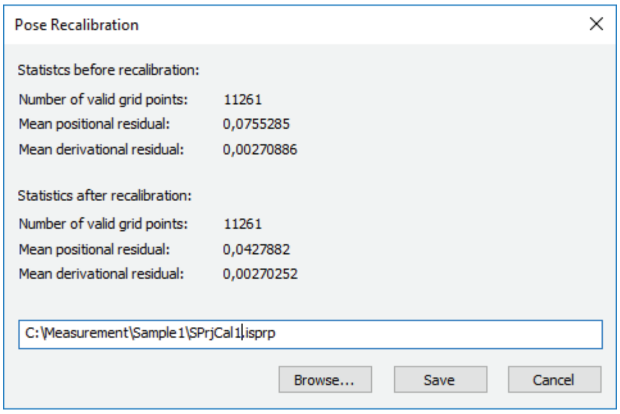

Figure 105 Dialog Recalibration Results

The Browse and Save buttons allow the storage of the new projection parameter. To replace the actual used parameter by the refined ones these parameter have to be loaded in the corresponding *Input Data Selection*.

NOTE: *The refining of the projection parameter requires the present of an evaluated data sets. The principle of this function is the rotation of one camera in order to reduce the 3D residuum for the entire data set. This function is meant to be used if by mistake one camera is slightly rotated. This function does not replace a calibration process.*

Using the *Add Step* button a new dialog opens (Figure 106). In the section **MEASUREMENT SERIES** different sources for the images can be defined. The section **STEPS** shows all selectable steps within the selected series.

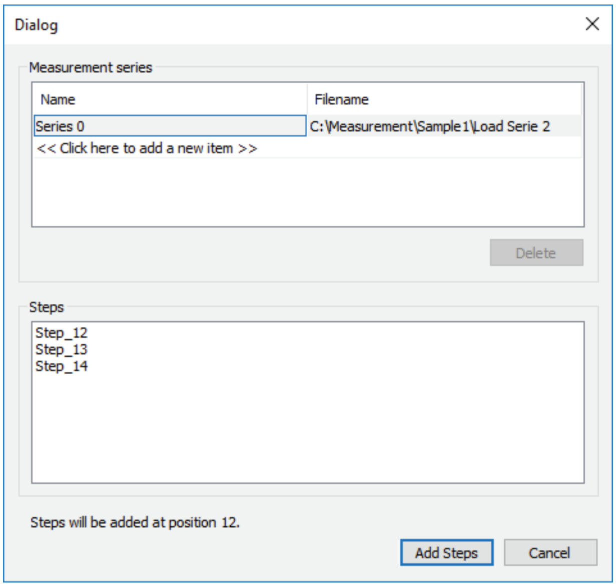

Figure 106 Dialog Add Evaluation Step

The selected steps will be placed before the active step in **STEPS SELECTION**. If no step is active the added step is placed at the end of the list.

The *Delete* button removes the selected step from the list.

The section **PROJECTION PARAMETERS** contains the list of available sets of projection parameter files. Each file referred as *ProjFile* plus a number. If a global projection file is defined the filename is set to *default_projection*. A click in the list element for the filename allows the selection of the projection file using a file open dialog.

If a projection parameter file is not linked to a step it can be removed from the list using the *Delete* button.

A dialog showing the Projection Parameter is displayed with the *Show Values* button (Figure 107).

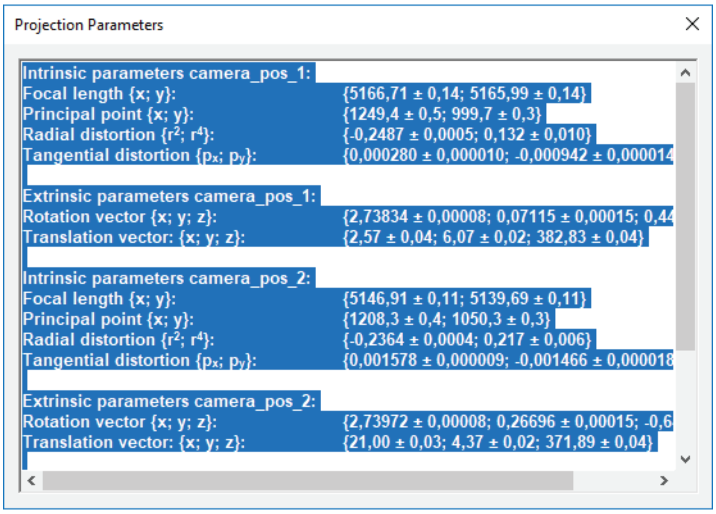

Figure 107 Dialog Projection Parameter

For every camera the *intrinsic* and *extrinsic* parameter are listed. In case of a two camera setup the *Stereo Parameter*, like the *Angle* between the optical axis of the cameras and *Baseline*, as the distance between the principal points of the cameras are displayed.

The *Show Graphics* display the setup of the cameras and the first pose of the calibration target as 3D graphic (Figure 108).

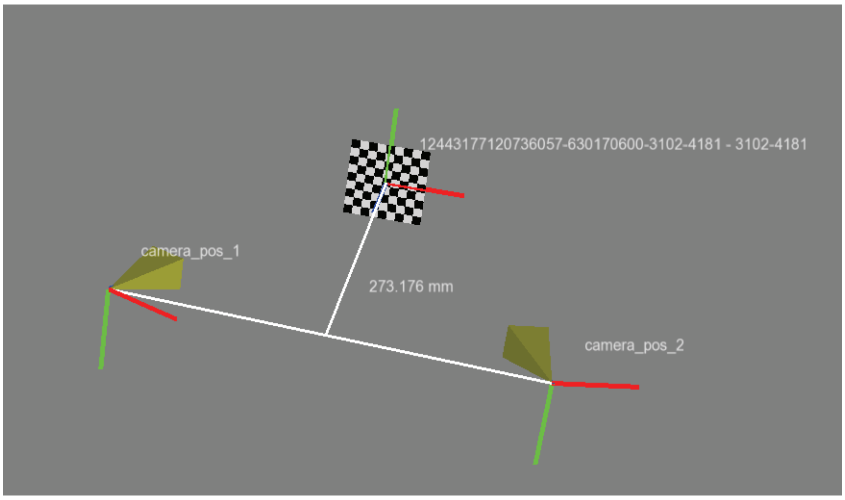

Figure 108 3D Setup of cameras and calibration target

7.5 Correlation Settings

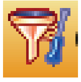

In this section the parameter controlling the correlation are defined (Figure 109).

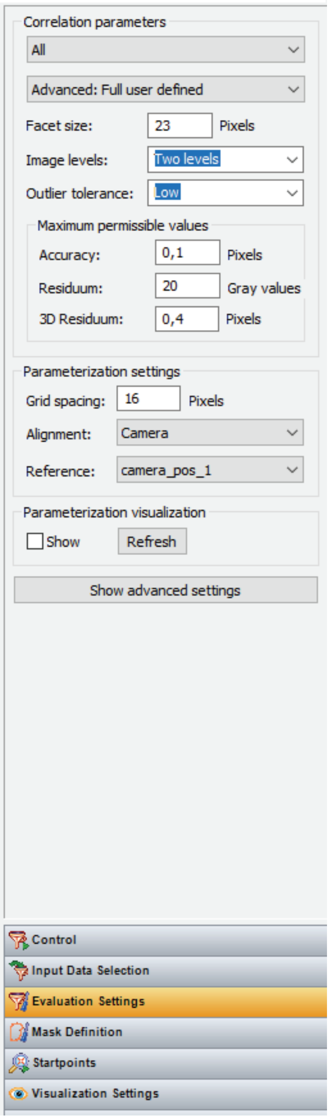

Figure 109 Universal Correlation Evaluation Settings Control

For evaluation a regular grid with equidistant grid points is laid over the image of the reference camera at the reference step. Each grid point defines the center of a squared image region in the reference image, called facet. The image correlation algorithm uses these facets for determining the 3D-position and tangential plane of the underlying object surface for each of the grid points.

The section **CORRELATION PARAMETERS** contains information controlling the correlation of each individual facet.

The first drop down list shows the different types of correlation. For each type the parameter can be set individually. If *All* is selected the parameter for each correlation are identical. The different types of correlation are:

- *Start Points Correlation*: At first the start points are searched for all images of the series. The parameters for this correlation are defined here.
- *Intra Step Correlation*: This type relates to the correlation between images of different cameras within one step like at the reference step. It does not apply to the start point search.
- *Inter Step Correlation*: This type relates to the correlation between images of a single camera at different steps. It does not apply to the start point search.
- *Surface Approximation Correlation*: These settings are used for the *Multi Camera* cluster approach only.

Tip: *In the case that wrong startpoints are found it is recommended to use stricter parameter for the startpoint search and more tolerant for the other correlations.*

In the second drop-down list the user can select predefined sets of parameters: These predefined sets are:

- *Advanced - Full User defined*: All settings can be defined by the user.
- *Normal - Good images*: These are default parameters for images with good conditions.
- *Normal - Bad images (reflexes)*: These parameters are used for images which are made under non optimized conditions like reflections in the field of measurement.
- *Fast*: The parameters are changed to optimize more the speed of evaluation rather than the accuracy.
- *High Accuracy*: The parameters are changed to optimize the accuracy of evaluation rather than the speed. This requires a good image quality.

With the use of the Advance selection all parameters are accessible.

The facet is a square area around the grid point which is used from the correlation algorithm in order to identify the corresponding area in another image. The length of the facet is set by the parameter **FACET SIZE**.

NOTE: *Facet size is an odd number and limits the spatial resolution as well, as the result of each grid point follows from the image correlation of the extended facet region. Thus increasing facet size causes smoothing of the results and decreases the spatial resolution.*

In order to have independent data points the facets of neighboring data points should not overlap. Nevertheless simulations have shown that a overlap of 1/3 of a facet does not influence the spatial resolution.

The minimal facet size is limited by the size and roughness of the stochastic pattern on the object surface. Each facet must contain a characteristic part of the pattern with good contrast features.

To ensure that the correlation finds a global minimum in general the algorithm is used on different levels of spatial feature resolution of the images. The more levels used the better the algorithm can handle larger displacements. **IMAGE LEVELS** selects the number of levels to use.

Tip: *A smaller number increases the speed of the correlation. On the other hand it increases the chance of non or false results. For the Start Point search the number should be Two Levels.*

The **OUTLIER TOLERANCE** selects the influence of the noise on the correlation algorithm. Possible choices are:

- *Low*: Noisy and outshines points have high influence on the correlation result and may generate inaccurate or non valid results. This method requires good image quality. This is the default setting.
- *Medium*: Noisy points have less influence on the correlation result.
- *High*: Noisy points have a small influence on the correlation result. This method helps for images with reflections.

NOTE: *If the Outliner Tolerance is Medium or High the evaluation time is increased dramatically*

The section **MAXIMUM PERMISSIBLE VALUES** defines the abort criteria of the correlation algorithm. A correlation must fulfill the parameters with at least the given values to be valid.

Parameter	Description	Default[a]
**ACCURACY**	Set maximum acceptable value for the correlation accuracy in pixel for each facet	0.1 Px
**RESIDUUM**	Set maximum acceptable value for the residuum of the correlation algorithm in grey values	20

**3D RESIDUUM**	Set maximum acceptable deviation of the pixel position found by the correlation and the back projected object point in pixel	0.4 Px

a. The default values refer to the *Normal Good Image* selection

The section **PARAMETERIZATION SETTINGS** specify the position of each facet.

The **GRID SPACING** is the distance between the grid points in pixel and therefore sets the spatial resolution or the distance between the data points on the object. The grid is equidistant in vertical and horizontal direction.

NOTE: *Grid spacing limits the spatial resolution, as each grid point represents one single data point of the result.*

From the attributes of the parameters grid spacing, facet size the following rules of thumb can be derived:

lower grid spacing **<->** *higher spatial resolution*

*lower facet size***<->***higher spatial resolution*

facet size = 4/3 grid spacing

facet size>> stochastic pattern size

Tip: *To improve the number of valid resolved facets it's recommended first to increase the value for the Correlation Residuum. Depending on the contrast of the stochastic pattern the best value for this parameter may change.*

The **ALIGNMENT** settings assign how the position of the facet is determined. In the conventional way a regular grid with equidistant grid points is laid over the image of the reference camera at the reference step. Each grid point defines the center of a squared image region in the reference image, called facet. The image correlation algorithm uses these facets for determining the 3D-position and tangential plane of the underlying object surface for each of the grid points. Here the **ALIGNMENT** is set to *camera*. Here **REFERENCE** selects which camera is used.

In a *Multi Camera* system the cluster approach can be used to combine the images of all cameras on the image level. In this case a reference camera doesn’t exist and the position of the facets is defined on the object surface. The **ALIGNMENT** parameter is set to *object* and the **REFERENCE** list selects the base shape of the object. This type of evaluation is called *Multi Camera Evaluation*[1].

Available shapes are:

- *Plane*: a plane is used as reference object. All cameras are expected to be on the same side of the object.

  Typical objects are large structures like wings

1. This option is only available with the *Multi Camera Evaluation* license

- *Cylinder*: a cylinder is used as reference object. The cameras are expected to look from outside on the object.

  Typical objects are tires
- *Biplane*: a plane is used as reference object. It is expected that cameras are looking from both sides (front and back side) on the object.

  Typical objects are tensile samples
- Sphere: a sphere is used as reference object. The cameras are expected to look from outside on the object.

  Typical objects are balloons

NOTE: *In case of using alignment to an object shape additional options are available in the visualization*

The section **PARAMETRIZATION VISUALIZATION** enable the display of the actual used grid points (yellow cross) and facets (green figure) on the image of the cameras at the reference step (Figure 110).

NOTE: *If the data set is not yet correlated the grid points and facets are only available for the reference camera.*

The **SHOW** selection turns the display on/off and the *Refresh* button updates the actual points and facets if the values are changed.

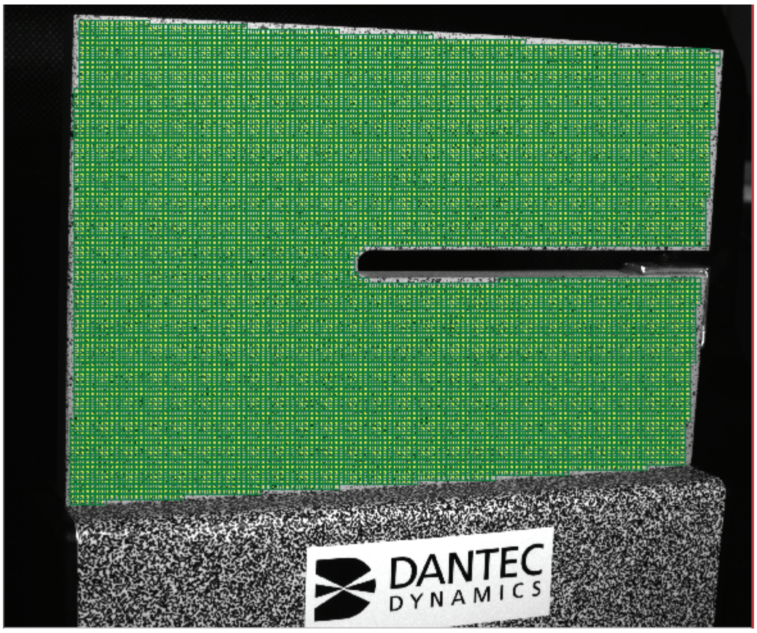

Figure 110 Parametrization showing grid points and facets

Tip: *The Parametrization visualization helps the judgement of the grid and pattern density on the object in relation to the actual correlation settings (Figure 111).*

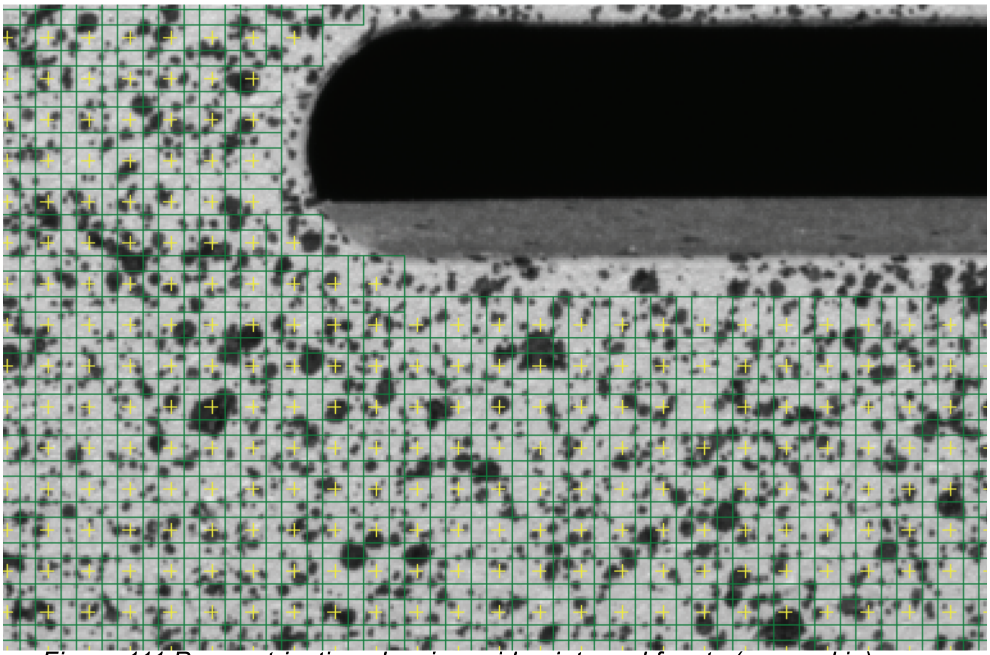

Figure 111 Parametrization showing grid points and facets (zoomed in)

The **ADVANCED SETTINGS** button toggles between show and hide of additional settings controlling the start point search and use of computer resources mainly (Figure 112).

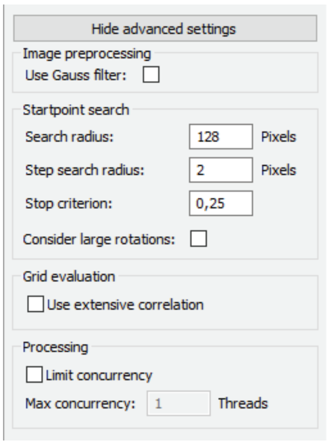

Figure 112 Universal Correlation Evaluation Advanced Settings

The section *Image Preprocessing* controls functions which are applied on the images before the correlation process. By activating *Use Gaussian filter* all images are filtered by using a Gaussian low pass filter of a sigma of 0.3 pixel.

Tip: *Using a low pass filter might reduce the effect of aliasing caused by too small pattern size.*

The section *Startpoint search settings* defines limits used during the start point search. Starting from the last valid position the correlation algorithm tries to find the next position within a certain radius defined in **SEARCH RADIUS**. Not every, but every **STEP SEARCH** point used for the search. If a position is found where all correlation criteria's are below the **STOP CRITERIA** multiplied with the maximum allowed criteria the search stops at this point and takes it as result.

NOTE: *If the movement of a start point between two consecutive images is larger than the search radius it can‘t be found.*

Tip: *If the start point has moved a bit between two steps and the Stop Criterion is too high, the start point search might find a similar but wrong start point close to the position of the previous point. In this case the algorithm does not search further for the better position.*

A reduction of the Stop Criteria will overcome this issue but increases the time for the start point search as well.

In case larger rotations exist between two measurement steps, the **CONSIDER LARGE ROTATIONS** should be activated. In this case, the start point search is extended and allows lager rotations between two steps.

The section *Grid evaluation* allows the activation of an **EXTENSIV EVALUATION**. In case of Multi Camera measurements, where more than two cameras can contribute to one data point, this evaluation analyze all combinations from the cameras for the best result.

Tip: *The extensive evaluation is recommended for the use of complex shaped objects, where some cameras might look obliquely to the surface of the object.*

In general the software tries to use as much computing resources as possible. In the section *Processing settings* the maximum number of threads used can be limited by ticking the **LIMIT CONCURRENCY** and set the value of **MAX CONCURRENCY** to a specific value.

Tip: *It might be required to limit the maximum threads if you plan to evaluate more than one data set at one time*

7.6 Mask Definition

In general the camera field of view is larger than the region of interest for evaluation of the object contour. Besides the possibility to reduce the size of the field of view during the acquisition of the images (*Definition of Region of Interest (ROI)*), a mask can be used as an advanced tool for interactively defining portions of the field of view as evaluation areas. Each of these areas is defined by a closed multi line border. Multiple distinct areas can be defined. You can also build an overlap between different areas. In this case the union of all overlapping areas will be treated as a single, connected evaluation area.

NOTE: *In the Universal Correlation Evaluation the definition of a mask is not required. Without a mask the algorithm correlates as far as possible. This might generate noise at the border or edges of the object.*

In the control in the upper part the *Step Selection* list shows all available steps for the current evaluation. The image of the activated step is displayed in the Multi Image Control View (Figure 113).

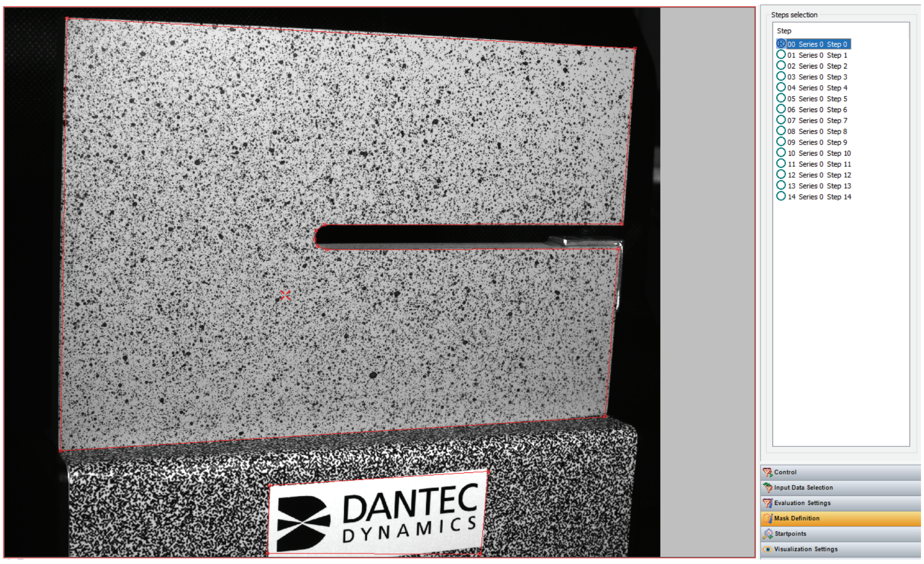

Figure 113 Universal Correlation Evaluation Mask Control

The mask is defined in the reference step on the image of the reference camera.

Tip: *If the series is already evaluated the results are displayed as an overlay on the images. In this case the mask can be changed according to the evaluated results.*

In the icon bar additional icons control the mask editor.:

Description	Icon
Swap between show and hide mask	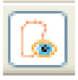
Swap between activate and deactivate mask edit mode	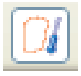
Change existing elements	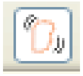

Table 12: Control Mask Edit Icons

Description	Icon
Create new polygon	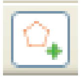
Create new marker	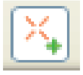

Table 12: Control Mask Edit Icons

Change Mask Visibility

Using this icon the visibility of the mask elements is switched between visible and not visible. The color of the mask elements can be changed using the corresponding function from the **ISTRA 4D** Options function (see also *Mask Editor*).

Change Mask Element

If the change of mask elements is activated depending on the position of the cursor the cursor changes the appearance to indicate the possible actions.:

The cursor is at non element.<br>*Left Mouse*: move the image<br>*Right Mouse*: no action	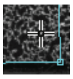
The cursor is on a line.<br>*Left Mouse*: adding a new corner, which can be dragged<br>*Right Mouse*: no action	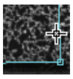
The cursor is on a corner element.<br>*Left Mouse*: drag the corner<br>*Right Mouse*: menu with functions to delete the element or polygon	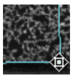

Table 13: Control Mask Change Elements

Create new Polygon

A new polygon can be created. The Left Mouse Click sets new elements, the Right Mouse Click will end the process and close the polygon.

Create new Marker

In order to complete the initialization of an evaluation area with a given mask, a marker must be set to the interior of the area. Set the marker by clicking on an arbitrary position in the interior of the polygon.

NOTE: *Each area must have at least one marker.*

Tip: *It does not matter, at which position of the interior of the polygon is set. Nevertheless: These markers are taken as default Start-Point positions for the subsequent Start-Point search. Thus it might be useful to set the markers to prominent positions where you don't expect big deformations*

Saving and Loading of a mask

The mask is saved automatically using a default name in the directory of the data. There is no need to save the mask but it is possible to use the command from the File menu or the save icon from the toolbar to save the mask using a user defined name.

Instead of creating each time a new mask it's possible to import an existing mask. If you are creating a mask you can use the File-Import command from the menu to load an existing mask definition from the hard disk.

7.7 Startpoint search

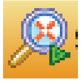

The *Startpoint Search* describes the first part of the image correlation process. For each evaluation area there must be defined at least one *Startpoint*. By default the Startpoint is in the center of the image or at the position of the marker from the mask (*Create new Marker*).

In the view two multi image controls are displayed. In the lower one the images from the reference step are shown. In the upper one the images of the active step (Figure 114).

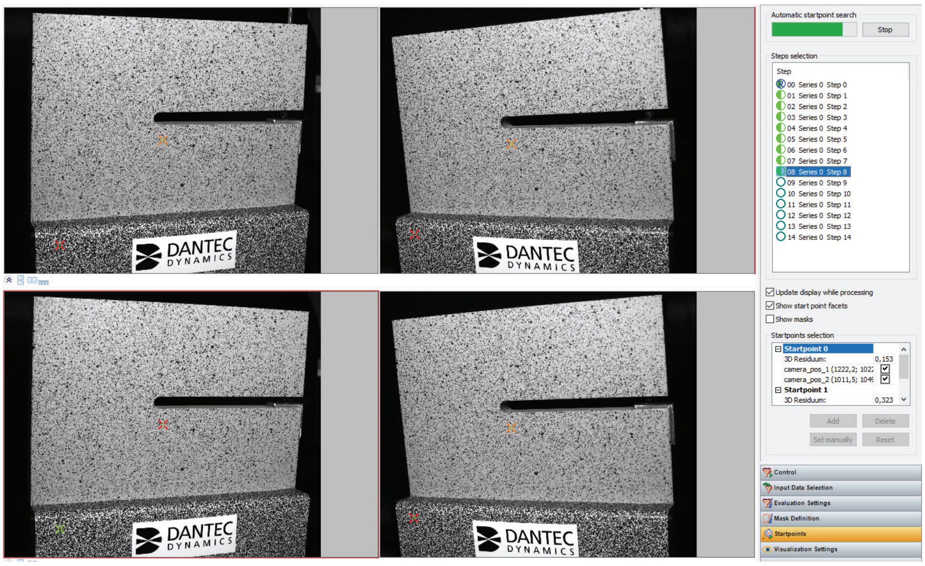

Figure 114 Universal Evaluation Startpoints

The **START** button in the *Automatic Startpoint Search* activates an automatic search of the Startpoints. The status bar shows the progress during the search. During the search the process can be stopped by using the **STOP** button.

The status for the individual steps is shown in the Steps Selection section. If a Startpoint for a step is found the leading circle is half filled. The color indicates the quality of the found Startpoint.

- *Green:* Startpoints are found with good quality (within the quality criteria defined in the correlation settings, *Correlation Settings*)
- *Red*: The quality of the found Startpoints is not sufficient. The user must check the position of the found Startpoints. If no Startpoint can be found with adequate quality the user must check the calibration data.

If **UPDATE DISPLAY WHILE PROCESSING** is active the images in the upper view are updated during the startpoint search.

The option **SHOW START POINT FACETS** draws the facet around the position of a found start point. By using the zoom function (8.1.2) the user can check the size of the selected facet size against the pattern on the object (Figure 115).

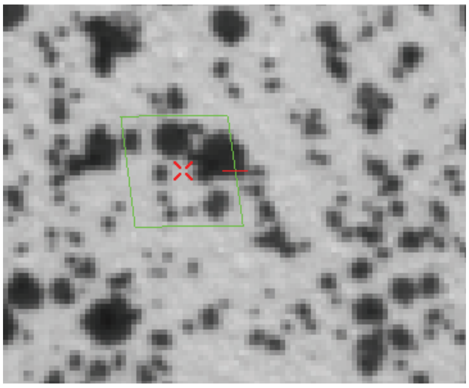

Figure 115 Startpoint Show Facet Size

SHOW MASK will display the actual mask in the image of the reference camera (lower left). Areas outside the mask are covered by a grey overlay. Using this function the user can ensure that a start point is not outside the mask in a non-valid area.

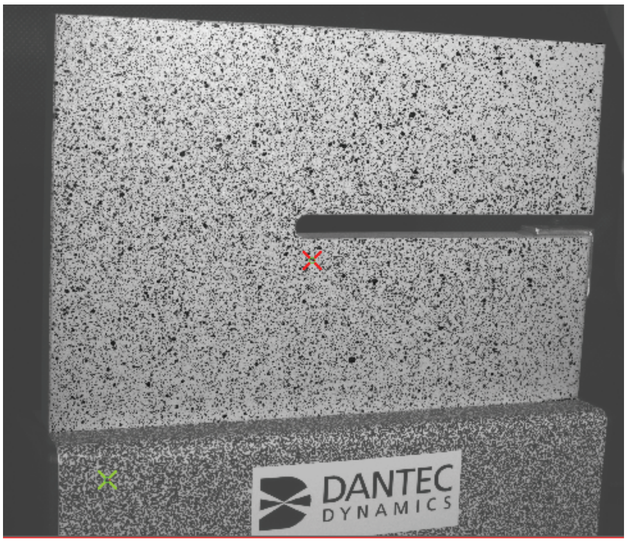

Figure 116 Image of the reference camera with mask area.

In the lower table information about each Startpoints at the active step are displayed. The following information is shown:

- *3D Residuum*: Indicating the quality of the reconstructed point in 3D. This value is used to judge the quality of found Startpoints. If the actual value is below the defined limit this point is valid otherwise the position is invalid.
- *Camera_pos_n*: Position of the Startpoint in the corresponding camera
- *Tick box*: indicating in which camera and step this Startpoint is active. As default a Startpoint is active in all cameras at all steps. A right mouse click opens a context menu with additional functions for toggling the validity of Startpoint (Figure 117)

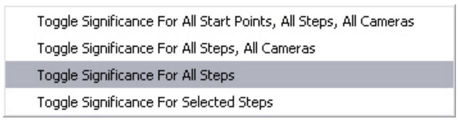

Figure 117 Startpoint Selection Menu

NOTE: *Activating or deactivating Startpoints in different cameras is important for multi-camera systems.*

Additional functions make the manual interaction with the startpoint search possible. Using **ADD** button allows the definition of new points. The additional startpoints must be placed in the reference camera in the lower left image. **DELETE** will remove the active Start-Point from all images.

Setting or correcting the position of a startpoint in the active step can be done by pressing the **SET MANUALLY** button. This function is always done in the upper images.

NOTE: *If the reference step is active the Set Manually function works only in the upper right image.*

The active Startpoint can be marked as not found in all steps by pressing the **RESET** button. In order to reset the startpoints in specified steps only mark the steps in the Steps Selection and use the right mouse (Figure 118).

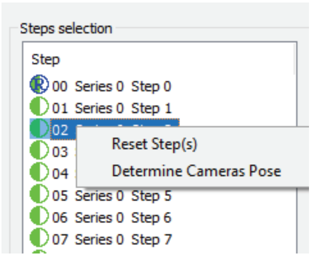

Figure 118 Startpoint search reset specified steps

The **DETERMINE CAMERA POSE** function is used for the calibration of large fields (*Calibration of large field of views*).

At the reference step it is possible to display the epipolar line for each start point. By selecting a start point for a specific camera (in *start points selection*) the corresponding epipolar is displayed in the image of that camera (Figure 119).

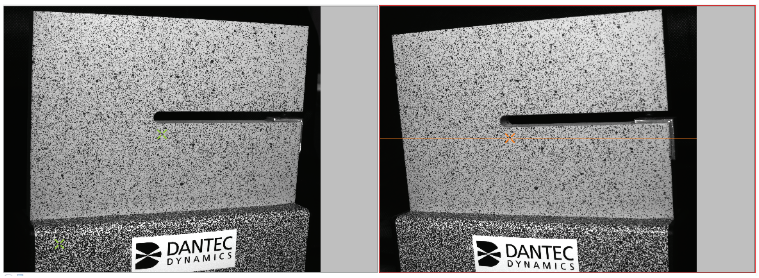

Figure 119 Startpoint search Show Epipolar Line

NOTE: *The epipolar is the line on which a point of the reference camera appears in the other camera depending on the distance of the object (for more information see also: http://en.wikipedia.org/wiki/Epipolar_geometry)*

Tip: *The epipolar might help finding the right position of the start point in the image of the other cameras.*

If the position of the startpoint is far off the line the selected projection parameter are wrong.

8. Visualization

Depending on the type of data set selected in the repository the visualization opens different display modes (Table 1:). Images are displayed in Multi Image Control Views.

8.1 Multi Image Control View

The Multi Image Control View is a control window dedicated to display images from a number of multiple images. The main application is the display of images recorded with multiple cameras.

In the lower part of the control window all available images are shown. Here the user can select the images which are displayed in the upper main part (Figure 120).

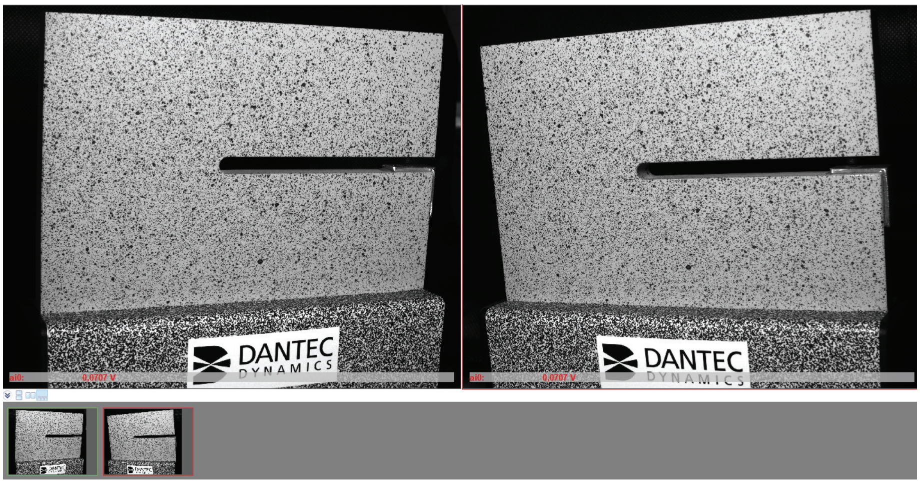

Figure 120 Control window with image list

8.1.1 Select images

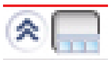

In the lower right corner control elements are located to show or hide a list of all available camera images. The first two icons open or close an additional window below the main window. Here the images of all available cameras are displayed.

To select images use the *left* mouse (in the main or the thumbnail window). For the selection of multiple images press the *Control* key and click the images in the thumbnail window.

8.1.2 Change Zoom Factor

The zoom factor for the display of the images can be set by using the control in tool bar (5.1.2). In addition the zoom factor can be adjusted by using the scroll wheel of the mouse. If the mouse is in an active window the image is zoomed in and out at the position of the mouse by rotating the scroll wheel.

If the *control key* is pressed the zoom is applied to all images. The *shift key* and a rotation of the scroll wheel puts the image back in the default display.

8.1.3 Arrangement of images

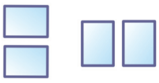

The arrangement of the images in the main window can be switched between horizontal or vertical by using the *Arrange Windows* elements in the lower right corner.

8.2 Visualization of Measured Data

If the selected entry in the repository explorer is a set of measured data, e.g. a series of images, the activation of the visualization opens a Multi Image Control View where the images from the series are shown like in a movie player. If available a time stamp when the images where acquired and the number are displayed. If analog signals are recorded with the images, the values are displayed in the lower part.

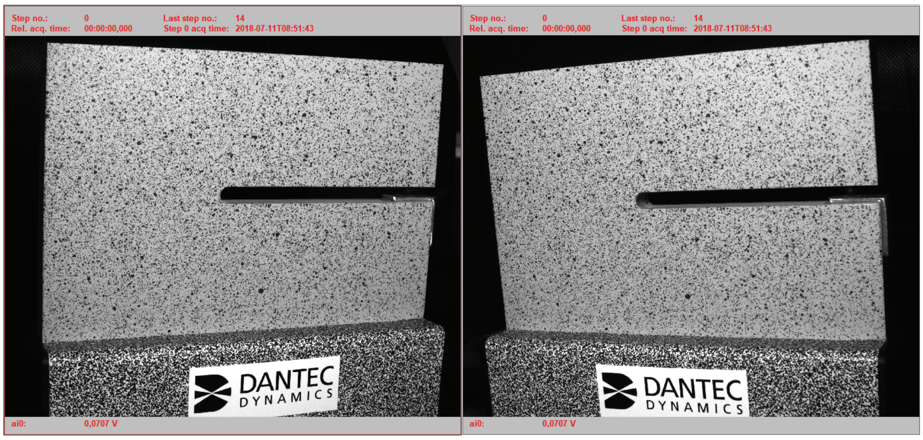

Figure 121 Visualization of acquired images

For the control of the display the *Video Control Bar* (see Table 7:) is used.

8.2.1 Display of additional information

If acquired images are displayed in the View Menu the **IN IMAGE INFO SETTINGS** function is available.

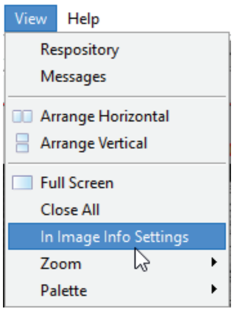

Figure 122 Menu View - In Image Info Settings

In the image view data are displayed in the top and bottom area. In the following dialog the **NAME** of area to be defined is selected (Figure 123).

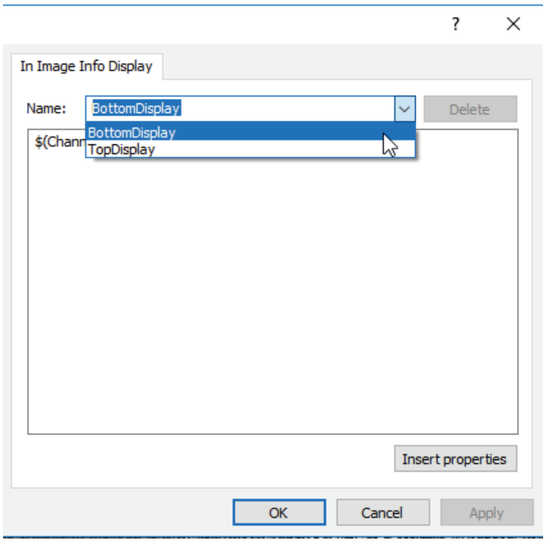

Figure 123 In Image Info Settings selection of area

In the bottom of the display, if available the analog signales recorded with the image are display by default. The user may type in any text to be displayed. With **INSERT PROPERTIES** a new window with the usable properties is open (Figure 124).

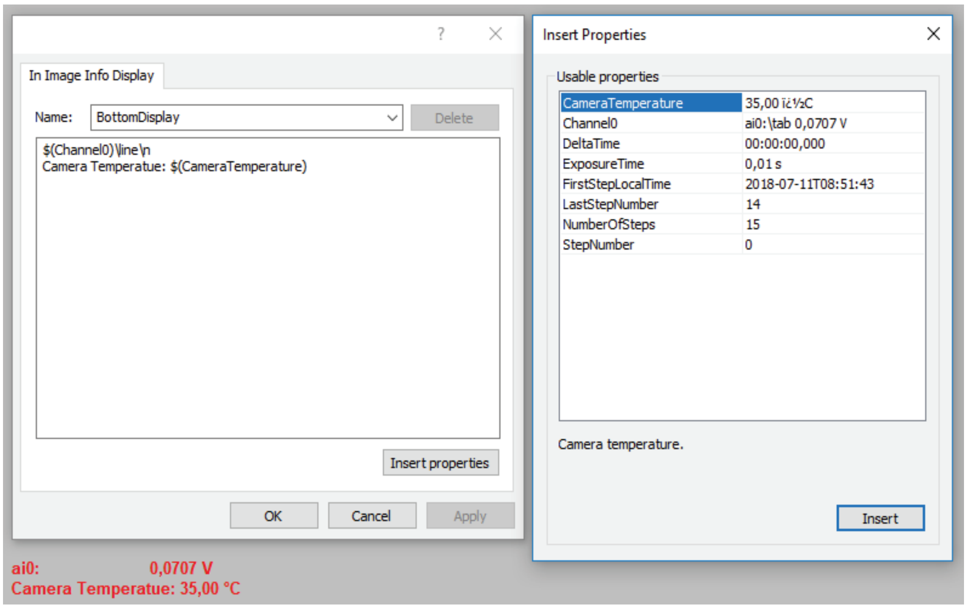

Figure 124 In Image Info Settings insert new information

If the changes are applied, the information is displayed in the bottom area with acquired images.

By default some information of the series is displayed in the top area (Figure 125). This information can be modified as well, similar to the bottom area.

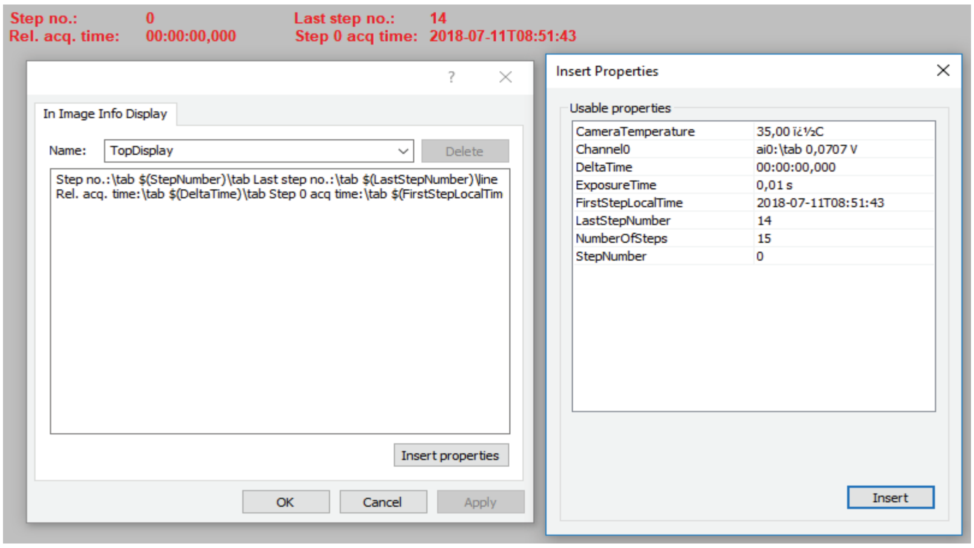

Figure 125 In Image Info Settings top area

8.3 Visualization of Evaluated Data

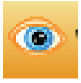

The evaluated data can be displayed and analyse within the Universal Evaluation Control in the *Visualization Settings* control (Figure 100).

In the *Visualization Settings* control the parameters of the display of the results are defined. In this visualization the results are shown as a 2D representation mapped on the live image view of the cameras. The display uses a multi camera control in order to select the camera views (Figure 128).

The features and functions of this image overlay is almost identical with the 2D representation of the *Default Visualization*. For details see Chapter 8.4.

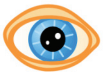

Another way of display and analyse the evaluated data, which is independent of the evaluation and can be used even it an evaluation license is not available, is the *Default Visualization*. A double click on evaluated data in the repository explorer or the Default Visualization Icon will display the data. The data can be represented as a 2D overlay (Image Overlay Display) on the camera images or as a 3D model (3D Model Display). Within the visualization the user can switch between these different views by a right mouse click on the tab of the data.

NOTE: *From a single evaluation only the Default Visualization or the Visualization Control Panel should be open at any one time*

In the following Chapter 8.4 the 2D image overlay display is described firstly in detail. The differences of the 3D model display are explained bellow in Chapter 8.5.

In the *Deafult Visualization* an additional control below the graphic window for the step selection is present (Figure 126).

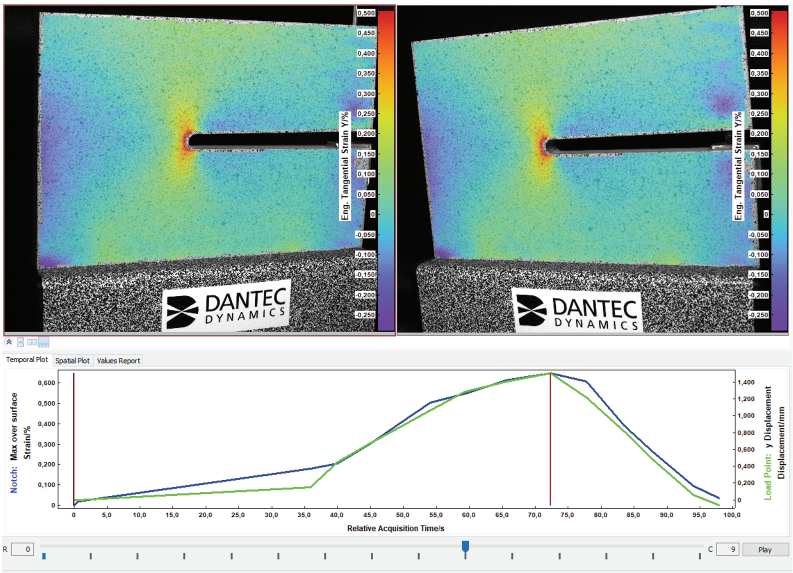

Figure 126 Default Visualization

A Right Mouse-click on the ticks set the reference step, moving the upper slider changes the current step. The number of the selected reference and current step is displayed at the end of the control (Figure 127). The **PLAY** button activates a continues change of the current step.

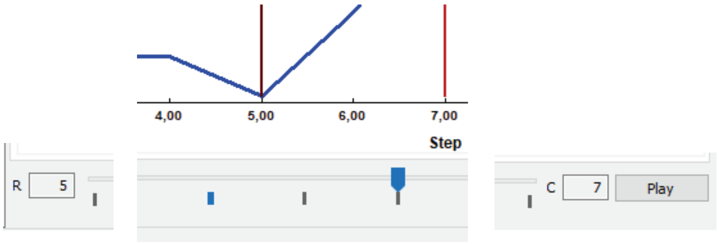

Figure 127 Default Visualization step selection control

8.4 2D Image Overlay

The upper parst display the data. In the lower area additional windows organized as tabs contain information about selected Gauge Elements, Spatial or Temporal Plots.

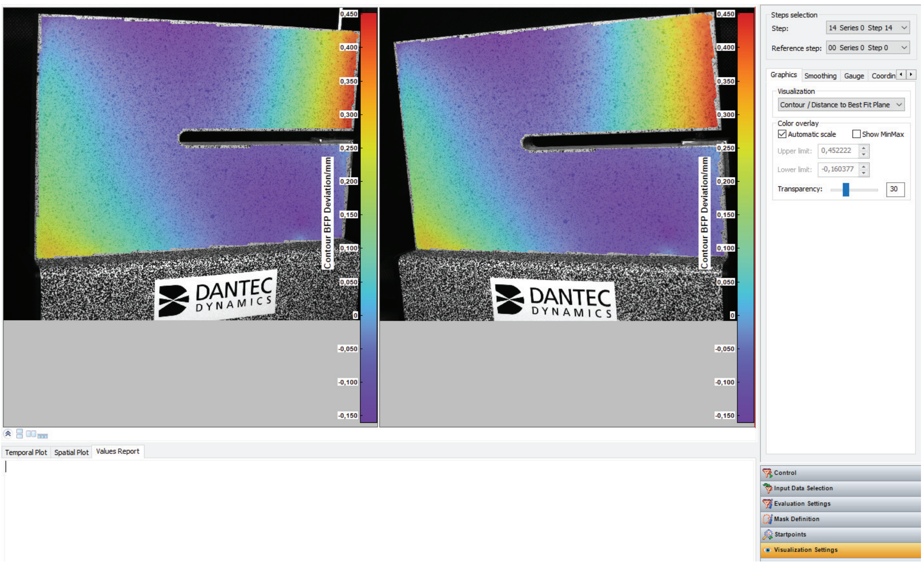

Figure 128 Visualization 2D display

A control panel on the right side contains multiple tabs for the adjustment of the data display. These tabs are described in detail int follwing chapter.

8.4.1 Graphics

The first tab selects the type of full field data, steps to be displayed and controls the scaling, transparency etc.

The *Step(s)* section defines the **REFERENCE STEP** and the actual measurement **STEP** from a list. **STEP** selects the actual step. If the measurements between two steps are compared (displacement or strain) **REFERENCE STEP** defines the unloaded step (Figure 129).

Tip: *Since the Reference and actual Step can be chosen in the visualization this gives the user greater flexibility. E.g. if the last measured step is the reference step this is like an inverted loading of the sample. Also for vibration analysis the step with the max or minimum amplitude can be selected as the reference step.*

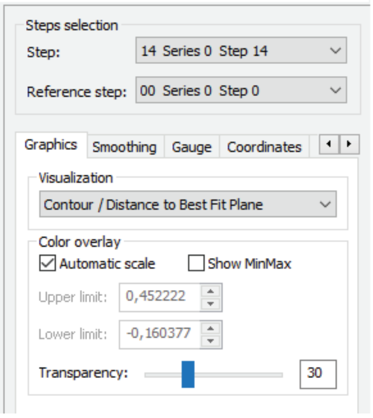

Figure 129 Visualization Graphics

The selectable data can be divided into different categories[1]:

- **OBJECT CONTOUR** represents data which is related to the contour of the actual step
- **DISPLACEMENTS** represents displacement data between the reference and the actual step
- **STRAINS** represents strain data between the reference and the actual step

The **ISTRA 4D** software contains a unit system. The user can define the unit of the displayed data of Positions, Displacements and Strains in different units. The definition of the units to be used is done in the *Help Menu*.

NOTE: *The figure above shows all possible elements. Using the Show/Hide entry in the visualization option (Show/Hide), the elements displayed can be selected.*

Object Contour

Within the Object Contour data type there is a selection of the following data sets:

- **CONTOUR ONLY** shows only the contour of the object with no mapped data set.

1. All data is in a single list

- **DISTANCE TO BEST FIT PLANE** shows the distance of the actual contour to a best fit plane mapped as a color plot on the image.
- **DISTANCE TO BEST FIT SPHERE** shows the distance of the actual contour to a best fit sphere mapped as a color plot on the image.
- **APPROXIMATED ERROR RADIUS** shows the estimated uncertainty for the 3D coordinate of each data point on the contour mapped as a color plot on the image.

Tip: *The unit of the Contour data can be changed using the settings (see also Unit System)*

Displacements

Displacements are displayed relative to a reference step, which can be set to any of the present steps. Within the *Displacements* data type there is a selection of the following data sets:

- **TOTAL DISPLACEMENT** shows the total displacement for every data point mapped as a color plot on the image[1].
- **GRID** shows the total displacement as a deformed gird on the image.
- **QUIVER** shows the total displacement as vector array mapped on the image.
- **DISPLACEMENT X** shows the displacement in X direction for every data point mapped as a color plot on the image.
- **DISPLACEMENT Y** shows the displacement in Y direction for every data point mapped as a color plot on the image.
- **DISPLACEMENT Z** shows the displacement in Z direction for every data point mapped as a color plot on the image.
- **STDDEV DISPLACEMENT X** shows the estimated standard deviation of the displacement in X direction for every data point mapped as a color plot on the image.
- **STDDEV DISPLACEMENT Y** shows the estimated standard deviation of the displacement in Y direction for every data point mapped as a color plot on the image.
- **STDDEV DISPLACEMENT Z** shows the estimated standard deviation of the displacement in Z direction for every data point mapped as a color plot on the image.

NOTE: *The results are displayed in the selected coordinate system (Coordinates)*

Tip: *The unit of the Displacement data can be changed using the settings (see also Unit System)*

1. The Total Displacement represents the length of the displacement vector

For all displacement data it's possible to calculate a rigid body movement. There are additional entries for the displacement with **REMOVE RIGID BODY MOVEMENTS** (**RBMR**). This will calculate a rigid body movement of the object between the reference and actual step and subtract this from the measured displacement.

NOTE: *The rigid body movement consist of a translation between the balance point of the two contours and a rotation which minimize the distance between the contours.*

Strain

Strain data is displayed relative to a reference step, which can be set to any of the present steps. At this time **ISTRA 4D** supports three different types of strain definitions[1]:

- Lagrange Strain (Lgr.)
- Engineering Strain (Eng.)
- True Strain (True)

Tip: *For display the type of strains can be restricted in the Options settings (Show/Hide)*

Within each of the *Strains* definitions there is a selection of the following possible data sets:

- **TANGENTIAL STRAIN X** shows the strain in X direction for every data point mapped as a color plot on the image.
- **TANGENTIAL STRAIN Y** shows the strain in Y direction for every data point mapped as a color plot on the image.
- **TANGENTIAL SHEAR STRAIN** shows the shear strain for every data point mapped as a color plot on the image.
- **PRINCIPAL STRAIN 1** shows the principal strain 1 (maximum strain) for every data point mapped as a color plot on the image.
- **PRINCIPAL STRAIN 2** shows the principal strain 2 (minimum strain) for every data point mapped as a color plot on the image.
- **STDDEV TANGENTIAL STRAIN X** shows the estimated standard deviation of the strain in X direction for every data point mapped as a color plot on the contour.
- **STDDEV TANGENTIAL STRAIN Y** shows the estimated standard deviation of the strain in Y direction for every data point mapped as a color plot on the contour.
- **STDDEV TANGENTIAL SHEAR STRAIN** shows the estimated standard deviation of the shear strain for every data point mapped as a color plot on the contour.
- **STDDEV PRINCIPAL STRAIN 1** shows the estimated standard deviation of the principal strain 1 (maximum strain) for every data point mapped as a color plot on the contour.

1. for definition of the strain see: http://en.wikipedia.org/wiki/Finite_strain_theory

- **STDDEV PRINCIPAL STRAIN 2** shows the estimated standard deviation of the principal strain 2 (minimum strain) for every data point mapped as a color plot on the contour.

NOTE: *The tangential strains are displayed in the selected coordinate system (Coordinates)*

For Multi Camera Bi-Plane Objects additional strain components are available (see 8.6.2)

Tip: *The unit of the Strain data can be changed using the settings (see also Unit System)*

Color Overlay

Most of the data are displayed as a full-field color overlay of the data onto the image of the cameras.

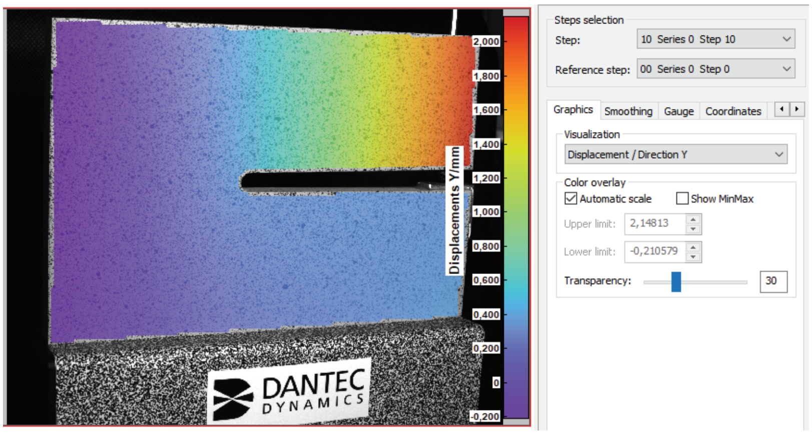

Figure 130 Visualization 2D Color Overlay

For each value map the *Color Overlay* can either be set to **AUTOMATIC SCALE** (default, color range spans minimum value to maximum value) or manually. For manual scaling, deactivate the automatic scale and set the values for the **UPPER LIMIT** and **LOWER LIMIT**.

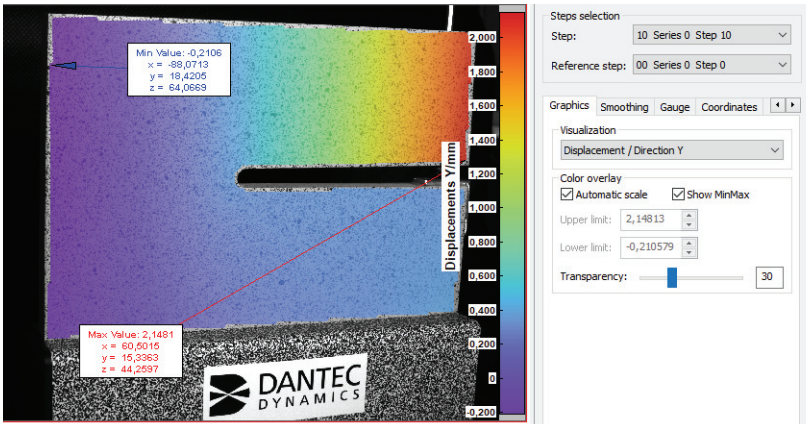

Figure 131 Visualization 2D Color Overlay with MinMax display

If **SHOW MINMAX** is active, the point of the minimum (blue) and the point of maximum (red) quantity are marked on the object. The arrow points to the loaction of the corresponding value. In addition to the value the coordinates of the position in the active coordinate system are displayed.

Tip: *The position of the labels can be changed by click in the lable and drag the lable to another position. The position is fixed for all steps.*

The **TRANSPARENCY** slider defines the ratio between the display of the evaluated data and the image of the camera. In the left position (*transparency = 0*) only the evaluated data are shown and no image is of the camera is visible. In the most right position (*100*) only the image of the camera without any data overlaid is shown.

Grid or Vector Display

The color overlay can display a single value only. For a better display of the 3-dimensional displacements two additional representations of the 3D displacement data projected onto the camera image are available.

In the grid display, a green grid shows the contour at the refrence step. A red grid display the deformed grid at the actual step (Figure 132).

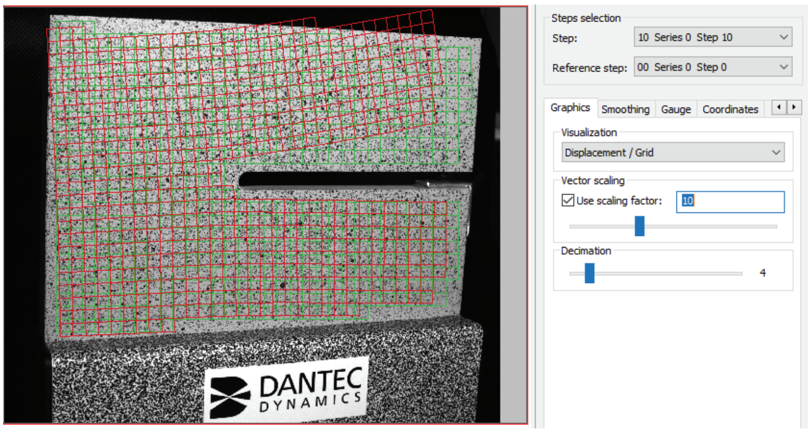

Figure 132 Visualization 2D Grid Display

For proper visualization of very small or pretty large displacements, the grid of the actual step can be modified in the *Vector Scaling* section. A **SCALING FACTOR** scales the displacements to an arbitrary factor. The slider sets the scaling factor in a range of 1 to 1000. The user may type any positive number in the field as well.

The density of the grid is controlled by the **DECIMATION** setting. At a decimation of 1 every data point is used and a square resents a single data point. At a decimation of 5 every 5th data point in horizontal and vertical direction is used and a square represents 25 data points.

The Quiver display represents the 3D displacement as a vector field projected onto the camera image (Figure 133). The total displacement is the length of the vectors and it is represented by the color of the vector.

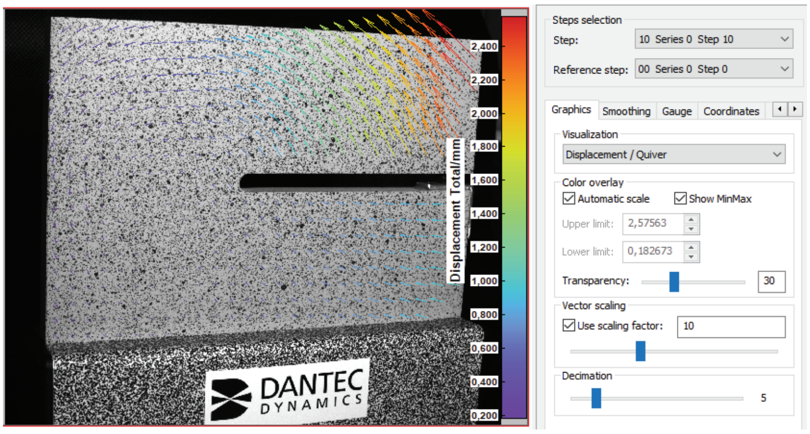

Figure 133 Visualization 2D Quiver Display

Tip: *For the display of the Grid and Quiver the image of the reference step or the image of the acutal step can be used. The user can swicht between both methods in the Option-Visualization settings (Visualization)*

8.4.2 Smoothing

The *Smoothing* tab defines the method and strength of smoothing of the data. The data is smoothed online, whenever a new data display is activated (Figure 134).

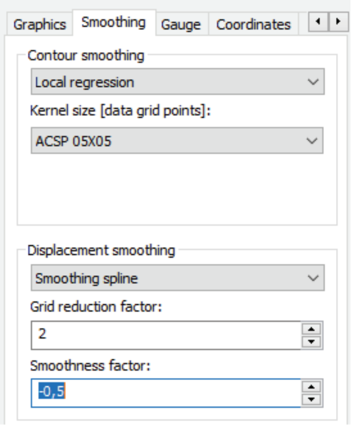

Figure 134 Visualization Smoothing

The settings for smoothing of the contour and displacement are independent. The **LOCAL REGRESSION** filter is based on an adaptive spline polynomial algorithm (ACSP). The strength of the smoothing is defined by the kernel size. This filter is optimized for localized effects as only neighbor points are used. The **SMOOTHING SPLINE** filter uses spline functions to represent the complete data

set. The strength can be controlled by two parameters. The **GRID REDUCTION** factor defines the density of the grid points relative to the data gird. The higher the reduction factor the less grid points and the smoother the data is. As a second constraint the **SMOOTHNESS FACTOR** limits the global curvature under the spline function. The higher this factor the more smoothed the data is. This filter smooths data globally.

NOTE: *Both types of filter can be used for the contour and displacement.*

The strain can be calculated by the distortion of the facets or by calculating the gradient of the deformation. If no filtering is used the strain will be calculated only from the distortion of the facet. An increased filter will increase the influence of the deformation gradient for the calculation of the strain.

An increased filter generates more smoothed results and decreases the StdDev of the results. But also decreases the spatial resolution.

Tip: *As the performance of the Smoothing spline filter is independent of the strength it is recommended to use this for the strain calculation. The contour might be filtered by a Local regression filter with a kernel size between 5x5 to 9x9. The settings of the spline filter for the displacement might be grid reduction of 2-3 and a smoothness factor of -1.0 to 0.0.*

8.4.3 Gauge

The *Gauge Visualization* is an expanded tool for analysis of the evaluated data. It provides the possibility for defining points (*New Point*), lines (*New Line*) and areas (*New Circle*, *New Polygon*) on the object surface and to evaluate and visualize contour, displacement and strain data on these objects (Figure 135).

If available the relative Time of the acquisition and Analog Input data are displayed as *Time* and *Analog Channels* in the Gauge tree.The item *Channels Sets* is used if external data are integrated (see *Samples of Recording Procedures*).

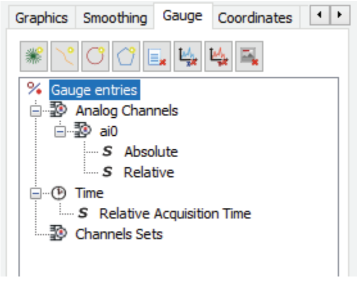

Figure 135 Visualization Gauge

The elements in the *Gauge Visualization* are arranged as nodes in a tree, each node allowing access to different types of data and data evaluation methods. Open a node by clicking the “+”-sign in For the *Time* element the *Relative Acquisition Time* is defined as

the time difference to the selected reference step. For each Analog Channel the absolute value and the relative value to the selected reference step is available.

NOTE: *An element is activated by a highlighting in the gauge tree or a click on the element in the graphic display. The position of an element can be changed by pressing the contrl-key, a click on the element in the graphic display and dragging it to the new position.*

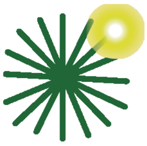

New Point

To create a new gauge point press the *New Point* button in the control panel and click on the image with the left mouse button for positioning the point (Figure 136). It doesn‘t matter which image is used to position the point it will be shown in every displayed image. In the gauge tree a point element is added.

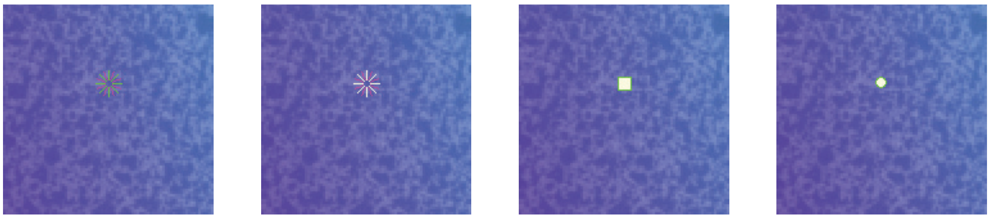

Figure 136 Visualization Gauge New Point

The style of the point element can be defined in the corresponding options entry (*Gauge Style*). In the upper Figure 136 the display in the *Default*, *Star*, *Square* and *Cyle* Style is shown. The color and size can be changed as well.

New Line

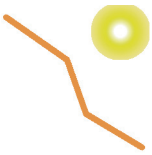

A line element on the object surface is defined by at least two points. The user may also add multiple intermediate points and create a poly line. To create a new line point press the *New Line* button in the control panel and click on the image. Use the left

mouse button for positioning of additional points of the line. The right mouse defines the end point of the line (Figure 137). In the gauge tree a line element is added.

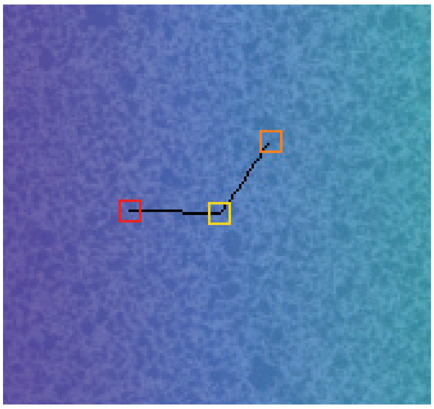

Figure 137 Visualization Gauge New Line

The start point is displayed in red, intermediate points in yellow and the end point in an orange color.

NOTE: *For a two point line element additional extensometer functions are available.*

Tip: *It is possible to start the definition of a line in one image and continue the line in another image.*

The style and color of the markers and the line can be changed in the corresponding options entry (Gauge Style).

New Circle

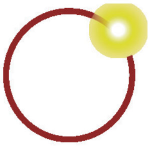

A circle is an area element on the object surface defined by two points defining the diameter of the circle line. To create a new circle point press the *New Circle* button in the control panel and click on the image. Use the left mouse button for positioning the points determining the diameter of the circle.

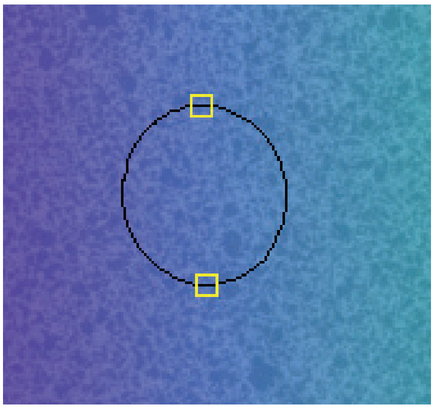

Figure 138 Visualization Gauge New Circle

At the selected positions markers (color: yellow) are shown and the surrounding circle line is drawn. In the gauge tree a circle element is added.

Tip: *The style and color of the markers and the line can be changed in the corresponding options entry (Gauge Style).*

New Polygon

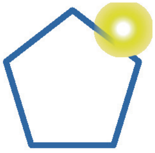

A polygon is an area element on the object surface defined by a surrounding poly line. To create a new polygon point press the *New Polygon* button in the control panel and click on the image. Use the left mouse button for positioning points along the border. The right mouse defines the last point and closes the border.

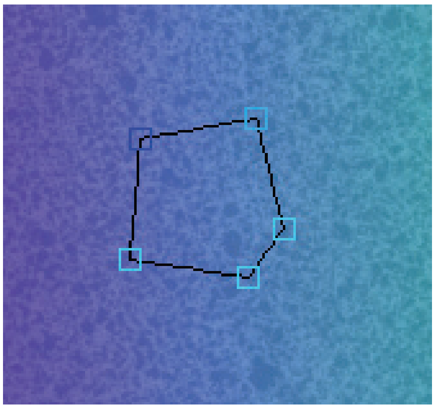

Figure 139 Visualization Gauge New Polygon

The start/end points are displayed in dark blue, the intermediate points in a light blue color. The style and color of the markers and the line can be changed in the corresponding options entry (*Gauge Style*).

Tip: *It is possible to start the definition of a polygon in one image and continue the line in another image.*

The style and color of the markers and the line can be changed in the corresponding options entry (Gauge Style).

Gauge Entries Tree

For the Point, Line and Area Elements the data is divided into different categories *Contour*, *Displacements* and *Strains*. Open any of these groups to get to the corresponding values.

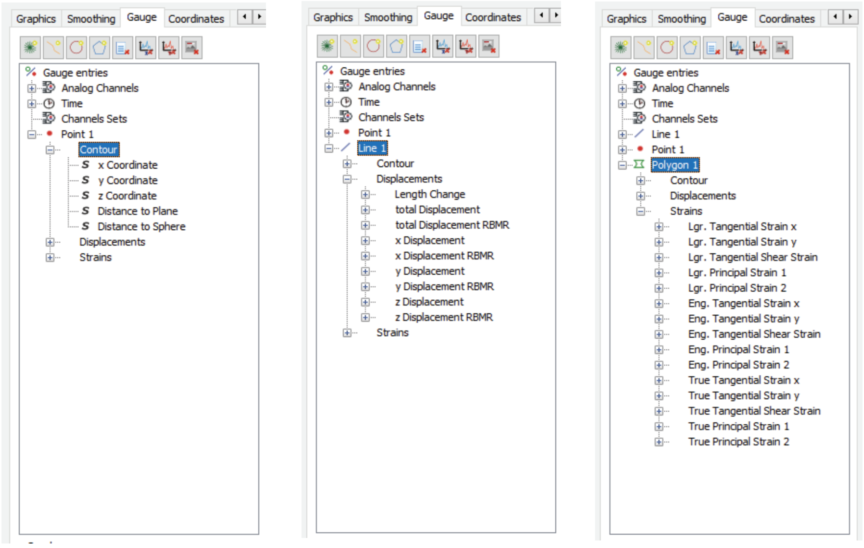

Figure 140 Visualization Gauge Point, Line Polygon Element

Within the displacements category the values are available with or without *Rigid Body Movement ReMoved* (*RBMRM*).

Move the mouse over any of the nodes in order to get the data shown as a tool tip display. Whenever the actual data is changed, e.g. if a different step is selected or smoothing settings changed, the gauge values are updated automatically.

NOTE: *All data are displayed with the value and an estimated standard deviation*

Depending on the dimension of the element additional information is available:

- *1D (Line)* **MEAN**, **MAX**, **MIN** and **STDDEV** of the data
- *2D (Polygon, Circle)* **MEAN**, **MAX** and **MIN** each **OVER THE BORDER** or **SURFACE** of the element

For a two point line element an additional **EXTENSOMETER FUNCTION** is available. In the contour node a **GAUGE LENGTH** entry is available (Figure 141). The values showing the absolute distance

between the start and end points at the selected step. In the Displacements node the change of length between the reference and actual step is available.

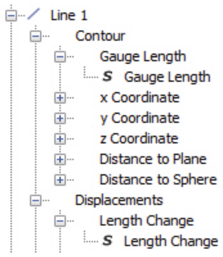

Figure 141 Visualization Gauge Extensometer

To conform to standards the length of the two point line can be defined at the actual step. A right mouse click on one of the points allows the adjustment of the length of the line(Figure 142).

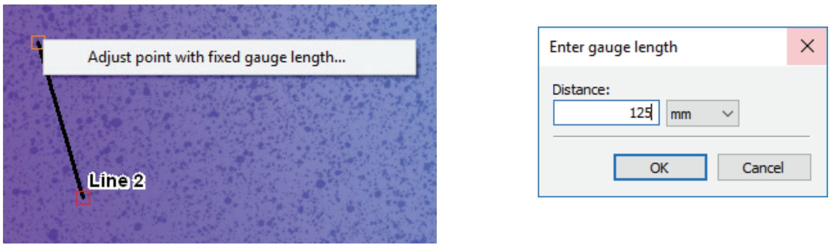

Figure 142 Visualization Gauge Set Extensometer Length

The position of the selected point is changed along the direction of the line until the distance between the start and end point complies with the defined gauge length.

Gauge Data Labels

If a new gauge element is created a default name is given to the element. The name is compiled from the type and a sequential numbering. The user can change the name of each element by using a right click the rename option is available (Figure 143).

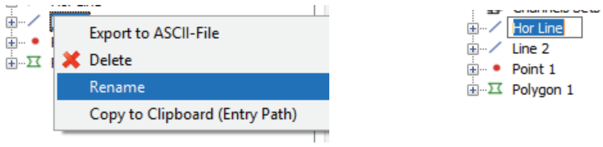

Figure 143 Visualization Gauge Rename Elements

By activating the Overlay Labels in the Gauge panel or Gauge Style options, the names of the gauge elements can be plotted as well with the elements (Figure 144). The style of the labels can be

defined in the Gauge Style option (*Gauge Style*). The font and color of the text can be selected. Optional a background box can be selected. The type, color and size can be changed as well.

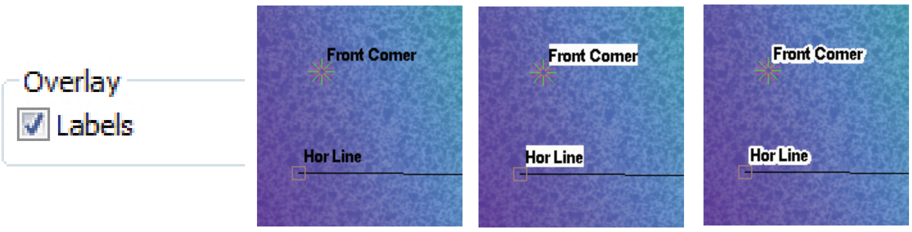

Figure 144 Visualization of Gauge Elements with labels

NOTE: *This option is available in 2D overlay only.*

Display Gauge Data

There are three different ways for each element to show within the software or export the information via the clipboard. The information is updated whenever the actual settings are changed.

- **REPORT ENTRY**: Display the value of the selected information at the actual step. This information can be displayed in the Report Tab.

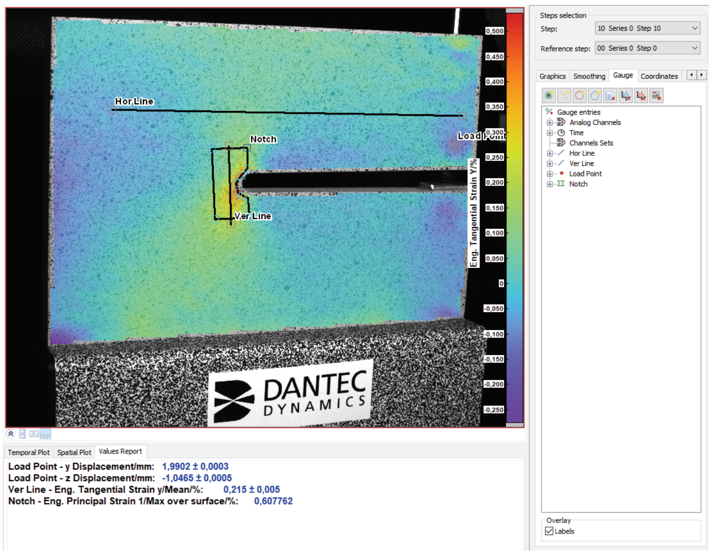

Figure 145 Visualization Gauge Report Entry

Tip: *A right mouse click in the Value Report window allows the change of the font size*

The information can be added into the 2D overlay display by selecting the corrsponding function Show in Visualization (see Table 14:)

- **SPATIAL**: Display the values of the selected information along the line of the actual step. Available for line information. This information can be plotted in the Spatial Tab.

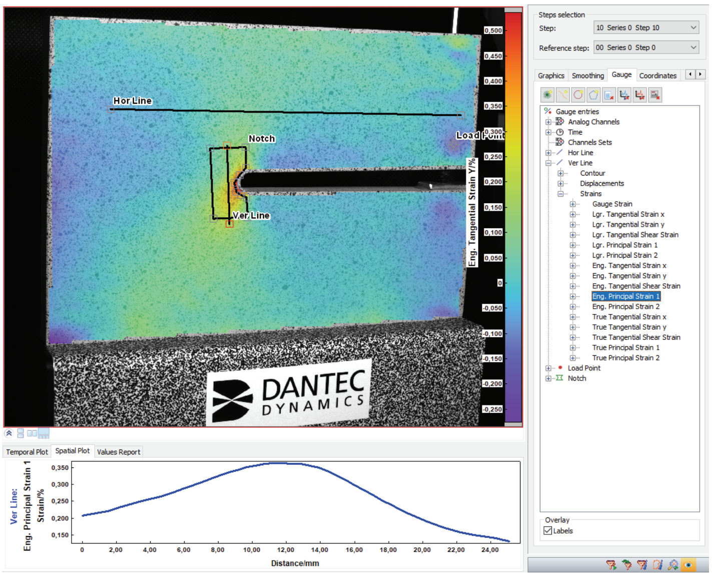

Figure 146 Visualization Gauge Spatial Plot

- **TEMPORAL**: Display the values of the selected information over all steps of the actual evaluation. Available for point information. This information can be plotted in the Temporal Tab.

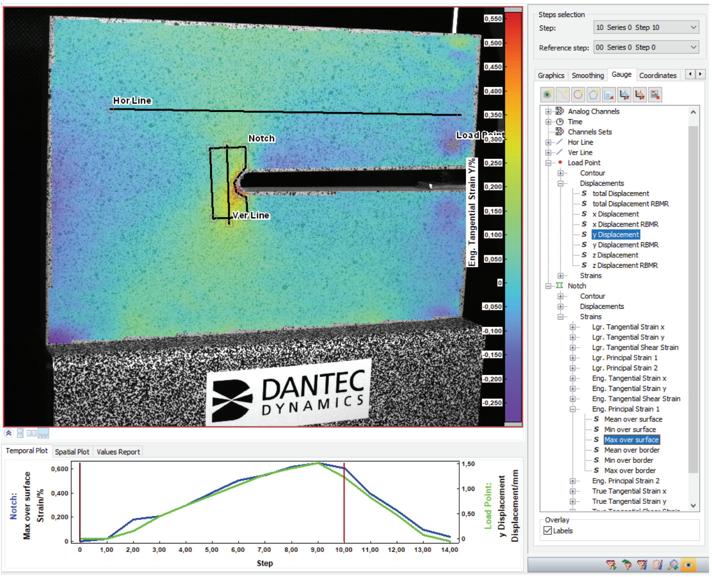

Figure 147 Visualization Gauge Temporal Plot

The two vertical lines indicating the position of the reference step and the current step in the measurement series. The color of the lines can be defined in the Gauge Style options (*Gauge Style*).

By default the temporal data are plotted over the measurement steps. If information about the acquisition time of the measurement steps is available a right mouse click on the x-axis of the temporal plot allows switching between the *plot over step* number or *over time* (Figure 148).

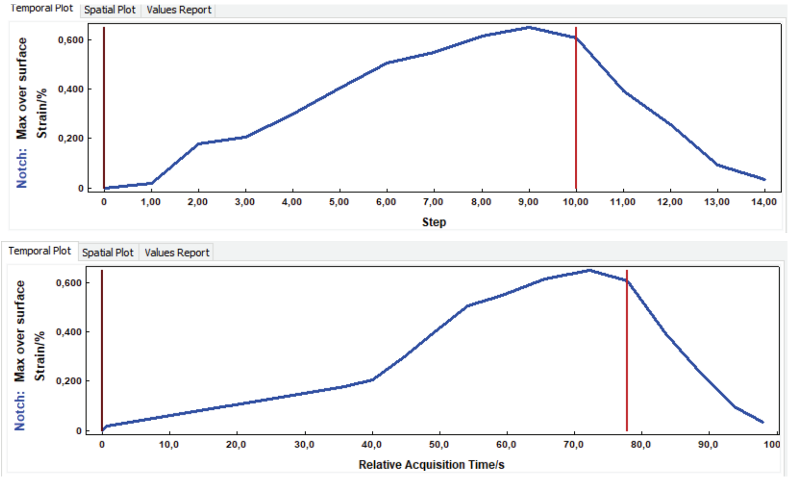

Figure 148 Temporal Plot over step number or time

The temporal plot can display up to two lines of information. The first uses the left Y-axis, the second the right Y-axis. A second information can be added by right mouse click on a corresponding element in the gauge tree (Figure 150).

The first line is displayed in blue, the second in green color. Both lines can use different units and the axes are scaled independently.

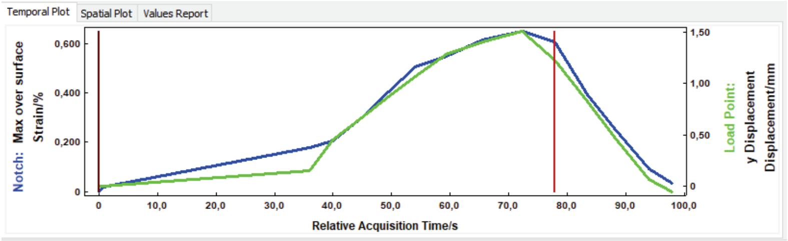

Figure 149 Temporal Plot two lines

The scale of the axis can be changed by pressing the *Ctrl key* and dragging the axis with a *mouse click*. Is the mouse located at the lower half of the axis the minimum value is changed; on the upper half the maximum is changed.

NOTE: *It is only possible to zoom into the data (increase the minimum or decrease the maximum) but not zoom out.*

A click on the *Gauge Entries* or *Channels Sets* shows the option to **INTEGRATE CHANNEL SETS** to the existing evaluated data. This feature allows the integration of external data to the existing measurement. The source of this additional data is an *ASCII* file. It can be used to add analog values to an evaluation of imported

images or to add data from an external device where no analog voltage information is available. A detailed description of the use of this feature can be found at Chapter 12.3.

A right mouse click on the elements in the gauge tree opens a menu with various possible methods to display the data in a report, display in a plot or export the information via the clipboard. The methods depends on the type and dimension of the selected data.

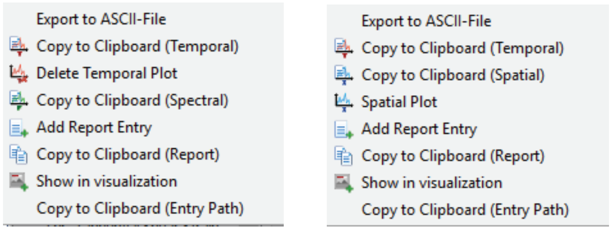

Figure 150 Visualization Gauge Export Context Menu

The following table lists all available options:

Copy the value of the data to the *Report* window	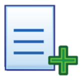
Copy the value of the data to the Clipboard	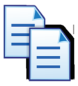
Delete the content from the *Report* window	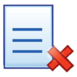
Plot the data of the element from the active step to the *Spatial Plot* window	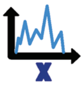
Copy the data of the element from the active step to the Clipboard. The information includes a heading and three columns (Position, Data, StdDev)	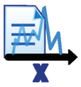
Delete the content from the *Spatial Plot* window	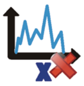

Table 14: Gauge Data Export

Plot the data over the loading step in the *Temporal Plot* window	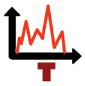
Copy the data over the loading step in the Clipboard. The information includes a heading and three columns (Step Number, Data, StdDev)	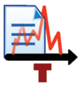
Delete the content from the *Temporal Plot* window	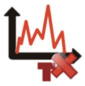
Copy the the spectral information (FFT over time) of the data in the clipboard[a]	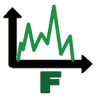
Overlay the value of the data into the *2D Visualiztion* window	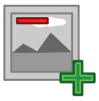

Table 14: Gauge Data Export

a. The Spectral Information is an optional feature and requires the corresponding licence option.

Tip: *If the additional plot windows are not visible, they might be hidden by the main graphic window. In this case you can go with the mouse to the lower border of the main graphic window and move the border up until the plot windows are visible*

NOTE: *If the information of the frequency of acquisition is not available, the user needs to enter the time between two images in the respective dialog.*

ASCII Export

The option Export to ASCII-File saves the selected information directly in a text file on the hard drive. Here multiple information can be marked and exported in a single file.

If the *ASCII Export* is selected a wizard guides the user through the options. The first step is the definition on the file name and storage directory (Figure 151).

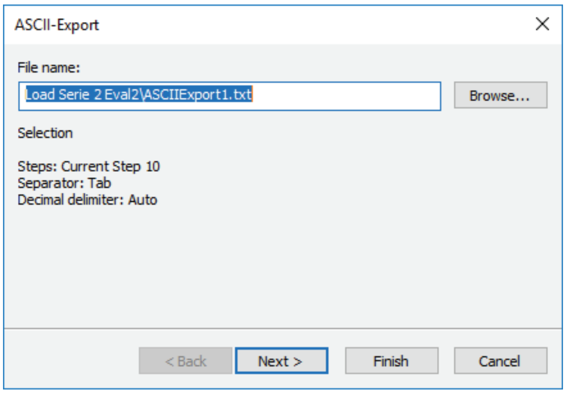

Figure 151 Gauge ASCII Export Wizard File Name

The information in *Selection* shows the actual settings for this export. If the user agrees with these they can start the export by clicking the **FINISH** button directly.

The next page defines the steps to be exported (Figure 152). **CURRENT** exports only the active step, **ALL** uses all available steps and using **STEPS FROM** defines a section of the series.

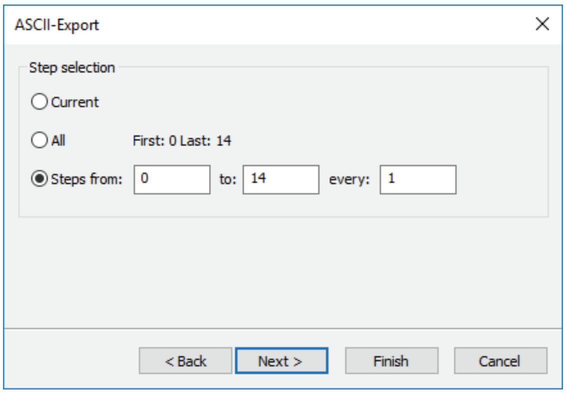

Figure 152 Gauge ASCII Export Wizard Step Selection

Finally the *Separator* between the data in a row and the *Decimal delimiter* for the numbers are defined (Figure 153).

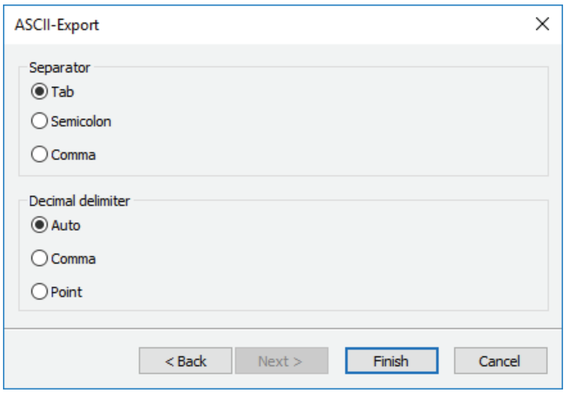

Figure 153 Gauge ASCII Export Wizard Separator

As the *Separator* the choice is between a **TAB**, **SEMICOLON** or **COMMA**. The *Decimal delimiter* can be selected by the actual settings of the operating system by using the **AUTO** selection or defined by the user explicitly as **COMMA** or **POINT**.

8.4.4 Coordinates

The data display is heavily influenced by the underlying coordinate system. A change of the coordinate system will cause a change of e.g. *X*, *Y* and *Z Contour, Displacement X*, Y and Z as well as the *Tangential Strain X*, *Y* and *Tangential Shear Strain*.

In the *Coordinates* tab the *Coordinate System* list appears. There exist a predefined coordinate systems, the **CALIBRATION SYSTEM**, the **CAMERA_POS SYSTEMS** and the **SENSOR SYSTEM**, which were generated during the data evaluation process.

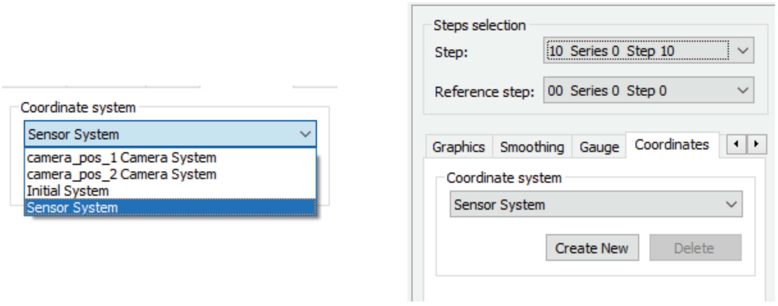

Figure 154 Visualization Coordinate Systems

NOTE: *It's possible to define multiple coordinate systems within one data set and select the actual one to use from the Coordinate System list.*

The **CALIBRATION SYSTEM** is the default coordinate system, defined by the three dimensional coordinates of the calibration target position in the first exposure of the calibration procedure (*Calibration Process*). The coordinate origin is set to the centre of the calibration target, defined by the centre of the circle in the

central square. The z-direction points to the direction normal to the calibration target towards the sensor. The x-direction is in the plane of the calibration target and points from the central circle to the circle in the neighboring square.

The **CAMERA_POS SYSTEMS** exist for each camera. The X-Y plane is in the image plane of the camera. The z-direction is in viewing direction to the object.

The **SENSOR SYSTEM** is bound to the sensor in a way that the coordinate's origin is set to the centre point of the connecting line of the two sensor cameras. The z-direction points towards the line of site of the two cameras and is defined by the mean direction of the corresponding optical axes. The x-direction is along the connecting line of the two sensor cameras, pointing from the coordinates origin towards camera 2.

NOTE: *In case of a Multi Camera Evaluation the Sensor System is replaced by an Object System. In this case the axis of the coordinate system is defined according the selected object type*

In addition a user can create their own coordinate systems.

Create new coordinate systems

Beside the default coordinate systems the user can define their own coordinate system. By clicking on the *Create New* button a dialog comes up. The name which is used to refer to this coordinate system is given in the *New Coordinate System Name*.

The method which is used is selected in the *Definition Method* section (Figure 155).

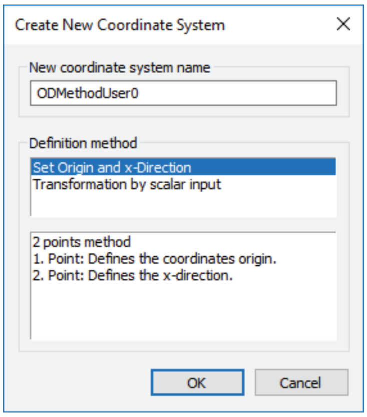

Figure 155 Dialog Create New Coordinate System

The **TWO POINT METHOD** is based on the definition of the origin and the X direction on the object surface (Figure 156). This method creates a coordinate system, which is bound to the specimen. It offers two markers, which are set to the specimen surface. Both markers can be moved over the surface by dragging with the

Mouse. The red marker sets the coordinates origin, the blue marker defines the x-direction as the connecting line between the red and light blue marker.

NOTE: *A marker can be moved by pressing the contrl-key and a left mouse click on the marker.*

Tip: *In order to position the markers at defined positions on the object it might be useful to put some marker on the surface. These can be seen in the images and be used to place the marker at the required position.*

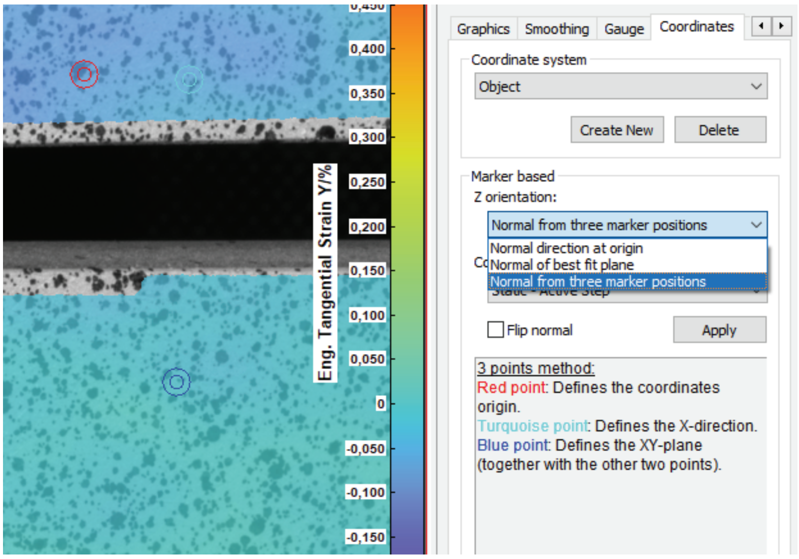

Figure 156 Visualization control panel Coordinates 2 Points Method

The z direction can be defined as **NORMAL DIRECTION AT ORIGIN**, **NORMAL OF BEST FIT PLANE** or **NORMAL FROM THREE MARKER POSITIONS**. In case of **NORMAL DIRECTION AT ORIGIN** the normal vector of the contour determined from the area around the origin (red marker) is used to define z direction. **NORMAL OF BEST FIT PLANE** does use the complete reconstructed contour to calculate a best fit plane through the contour and uses the normal vector of this plane as the z direction. In the third method **NORMAL FROM THREE MARKER POSITIONS** the xy plane is defined by the position of the origin, x direction and a third marker (dark blue). In this case the position of these points is used to define the xy plane. The normal vector of this xy plane is then the z direction.

In general the z direction is defined from the object to the sensor. If **FLIP NORMAL** is selected, the z direction is pointing in the oposite direction, from the sensor to the object.

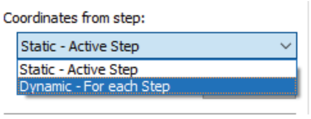

Figure 157 Visualization control panel Coordinates Step Selection

As another option the user can select the coordinate system as **STATIC**, where it is defined at the active step and it is fixed in space for all other steps even if the object is moving. In the **DYNAMIC** case the coordinate system sticks to the object and moves with the object. Here the coordinate system might be different for each step.

The coordinate system is activated and saved permanently to the data by clicking the **APPLY** button. It is added to the list of available coordinate systems for this data set. Whenever the coordinate system is reactivated by selecting the corresponding entry of the list, the markers appear and the coordinate system can be changed. Whenever the **APPLY** button is pressed, the previous coordinates settings are overwritten by the new ones defined by the actual marker positions.

Tip: *In the case that the user is interested in the relative movement of different parts within the field of evaluation the coordinate system should be dynamic and defined by three points.*

With the **TRANSFORMATION BY SCALAR INPUT** method the transformation is defined as rotation and translation with respect to the default coordinate system.

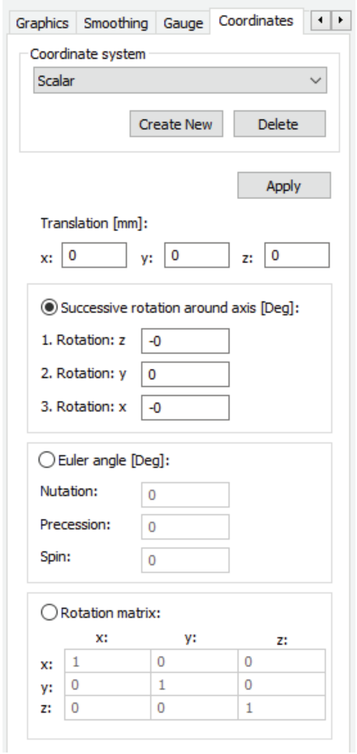

Figure 158 Visualization control panel Coordinates Scalar Input

The translation vector can be entered in the first section. The unit for the **TRANSLATION** is mm and given for each component.

For the rotation different methods are implemented:

- A **SUCCESSIVE ROTATION AROUND AXIS**, here the amount of rotation around the axis is given in Deg. The sequence of rotation is: Z-, Y- and X-Axis.
- Using the **EULER ANGLE** the amount of rotation is given in Deg in the Euler notation. The sequence of the rotation is: Z-, Y- and Z-Axis.
- In the **ROTATION MATRIX** the individual elements of the rotation matrix are defined.

NOTE: *The transformation is entered from the target coordinate system back to the default calibration coordinate system.*

8.4.5 Stencil

The *Stencil* panel is only available in the 2D Overlay of the *Default Visualization*, it is not available in the *Visualization Settings* in the Universal Evaluation Control.

The stencil is defining a visualization mask. With the use of the stencil function, evaluated points can be excluded from the visualization without re-running the evaluation of the data.

In the stencil mode the evaluated data points are shown as crosses in the overlay on the object (Figure 159).

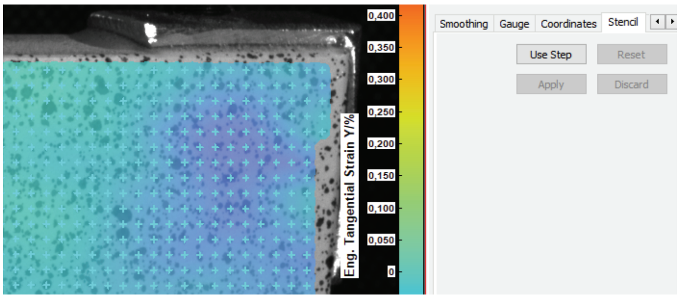

Figure 159 Stencil mode with display of data points

USE STEP will select the actual step for defining visualization mask. An area of points is selceted by moving the left clicked mouse with pressing ctrl key. Individual points can be selcted or unselected by klicking with the right mouse button on the points (Figure 160).

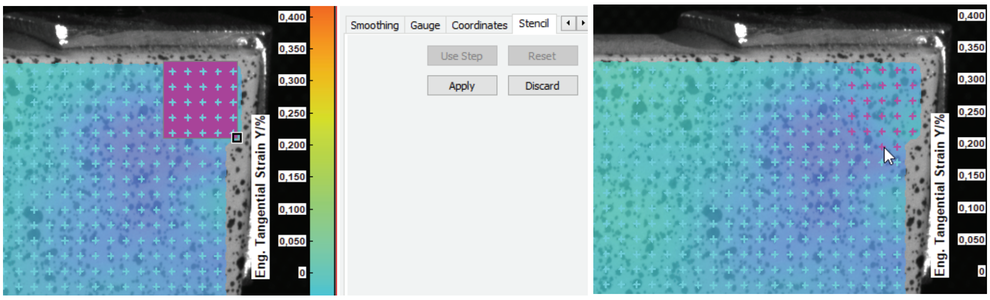

Figure 160 Stencil mode marking points (left area, right individual)

The selection can be **DISCARD** or by using **APPLY**, they will be masked out in the normal graphics display (Figure 161). **RESET** will show all data points again.

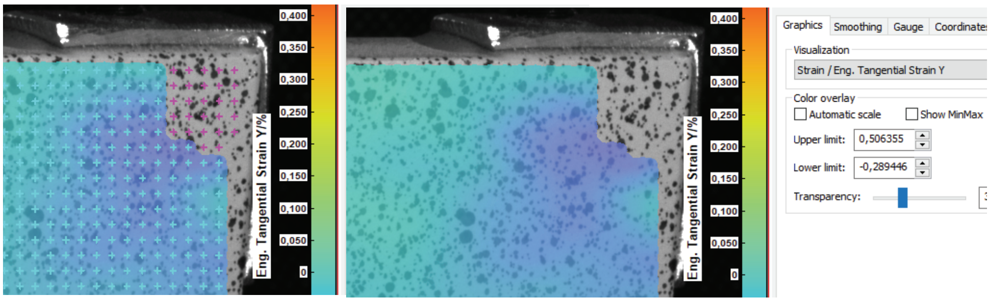

Figure 161 Stencil mode marked points (left stencil mode, right graphics mode)

NOTE: *Only the for the actual step calculated data points are displayed and only these can be marked. In order to see all data points the reference step needs to be selected.*

8.4.6 Modules

The **MODULES** are additional features for the post processing of the evaluated data into special applications. The *Working Directory* points to the directory containing the available modules. The *Job Definitions* list contains all available modules in the working directory and selects one module from the list. The *Refresh* button will update the list in case modifications were made. The **START** button activates the selected module.

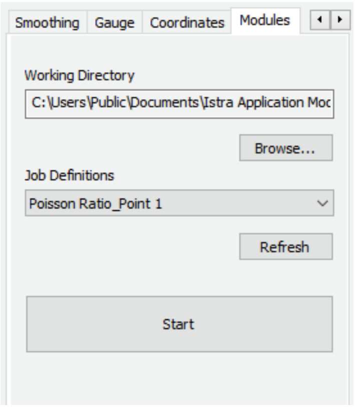

Figure 162 Visualization control panel Modules

A more detailed description of the Modules can be found on the *Dantec Dynamics e-Learning Portal*.

NOTE: *For more details on the Dantec Dynamics e-Learning Portal and how to access this portal, please contact your local sales representative.*

8.5 3D Model Visualization

In addition to the 2D Overlay Display the evaluated data can be displayed as 3D Model Display. This visualization uses the calculated 3D contour to build a 3D model and maps the results on to this contour (Figure 163). In this type of visualization only evaluated areas can be displayed.

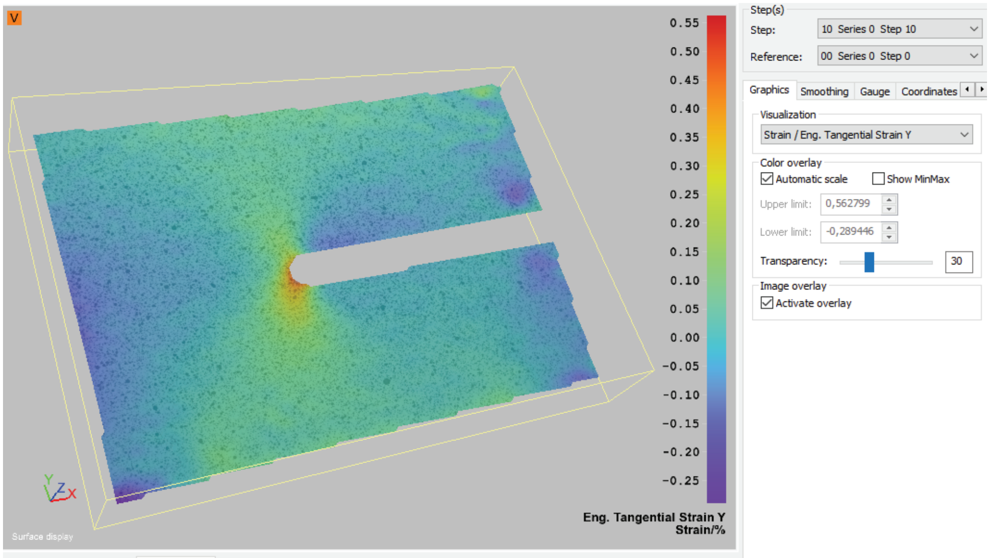

Figure 163 3D Visualization Contour and Distance to Bestfit Plane

On the left side the 3D Graphic of the measured object is shown, on the right side the settings and control elements are grouped in the control panel. These panels are identical to the 2D Visualization (see *2D Image Overlay*).

The orientation of the 3D object can be changed with the mouse. The following functions are possible by pressing:

- **LEFT MOUSE**: Moving the mouse rotates the object
- **RIGHT MOUSE**: Moving up and down zooms out or zooms in
- **LEFT + RIGHT MOUSE**: Moves the object

The standard view is the rendered object surface. The **SPACE BAR** brings the view back to the default position. The box indicates the coordinate system. In the default view the X-Axis is in horizontal direction, the Y-Axis in vertical and the Z-Axis is pointing perpendicular to the XY-Plane. In addition the orientation of the coordinate system is indicated in the lower left corner.

In the *Display* section the user has the choice to select the display of the **3D GRAPHICS** only or to display the **GRAPHICS AND REPORTS**, here in the lower part a report window, a spatial or temporal line plot is added to the display.

The background color of the window is defined in the Visualization section of the Istra4D Options (*Visualization*).

In the 3D Visualization the color scaling can be changed by using the control elements in the *color overlay* section (see *2D Image Overlay*) or by using the mouse on the color scale directly. Using a left mouse click on the color scale, the selected value can be moved up and down. If the mouse is in the upper half of the color scale bar the upper limit is changed. If the mouse is in the lower part of the bar the lower limit is changed.

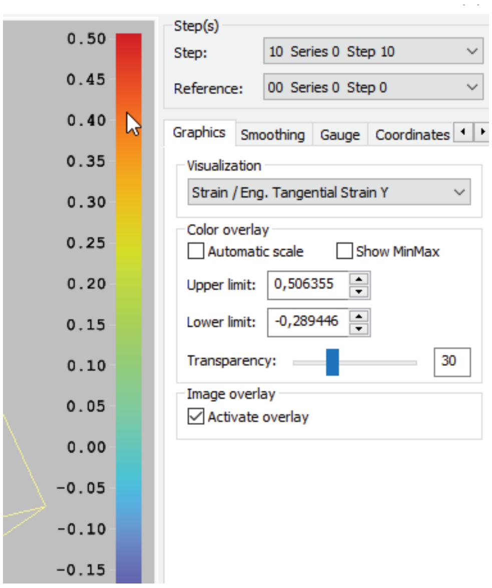

Figure 164 3D Visualization Change color scaling by mouse

8.5.1 Orthographic projections

In addition to the standard 3D Visualization, with the possibility of rotating the 3D model, orthographic projections can be selected. The selection element **V** in the upper left corner of the 3D Display toggles the display of additional buttons controlling the display of the orthographic projections.

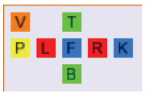

Toggle display of Orthographic Projection Control Elements	**V**
Select 3D Display mode	**P**
Select Projection onto XY Plane (**F**ront Bac**K**)	**F** **K**

Table 15: Orthographic Projections Control Elements

Select Projection onto XZ Plane (**T**op **B**ottom)	**T** **B**
Select Projection onto YZ Plane (**L**eft **R**ight)	**L** **R**

Table 15: Orthographic Projections Control Elements

In the orthographic projections the 3D object is projected onto one of the planes defined by two axis of the coordinate system. An alignment grid is underplayed to the projected object (Figure 165). The distance of the grid lines is determined by the overall dimension of the object.

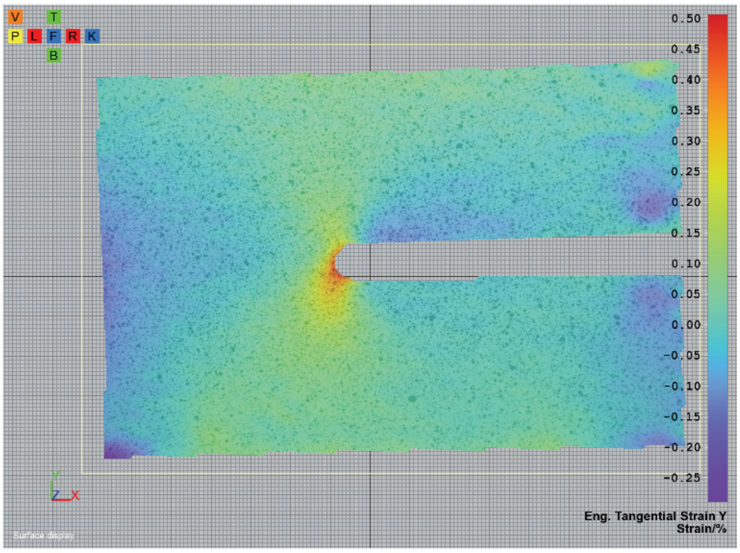

Figure 165 3D Visualization Orthographic Projections XY plane

In case the viewing direction is defined a Z coordinate axis, the XY plane is the projection from the front, the XZ from the top and the YZ from the side (Figure 166).

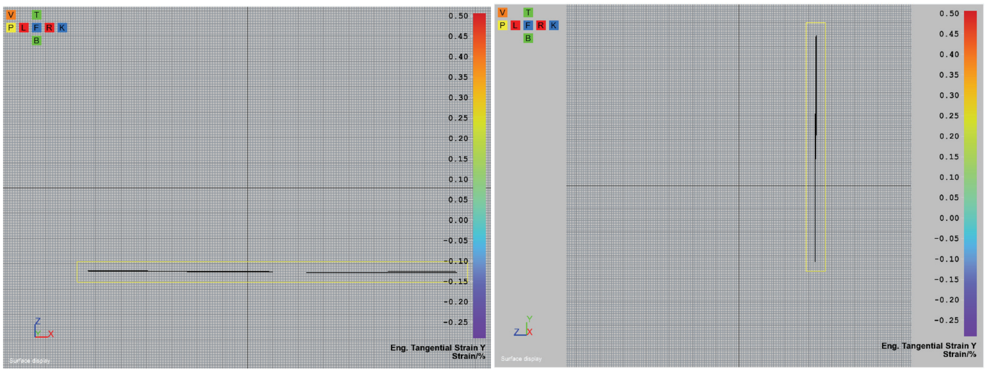

Figure 166 3D Visualization Orthographic Projections XZ plane (left, top view) and YZ (right, side view) plane.

8.6 Additional Features for Multi Camera Systems

In case of using the *Multi Camera Evaluation* with an object alignment additional options in the visualization are available (see page 92). These features are described depending on the object shape used.

8.6.1 Cylindrical Objects

If a cylinder is used as shape for the evaluation additional information for the displacement are available. Beside the displacement detriment in a *Cartesian Coordinate System* (X, Y, Z) here the displacements are available in a *Cylinder Coordinate system* (phi, Z, rho). The Z direction is in the direction of the axis of the cylinder, rho is radius and phi the angel (Figure 167).

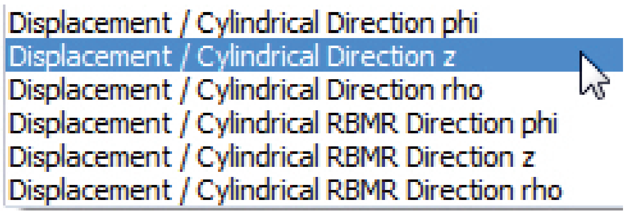

Figure 167 Available Cylinder Coordinate displacements

In order to be able to access this information it is required that the Object coordinate system is selected (see *Coordinates*). The following information is added to the list in the visualization section (see *Displacements*).

- **CYLINDRICAL DIRECTION PHI**: shows the displacement radial direction for every data point mapped as a color plot on the image.
- **CYLINDRICAL DIRECTION Z**: shows the displacement in direction of the axis for every data point mapped as a color plot on the image
- **CYLINDRICAL DIRECTION RHO**: shows the displacement in direction of the radius for every data point mapped as a color plot on the image

There are additional entries for the displacement with **REMOVE RIGID BODY MOVEMENTS (RBMR)**.

In the following Figure 168 the results of a measurement on a tire using a Multi Camera System are shown. In the upper image the displacement is displayed using a Cartesian Coordinate System. The selected Y direction is here from the center to the

measurement system. The lower image displays the same load but in a Cylinder Coordinate System. Here the radial direction is selected.

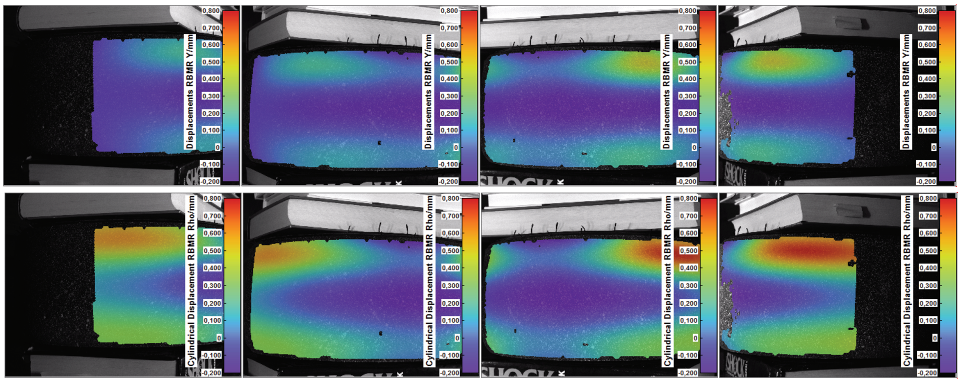

Figure 168 Comparison between Cartesian and Cylinder Coordinate System 2D

In the 3D visualization the tire is displayed in the Cartesian Coordinate System as half circle (Figure 169) as it appears.

In the Cylindrical Coordinate System however, as the evaluated area fits perfect with a cylinder, it is shown as a plane object.

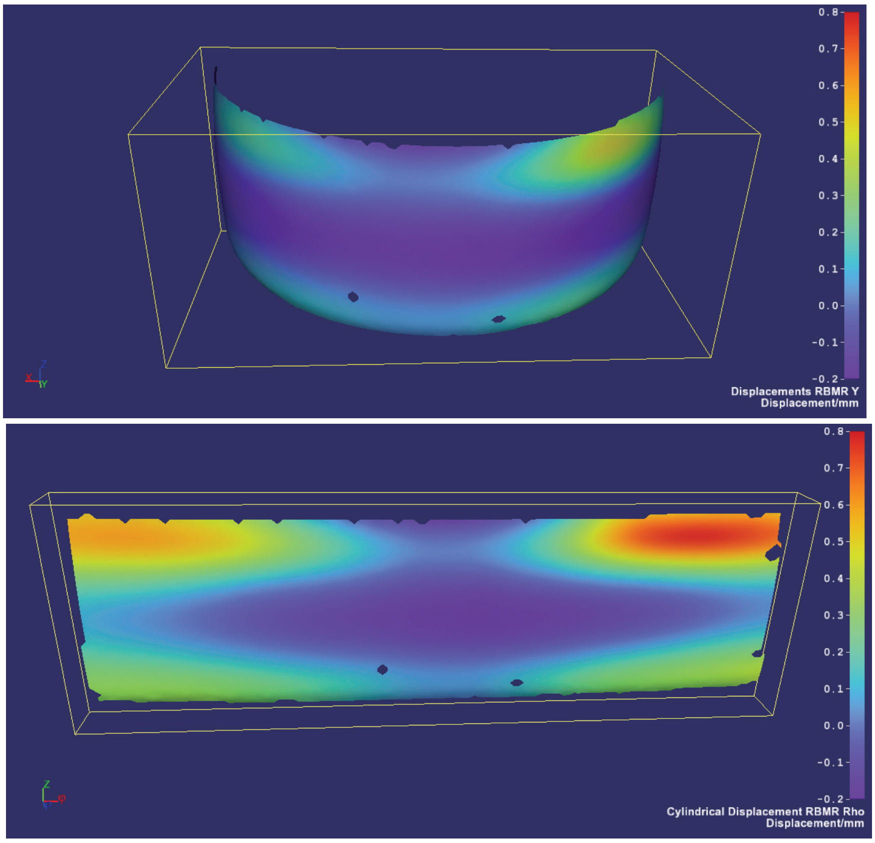

Figure 169 Comparison between Cartesian and Cylinder Coordinate System 3D

8.6.2 Bi-Plane Objects

A Bi-Plane object is a plane object where the front and the back side are measured simultaneously. A typical application for these types of objects is the measurement of a tensile test specimen. In addition to the full filed behavior of the front and backside also the changes of the surfaces relative to each other can be analyzed. And so also the thinning of the material can be measured.

A point element placed in the *Gauge* visualization on the evaluated area will show up on both sides of the object (Figure 170). A line or area elements are defined on one side only. The upper two images representing the front side, the lower images the backside of the sample.

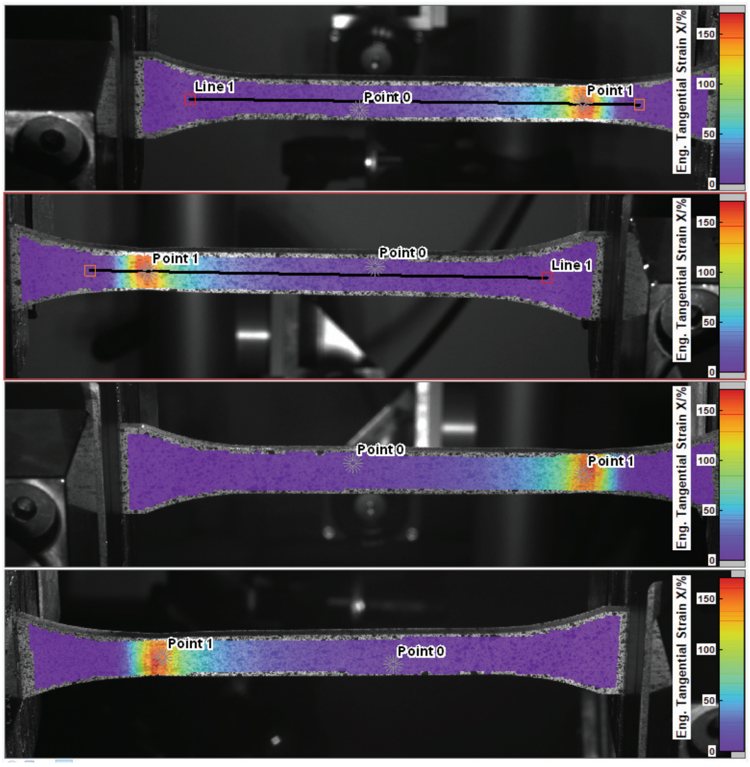

Figure 170 2D Visualization of a Bi-Plane object overlay

The thickness changes is available for all gauge elements. From this the strain in thickness direction and all the shear components can be determined as well. The additional available full field data are:

- **THICKNESS** shows the distance between the two surfaces at the actual step.
- **THICKNESS CHANGE** shows the change of the thickness between the reference and actual step.
- **TANGENTIAL STRAIN Z** shows the strain in z direction. It is calculated from the information of the two surfaces.
- **SHEAR STRAIN XZ** shows the shear strain in the XZ plane.

- **SHEAR STRAIN YZ** shows the shear strain in the YZ plane.
- **SHEAR STRAIN XY** shows the shear strain in the XY plane.
- **PRINCIPAL STRAIN 1** shows the maximum strain value.
- **PRINCIPAL STRAIN 2** shows the strain othogonal to principal strain 1 and 3.
- **PRINCIPAL STRAIN 3** shows the minimum strain value.

NOTE: *Data are only avilable from the surfaces and information between the surfaces con not be calculated*

These front backside points have additional entries in the Gauge tree (Figure 171).

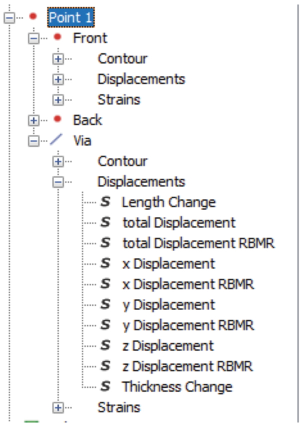

Figure 171 Visualization Bi-Plane Point

The standard point elements are available for the front as well as for the back side. In addition information between the points on both sides is calculated.

From the contour the distance between the positions on both sides is know. It can be used to determine an *Absolute Gauge Length*, corresponding to the distance at the actual step. The *Relative* distance corresponds to the distance change between the reference and actual step. In this way also the strain between these two positions as difference from the reference to the actual step can be calculated as *Global Gauge Strain (Figure 172)*.

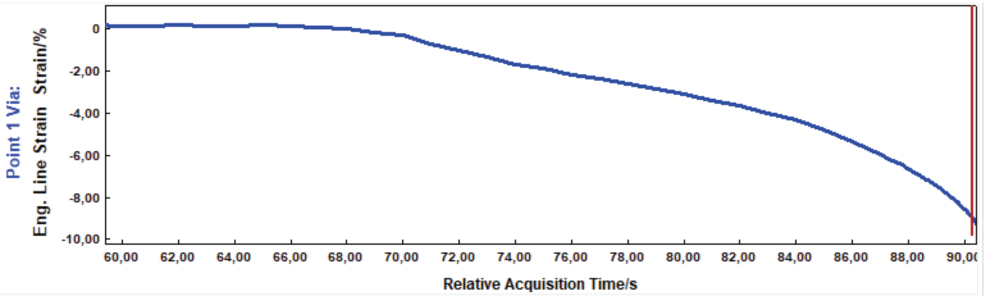

Figure 172 3D Visualization Global Gauge Strain temporal plot

In addition to the point information, from a line the thickness change (Figure 173) and strain (Figure 174) are calculated.

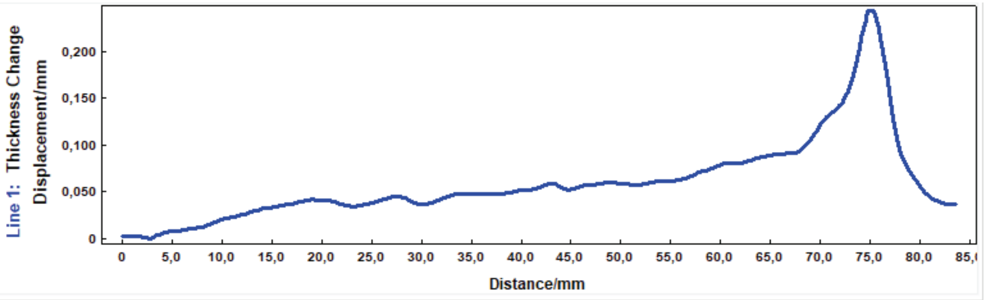

Figure 173 Spatial plot of the thickness change at single step

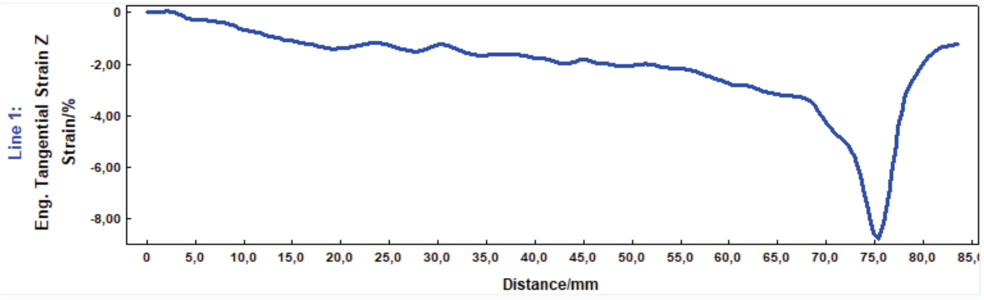

Figure 174 Spatial plot of the thickness strain at single step

In the 3D Visualization the object can be rotated and so both sides are accessible simultaneously. Figure 175 shows the measurement result of 1mm thick tensile test sample. The left image shows the front side, the middle the backside and the right image is a close up of the position of the Bi-Plane point.

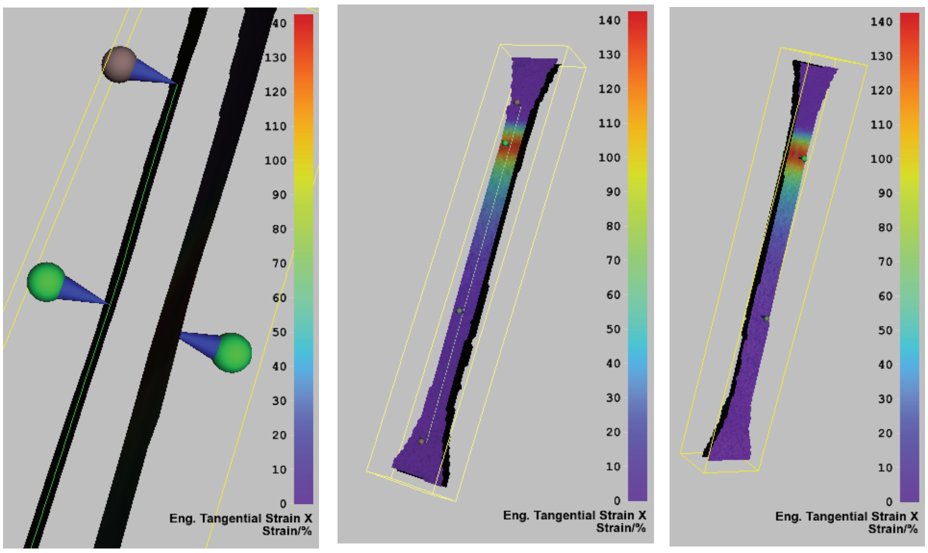

Figure 175 3D Visualization Bi-Plane

Using the front back side measurement with a tensile testing sample the thickness of the sample can be determined as full field data. In this way, the thinning of the sample during the test is available as well. And so the strain in all 3 dimensions, in-plane and thickness, can be calculated and displayed.

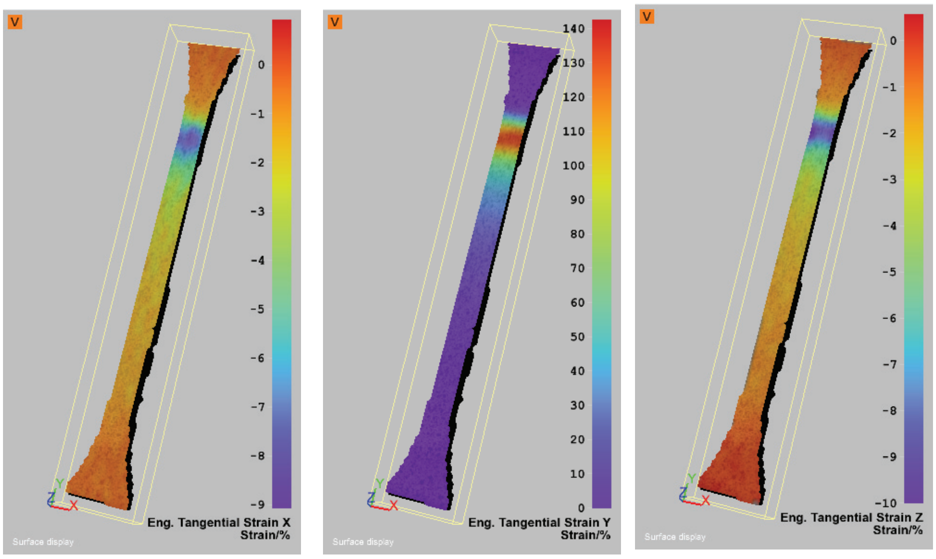

Figure 176 3D Visualization strain X, strain Y and strain Z

9. Real-Time functions[1]

In general in the digital image correlation technique in the first step the images are acquired, in following steps they are correlated to calculate the contour, deformation and strain and finally visualized for further analysis. Due to the developments in camera and computation techniques this sequential process can now be performed as a parallel process. That means while the images are streamed from the camera to the computer the correlation and reconstruction can be performed at the same time. This can be done in a way that the results are displayed in real-time on the computer screen during the experiment. Data from this can be used to generate an analog voltage output signal of a results value. In this way results from the correlation system can be used to monitor an experiment in real-time.

NOTE: *The maximum update rate for the real-time option depends on the hardware and the settings. In general it will be limited for full-field correlation in the range of a few Hz.*

This option is not suitable for the Q-450 High Speed system.

1. The Real-Time functions are an optional feature and requires the corresponding licence option.

9.1 Live Display Control

The *Live Display* View contains a *Multi Image Control View* (*Help Menu*) for presenting the image information and a control panel to the right (Figure 177).

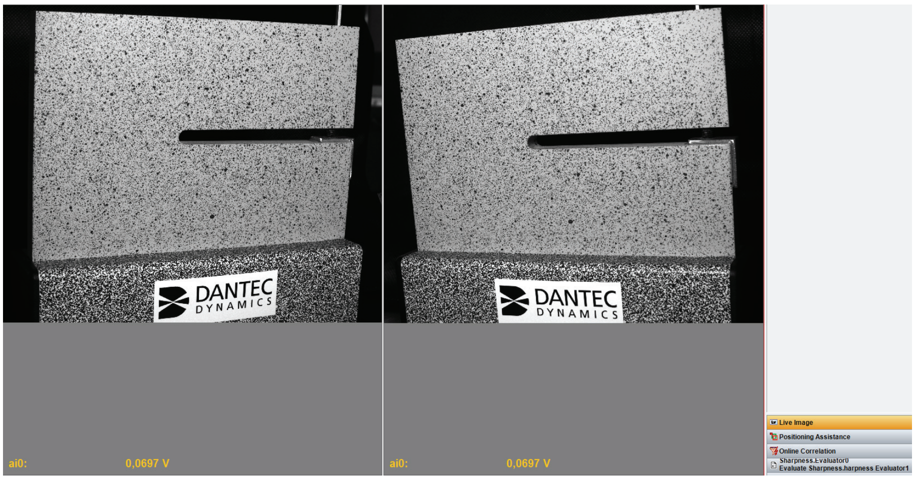

Figure 177 Live Display Camera Images

The control panel contains the following elements (Figure 178):

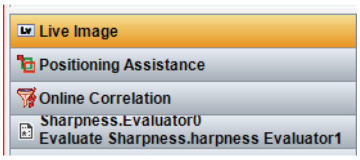

Figure 178 Live Display Control Panel

- *Live Image*: Displaying the actual live image of the cameras. In this view the different camera parameter are defined and the acquisition of measurement series is initiated.
- *Positioning Assistance*: This view helps to position the measurement system relative to the object in the same position as before.
- *Real Time Correlation*: Using this control a real time correlation of the images from the cameras is performed and displayed.
- *Sharpness Evaluator:* This view allows the judgment of the pattern quality with different correlation facet sizes.

9.2 Live Image

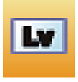

If a hardware configuration is activated (see also *Activation of Hardware*) and the Live Image view is started (see also *Acquisition of Images*) a Multi Image Control View (see also *Help Menu*) shows the live images of the cameras (Figure 177).

From this the different parameter of the cameras and display are defined as well as starting the acquisition modes. This view replaces the former Live Image View (see also *Live Image View*).

NOTE: *The following controls compare the actual live images to a reference image. Therefore they relay on the presence of a reference step. One can start a new series or continue an existing series (see also Acquisition of Images). The first step of the actual series is always the reference.*

9.3 Positioning Assistance

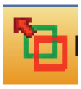

This control is designed to help the user to place the measurement system in the same position as during the reference step.

NOTE: *If a reference is not present because no image was acquired the positioning is not working. In this case the rectangles are not shown*

In this display the images of the cameras are shown and as an overlay the position of a rectangle in the reference step (green) and the actual positioned distortion of the corresponding rectangle (red) of the live image (Figure 179).

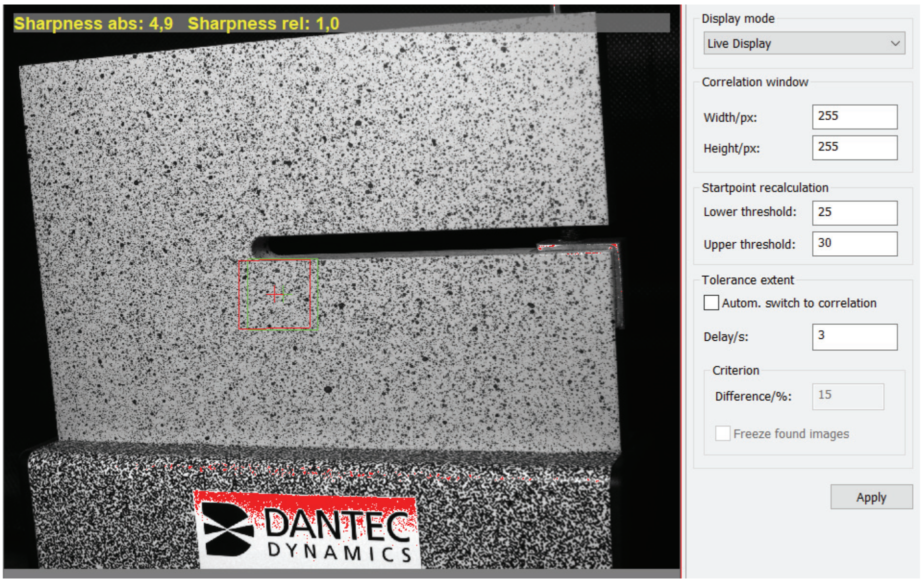

Figure 179 Live Display Positioning Control

The *Positioning Assistance* is based on the correlation of one large facet (analogical to the *Startpoint search*).

NOTE: *The Positioning Assistance requires the knowledge of the actual projection parameter. These are set with the Default Projection Parameter*

The *Sharpness* values in the upper part of the images indicate if the object and pattern have sufficient contrast and sharpness. The relative value compares to the reference step.

Tip: *This information can be used to set the system to an optimum focus. As this function requires no reference image it works without a reference image.*

If this view is activated several parameters are accessible in the control panel on the right side (Figure 180).

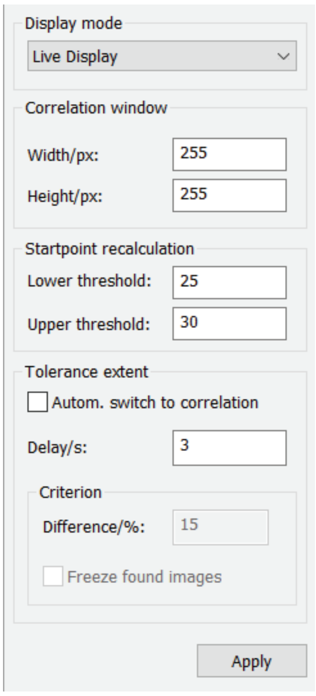

Figure 180 Live Image Positioning Parameter

The ***DISPLAY MODE*** can be changed between the actual live image or the reference image.

The size of this facet is defined by the parameter in the section *Correlation Window*. **WIDTH** and **HEIGHT** are the dimensions of this facet in pixels.

Tip: *The default values for the facet are 256 x 256 pixel. In case your object is smaller you may reduce these values. The smaller the value the more difficult it is to calculate changes in the distortion of the facet.*

The *Startpoint Recalculation* determines when a valid start point is found. The algorithm starts using the previous parameter. If the residuum[1] of the correlation is below the **LOWER THRESHOLD** the algorithm stops the search and uses the actual found Startpoint as the result. If the residuum is higher it will continue the search until a residuum below the **LOWER THRESHOLD** is found or it finishes when

1. The residuum is in Grey values (see also *Correlation Settings*)

the search in the whole area is competed. If within the whole area no *Startpoint* better than **UPPER THRESHOLD** was found the search is marked as not successful. Otherwise the *Startpoint* with the lowest Residuum is used.

Tip: *If the quality of the images is not good a higher Upper Threshold may help to find Start-Points.*

The default values for the Lower Threshold is 20 and for the Upper Threshold 30.

The section *Tolerance extent* controls an automatic switch between the positioning and the Real-Time Correlation. It's designed for the Hand-Held correlation system. The task is to minimize the difference of the position of the correlation system between the reference and the actual state. The positioning tool is optimized for a fast and robust detection of the shift between two images. With this feature it is possible to bring the correlation system back close to the original position. In this way the rigid body motion between the two states is minimized.

The function is activated by ticking the **AUTOM. SWITCH TO CORRELATION** box. The **DELAY** defines the time the system is waiting before it switches from the positioning into the Real-Time Correlation mode.

The definition of the distance between the two states is based on the ratio of the diagonals of the square respectively four-side figures and the distance of the centre. If the ratio differs less than the **DIFFERENCE** defines and the distance is within the search radius the actual position is set as close enough. The acquisition stops if **FREEZE FOUND IMAGES** is active. Otherwise the acquisition continues in the Real-Time correlation mode.

NOTE: *If the automatic switch is activated in the Positing Assistance the software will also switch back from Real-Time Correlation to Positing if it fails to correlate for a certain time.*

9.4 Real-Time Correlation

Like the Positioning Assistance the Real-Time correlation requires the presence of a reference image.

The Real-Time Correlation view is designed to perform and display a full field real time correlation of the actual camera images (Figure 181).

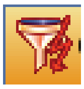

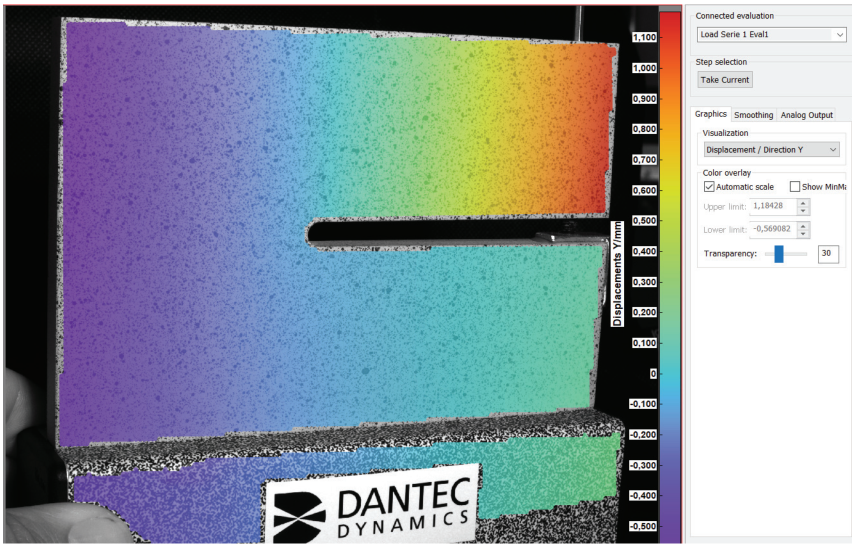

Figure 181 Live Image Real Time Correlation

The result is mapped as a 2D overlay on the live image of the reference camera. Since the display is within a Multi Camera Control View the user can select the display of the reference camera only to get a larger display of the results.

9.4.1 Start a Real-Time Correlation

A Real-Time correlation is based on an existing series of images and a corresponding evaluation. In order to perform a correlation several settings and parameters are required. At first the actual image acquisition must continue an existing image series (*Manual Acquisition*). The next step is the connection of the Real-Time Correlation with an evaluation (Figure 182).

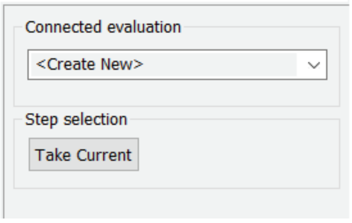

Figure 182 Live Image Real Time Correlation Connect

The series to connect to is selected in the *Connected Evaluation* section. As the first entry in the drop down list the selection to **CREATE A NEW SERIES** is listed. Selecting this will generate an

evaluation series using the default correlation parameter. If an evaluation already exists this can be selected from the drop down list (Figure 183).

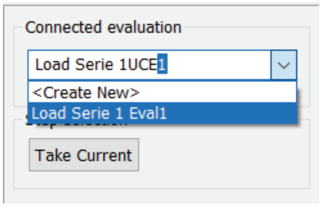

Figure 183 Live Image Real Time Correlation Connection List

Tip: *In order to make sure that the Real-Time Correlation does work properly it is recommended to generate first an evaluation (Start a Universal Correlation Evaluation) and set the parameter in a way that the correlation gives good results.*

The section *Step Selection* defines how the update is done. If **CONTINUOUS UPDATE** is marked the update is done as fast as possible. Otherwise the update is only done if the **TAKE CURRENT** button is used.

9.4.2 Visualization

The *Visualization* section contains multiple tabs for further functionality.

Graphics

In the Graphics tab a drop down list shows all available types of data to be displayed (see also *2D Image Overlay*).

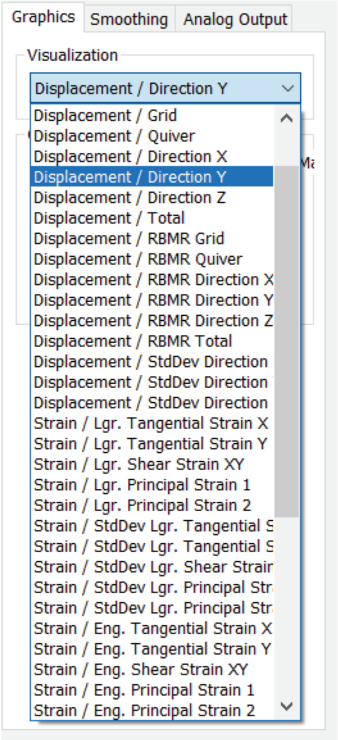

Figure 184 Live Image Real Time Correlation Graphics

As in the standard 2D overlay visualization the scaling of the display can be set to automatic or fixed values.

NOTE: *The real time correlation process is optimized in order to use multi core processors in an optimum way. Never the less one will realize a delay between the load and the display which is higher than the maximum update rate.*

Tip: *In order to maximize the update rate as well as to minimize the delay between loading and the display the following parameters can be adjusted:*

- *Reduce the number of pixel in the images by using the ROI function (see also Definition of Region of Interest (ROI))*
- *Increase the Grid spacing (see also Correlation Settings)*
- *Reduce the Facet size (see also Correlation Settings)*
- *Keep only one image of the reference camera in the display*

Smoothing

The smoothing defines if and how the Real-Time data is smoothed for the display.

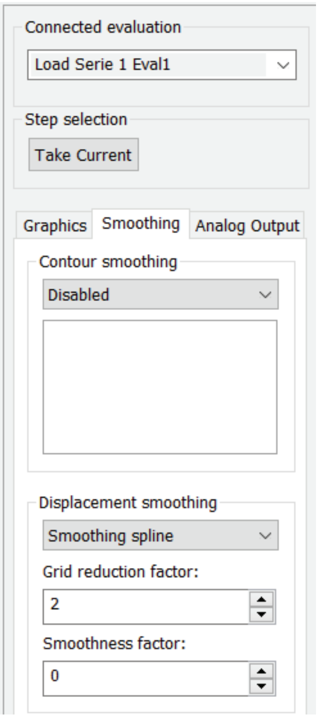

Figure 185 Live Image Real Time Correlation Smoothing

The settings are identical to the standard visualization. For more details see *Smoothing*.

Tip: *In order not to loose performance its recommended to use the smoothing spline filter only*

Analog Output

With the Analog Output function it is possible to convert a scalar data from the result of the Real-Time correlation into a voltage and send it to an Analog Output channel of the trigger box. The

definition of the data is done in the Gauge tab the standard visualization (*Gauge*). Every scalar entry from the report window can be used as Output signal.

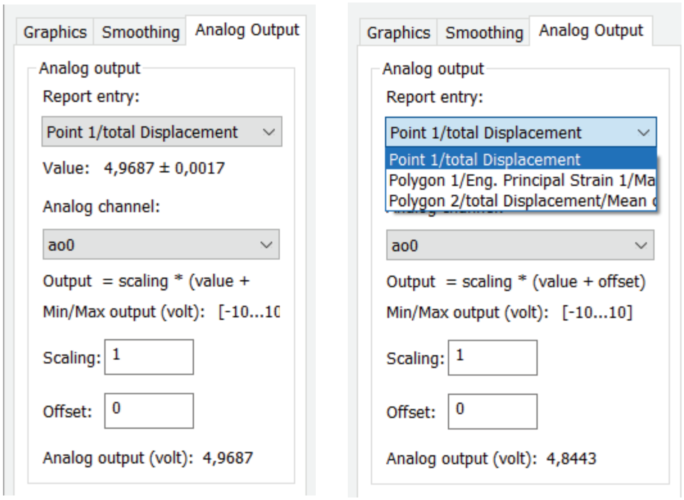

Figure 186 Live Image Real Time Correlation Analog Output

The **REPORT ENTRY** drop down list shows all available data which can be used as an output value. The channel to use can be selected from the **ANALOG CHANNEL** list. In order to adapt the range of the output voltage signal the correlation results can be shifted by an **OFFSET** and scaled by the **SCALING** value. The value of the output channel in Volts is displayed in the lower part.

NOTE: *This feature is designed to monitor a test in real-time.*

It is not designed to control any other device!

9.5 Pattern Quality Evaluator

The Pattern Quality Evaluator is a tool for real-time qualification of the object pattern. Depending on the selected **FACET SIZE** the reachable correlation accuracy is calculated. The display is divided in three regions (Figure 187).

All areas which can be calcualted with an accuracy better than the **STD ERROR MIN** are displayed in yellow color. The interval between the **STD ERROR MIN** and **STD ERROR MAX** is displayed from green to blue color. Areas with an expected error more than **STD ERROR MAX** are not color coded.

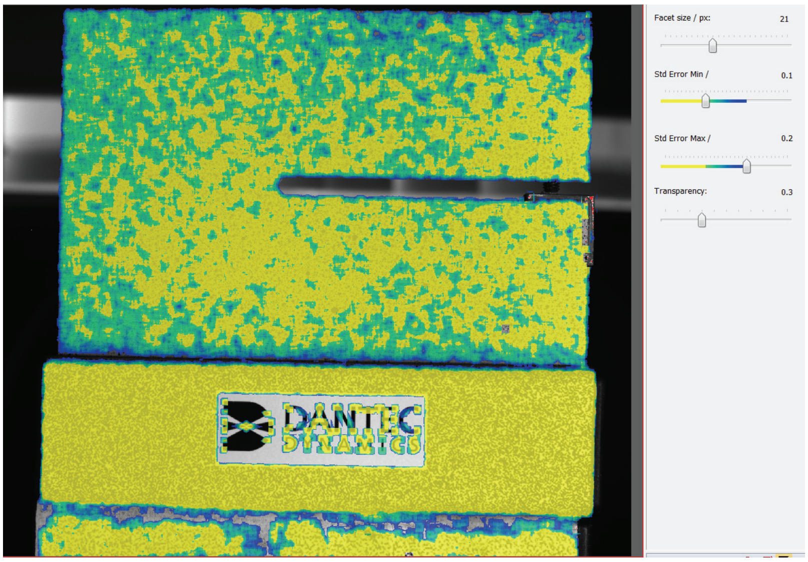

Figure 187 Live Image with Pattern Quality Overlay

NOTE: *The image quality is calculated based on the sharpnes and feature quality of the image.*

Overexposed areas are not color coded.

The **TRANSPARENCY** defines the visibility of the image of the object.

10. Export Functions

This Chapter describes the different functions to export the data and information from **ISTRA 4D** in order to be used with other software packages to generate reports (e.g. Microsoft Office) or do other post processing procedures (e.g. MatLab).

Functions related to the Gauge Elements are already described within the Gauge section in *Display Gauge Data*.

10.1 Export of Images

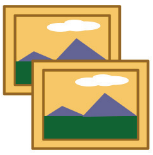

The *copy active graphic to clipboard* icon copies the camera image displayed into the clipboard.

If camera images are displayed the active camera image can be saved as *tif, bmp* or *png* bitmap image on the hard disk using the File-*Export-Graphic* function (Figure 190).

NOTE: *The image is exported as it is displayed including Step, Time and Analog Data information.*

The image of only 1 camera can be copied to the clipboard

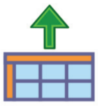

The camera images off all cameras and all steps can also be saved as individual *tiff* bitmap files. If the visualization of the series of images is active the *Data Export* icon or the corresponding menu function (*File-Export-Data*), opens a dialog to configure the export of the images (Figure 188).

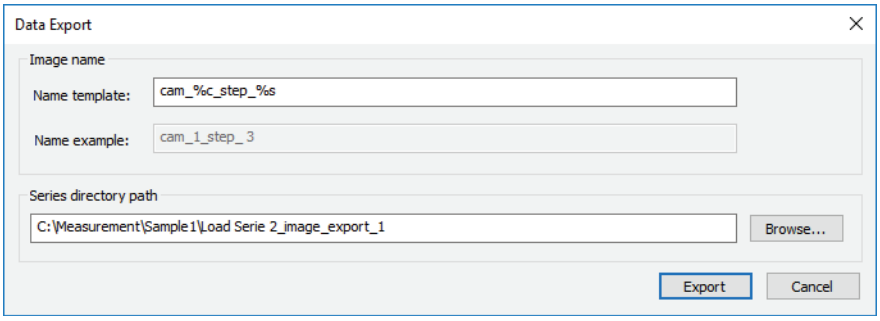

Figure 188 Export Image Series

The naming of the files is defined in the **NAME TEMPLATE** field. As wild cards for the number of the camera and the number of the step the variables %c and %s are used. The storage directory is set in **SERIES DIRECTORY PATH**.

The images and graphics can also be exported as an AVI file and saved by using the *File-Export Movie* function (Figure 189).

In the *Selection* section the images and graphics to export are defined. The drop down menu contains various selections. Using **SELECTED VISUALIZATION** only the selected window (marked with red frame) is used, **VISIBLE VISUALIZATION** uses all displayed windows, **ALL VISUALIZATION** uses all available windows, even if they are not displayed and **NO VISUALIZATION** uses the plots only. If **FULL RESOLUTION** is activated the images are copied in full resolution, otherwise the actual display settings are used. By selecting **SPATIAL** or/and **TEMPORAL PLOT** these plots are added to the exported windows. The image on the right side shows a preview for the selected settings. With the slider below the images for the selected step are displayed.

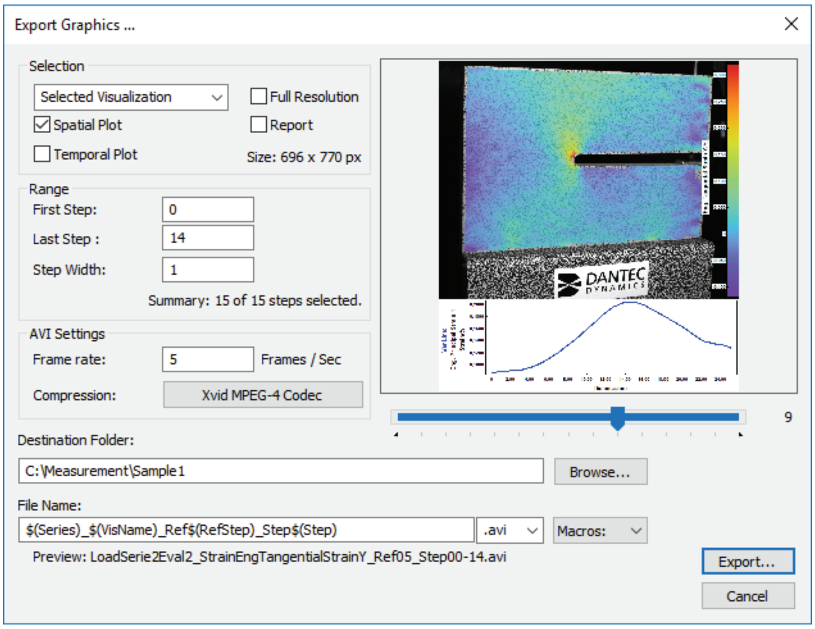

Figure 189 AVI File Configuration Dialog

In the section *Range* the steps to be exported are defined. The images in the movie starts with **FIRST STEP** to **LAST STEP** and **STEP WIDTH** determines if each image or only a portion is used to create the movie.

The parameters for the AVI creator are selected in the section *AVI Settings*. **FRAME RATE** selects how many images are displayed per second, the **COMPRESSION** defines the algorithm used to create the AVI file. Using the *Browse* button allows the selection of available algorithm (encoders). Depending on the encoder different parameters for the conversion can be defined.

Destination Folder selects the folder where the movie is saved. The *File Name* defines the name of the movie file. The file name can be typed manually or compiled from various variables. A selection of the available variables is in the *Macro* list. A preview with the actual settings is shown below.

NOTE: *An AVI creator consists of an encoder and decoder. The encoder compresses the views and creates the avi file. The decoder is required to read the avi file and show the movie. To show this movie on other computers the corresponding decoder needs to be installed.*

Tip: *We recommend the free AVI creator Xvid, which generates small files with good quality. (http://www.xvid.org/)*

10.2 Export of Evaluated Data

Depending on the status of the active data set different functions are available from the Icons in the toolbar and the *File Menu* (Figure 190).

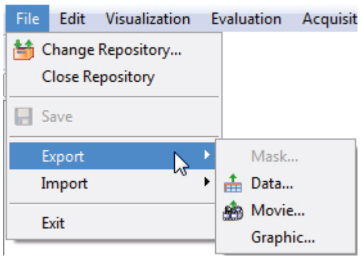

Figure 190 File Export Menu

Export as Visualization

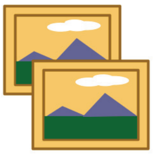

The display of the visualization can be exported as a graphic via the clipboard. The corresponding *copy active graphic to clipboard* icon copies the contents of the Visualization as displayed as a bit-map graphic onto the clipboard. Using the standard paste functions this information can be pasted into different software.

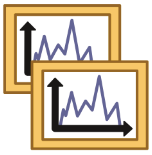

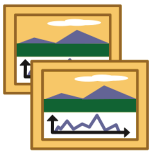

If additional data are in the plot window, two more icons are availbale. The *copy active plot to clipboard* icon copies the visible plot as bitmap graphic into the clipboard. By using the standard paste functions, this graphic can be paste in other software. Both graphics, image and plot, can be copied into the clipboard by using the copy active graphic and plot to clipboard icon.

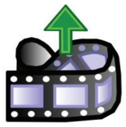

In addition the displayed results can also be copied as a series of images in an AVI movie to the hard disk. The function is identical to the function for the images (*Export of Images*). Since the results are copied as seen on the screen with all the active settings the user needs to set the type of data, filter parameter etc. before activating this function.

Export as data

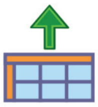

The results of the active data set can be exported as data to a file in various different formats. The activation of the corresponding Export Data Icon opens a dialog where the format can be selected from the list *Select the export format*. For each format a short description is given below.

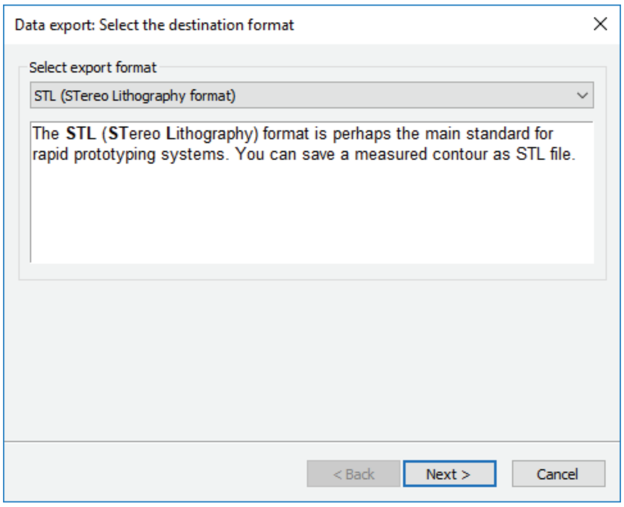

Figure 191 Dialog Select Data Export Format STL

With the **STL** (STereo Lithography) format only the contour information of one single measurement step is exported (Figure 191). If this format is selected the *Next>* button opens a new selection where in *Data Selection* the type of contour is chosen. The possible contours are the **REFERENCE CONTOUR**, the **SMOOTHED REFERENCE CONTOUR** and the actual **STEP CONTOUR**. The name of the STL file is specified in the *File name and path* section.

The **SNAPSHOT AS HDF5 SERIES** saves all the data of the active data series as files in HDF5 format (Figure 192).

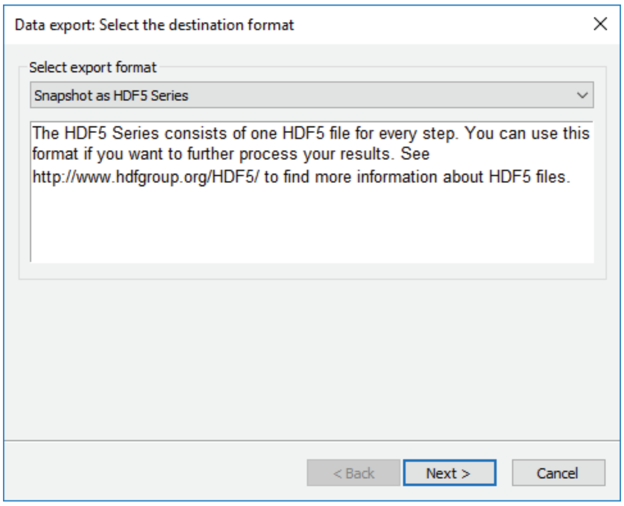

Figure 192 Dialog Select Data Export Format HDF5

All different types of available data are exported for each step, e.g. contour, displacements and strain data. The data is saved according to the selected settings of the visualization, like the active coordinate system and smoothing parameter (*Visualization of Evaluated Data*).

The *Next>* button opens a new selection, where in the section *Step selection* the steps to be exported and the directory where the files are saved is specified (Figure 193). For each selected step a file is created called series_step_*nr*.hdf5, where *nr* is the step number. The directory to be used is set at *Series directory path*.

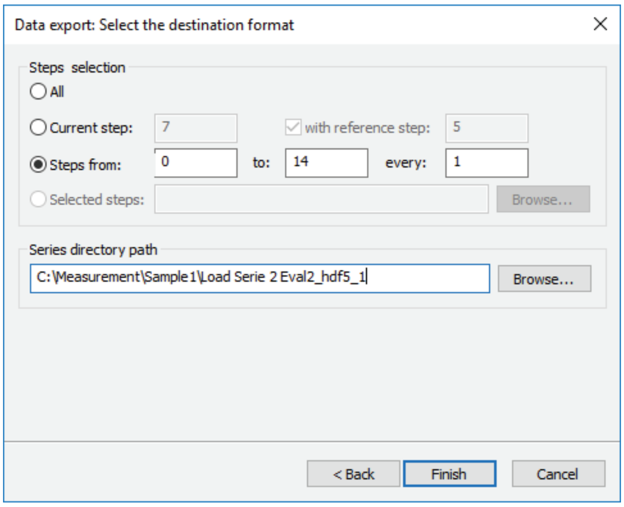

Figure 193 Dialog Select Data Export Format Step Selection

NOTE: *The HDF5 file format is Hierarchical Data Format with open specifications (see also: www.hdfgroup.org). This data can be opened by other software e.g. MatLab for post processing. This gives the user full access to all data calculated with the software.*

During the evaluation process the data is also saved automatically in the HDF5 file format. But in these files the system saves pre-evaluated data. From this the data is calculated on the fly for the visualization according to the settings.

The data exported using the Snapshot format is only for further post processing with other software. It's not possible to import them later.

The **ASCII** format generates for each step an ASCII file containing all valid data points at this step (Figure 194).

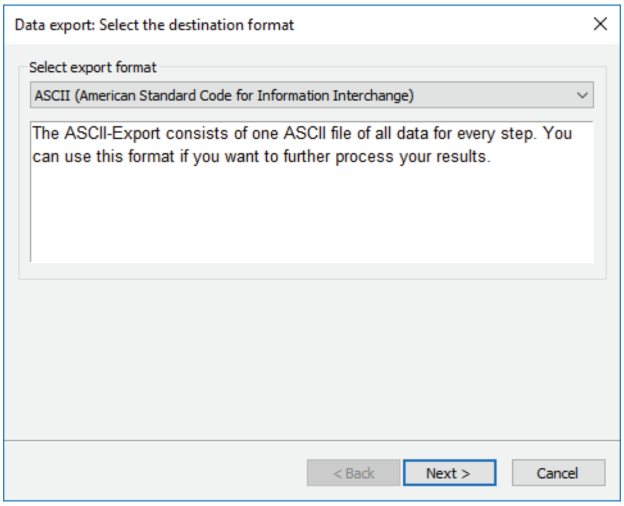

Figure 194 Dialog Select Data Export Format ASCII

The *Step Selection* section (Figure 193) allows the specification of the steps to be exported.

The first two columns showing the index of the data point in the evaluation grid. Followed by all available data values of this point. The columns are separated by Tab and the decimal delimiter is selected according to the settings of the operation system.

11. ISTRA 4D Options

The ***ISTRA 4D*** *Options* Icon or corresponding function in the *Tools Menu* opens a dialog with different options. In the left part the functions are grouped around different topics.

11.1 Environment

11.1.1 Unit System

In the *Units* section (Figure 195) the units for the display of the different quantities is defined. The units can be defined for *Position*, *Displacement* and *Strain* independently. In addition the units for *Angle*, *Phase* and *Time* can be defined.

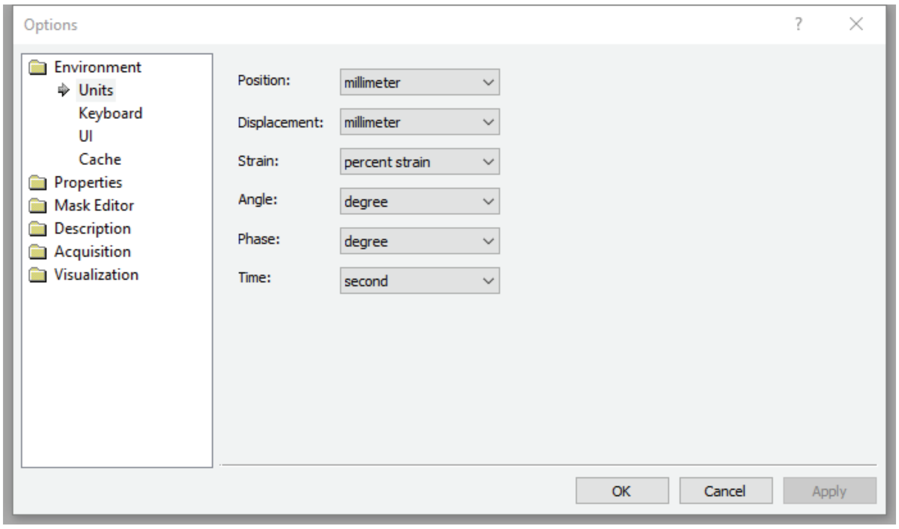

Figure 195 Dialog Options Environment Units

For the *Position* and *Displacement* the following units are available:

- millimeter, micrometer, nanometer, decimeter, centimeter, meter, inch, foot

As units for *Strain* are available:

- millistrain, strain, microstrain, percent strain, promile strain

As units for *Angle* and *Phase* are available:

- degree, revolution, radian

As units for *Time* are available:

- second, millisecond, microsecond, nanosecond

11.1.2 Key Shortcuts

In the *Keyboard* section of the group *Environment* (Figure 196) the user can define key shortcuts for different functions in the software.

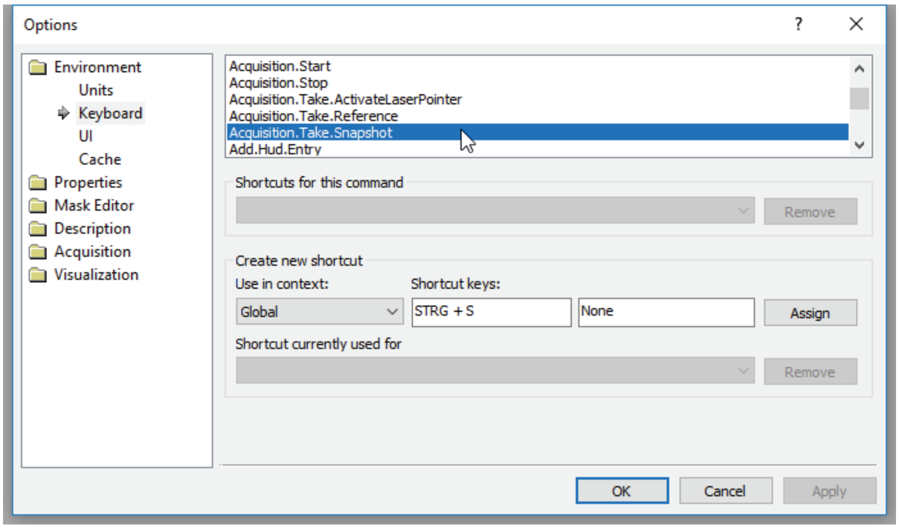

Figure 196 Dialog Options Environment Keyboard

In the top of the right part a list with all accessible functions. For the selected function in the section *Shortcuts for this command* the actual shortcut is shown, if this exists. This assignment can be deleted by the **REMOVE** button.

To create or change the shortcut for the selected function, click with the mouse in the field *Shortcut Keys* of the Create new shortcut section. Now press the sequence of keys which should be connected with the function. Use the **ASSIGN** button to activate this shortcut. If the Shortcut is currently used for a selection the actual use of the shortcut is shown, if this already exists.

NOTE: *A shortcut can be assembled by a single key, combination of keys, e.g. Alt+F4 or sequences e.g. Alt+A and S*

11.1.3 UI

Inthe *UI* section of the group *Environment* (Figure 197) the user can define the size of the icons in the tool bar.

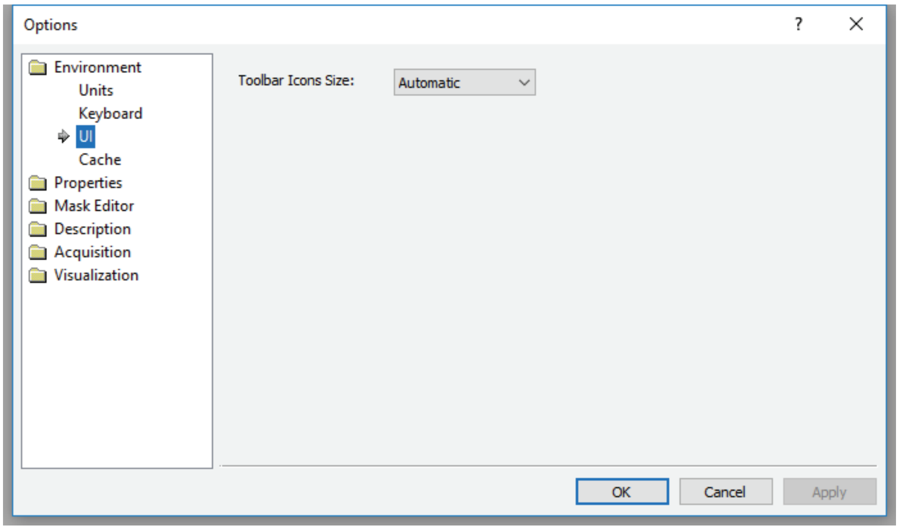

Figure 197 Dialog Options Environment UI

11.1.4 Cache

ISTRA 4D uses a cache on the hard drive in order to store results of filtered data when they are calculated. This improves the speed for the display and export of filtered data. The maximum size for this cache is defined by using this setting.

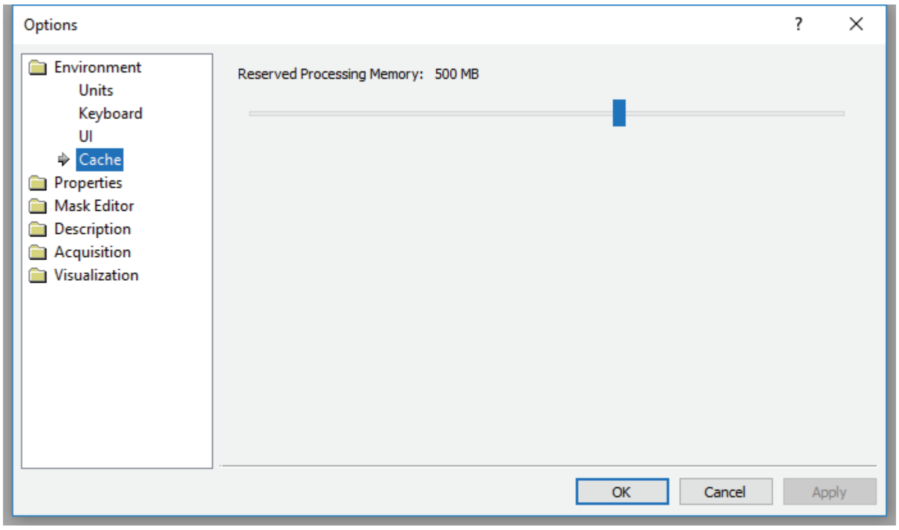

Figure 198 Dialog Options Environment Cache

11.2 Properties and Descriptions

The Properties and Descriptions function are intended to give the user a very flexible way of connecting property information in a strict hierarchical way to the repository and it's elements. From this information reports can be generated to the individual elements.

The highest level are Properties and Descriptions which are linked to the program itself. Below this level is the repository and under this are the elements of the repository.

If a new element is created it inherits the Properties and Descriptions from the higher level, e.g. a new repository inherits the definitions from the program.

11.2.1 Properties

The *Properties* of the program are defined using the *Properties* entry of the dialog *Options Properties* (Figure 199).

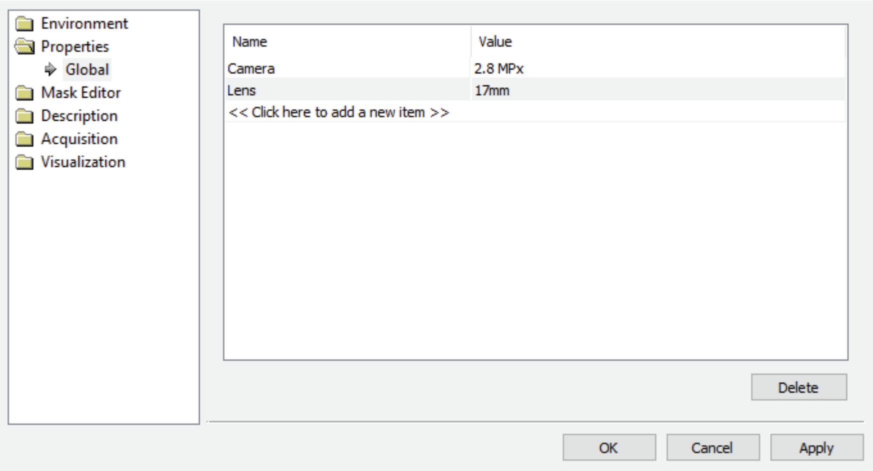

Figure 199 Dialog Options Properties

The elements are global and belong to all elements of the repository. The dialog allows the user to create new elements using the **NEW** button or delete elements using the **DELETE** button. In the left column the name and in the right column the value of the element is defined. The **APPLY** button saves the settings without closing the dialog. Each entry must be accepted by **APPLY** before entering a new item.

To change the Properties of an existing repository or an element within use the right mouse to open a context sensitive menu. Here the function **CHANGE PROPERTIES** opens a dialog where existing

elements, e.g. inherit during the creation, can be changed or new elements can be defined similar to the Options Properties dialog (Figure 200).

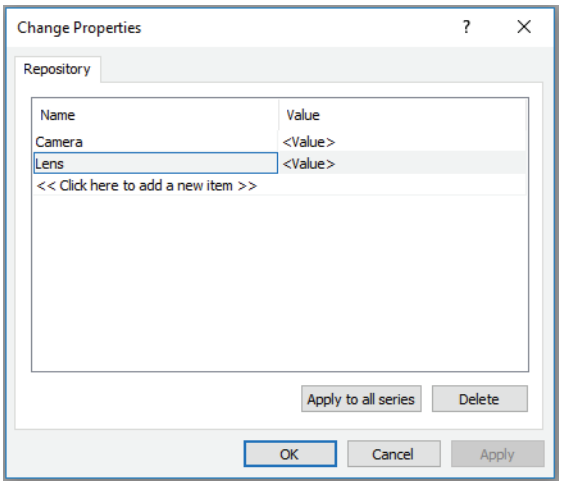

Figure 200 Dialog Change Properties

11.2.2 Description

The structure of the *Description* is the same as the *Properties*. Using the *Description* the user can generate a text including the defined corresponding *Properties* (Figure 201). Where the *Properties* are used as variables which are dissolved when using the description.

In the window of the *Description* section text can be edited. To add properties use the **INSERT PROPERTIES** button. In the dialog the default and user defined properties are listed and can be inserted in the Description (Figure 202).

The property appears in brackets with a leading *$-sign* in the upper text window. For each *Description* a name can be given in the list element **NAME**. Here the user can also select an existing definition.

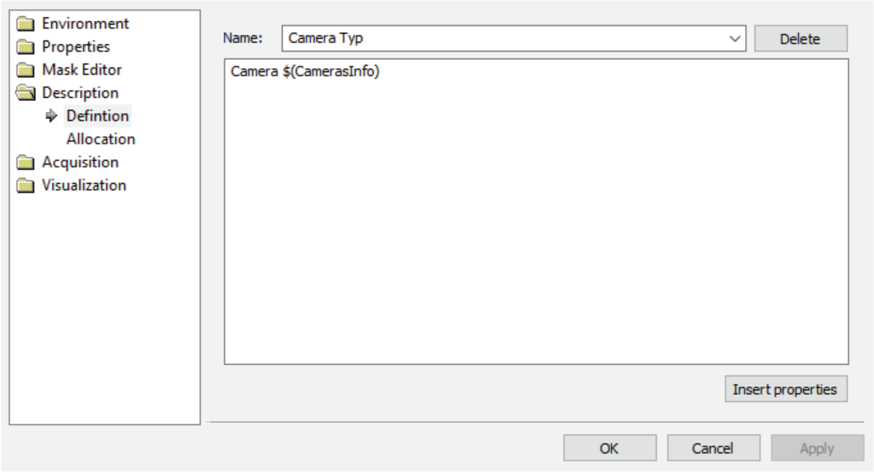

Figure 201 Dialog Options Description

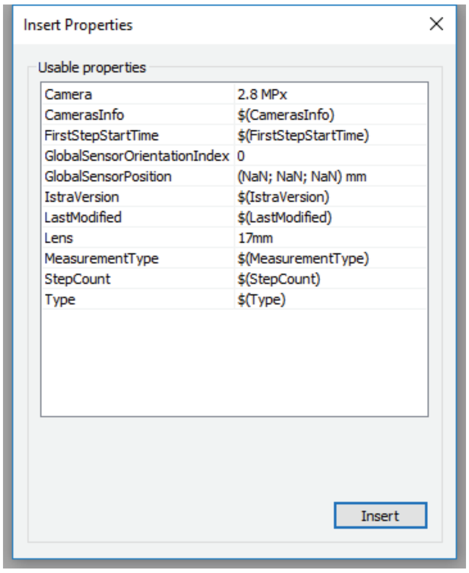

Figure 202 Dialog Insert Properties

The **ALLOCATION** selects where the descriptor information is displayed (Figure 203).

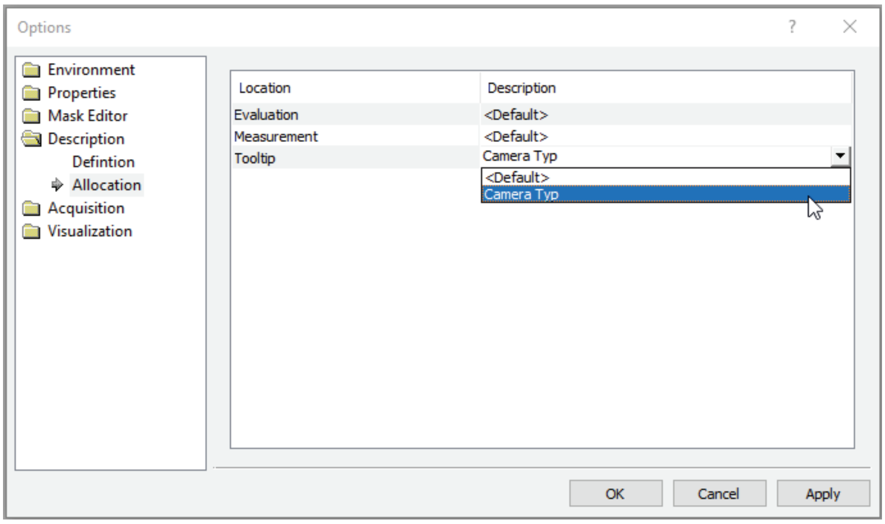

Figure 203 Description Allocation Dialog

NOTE: *Under Description Measurement appear the properties defined in General as well as defined in Measurement.*

To change the Description of an existing repository or an element within use the right mouse to open a context sensitive menu. Here the function **CHANGE PROPERTIES** open a dialog where existing elements, e.g. inherit during the creation, can be changed or new elements can be defined similar to the Options Descriptions dialog.

NOTE: *If the Properties or Description are changed these changes are not applied to the existing elements, they effect only new elements created after.*

11.3 Mask Editor

In the section *General* of the *Mask Editor* section the user can define the color which is used to draw the mask as an overlay of the image (*Mask Definition*) and the start points in the start point search (*Startpoint search*).

Parameter	Description
**DEFAULT**	Color of the non active start points in the other cameras and steps
**SELECTED**	Color of the active start point in the other cameras and steps
**ACTIVATED**	Color of the active start point in the reference camera at reference step. And the color of the mask
**EXTRA**	Color of the non active start points in the reference camera at reference step

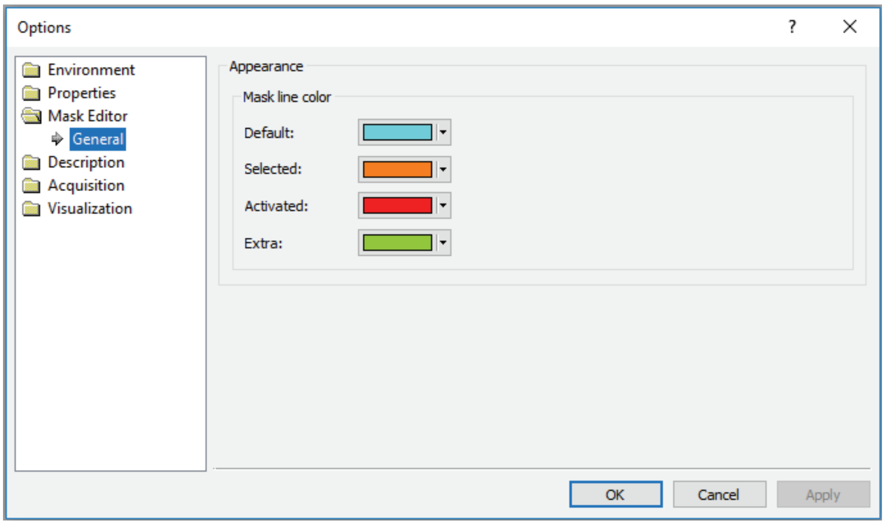

Figure 204 Options Select Mask and Start Point color

A mouse click on the actual used color opens a pallet from which the color to be used can be selected.

11.4 Acquisition

Within the *Acquisition* settings it is possible to activate for each recording and storage of an image to the harddisk a confirmation by the user (Figure 205). If the *Storage Request* is active the user is asked before an image is saved in the active series.

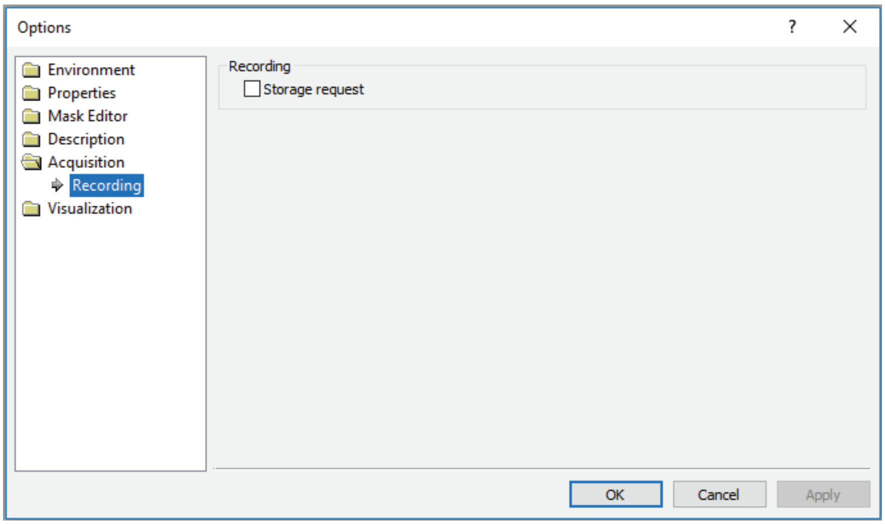

Figure 205 Dialog Options Acquisition Recording

NOTE: *This option is designed to be used with the Q-480 Handheld sensor.*

11.5 Visualization

In the *Visualization* section differend parameters controlling the display of graphical elements can be defined.

11.5.1 Appearence

Within the *Appearence* settings the background color of the *3D Model Visualization* is defined (Figure 206). By using the standard color definition tool the color of the 3D visualization can be selected.

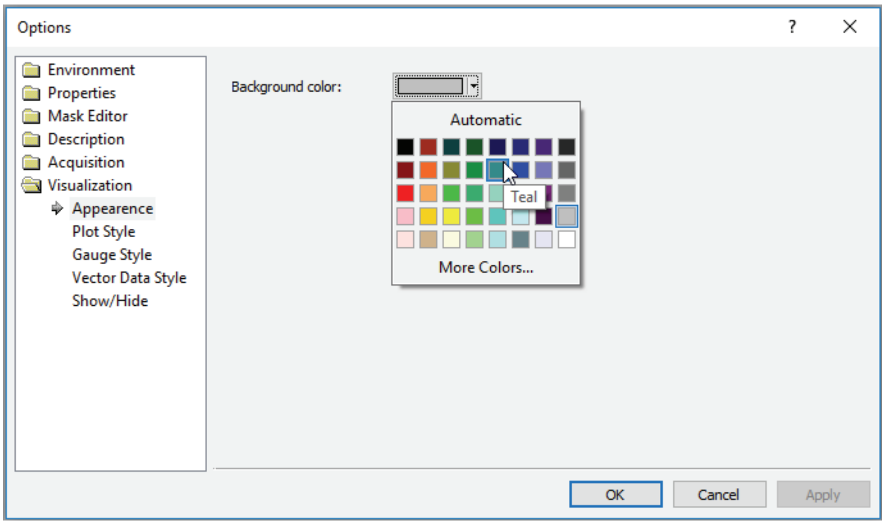

Figure 206 Dialog Options Visualization Appearance

NOTE: *The background color can't be changed on the fly. In order to apply the changes the 3D Visualization must be reopen.*

11.5.2 Vector Data Style

The *Vector Data Style* selection defines the images which is used for the *Grid* and *Vector* display (*Grid or Vector Display*). If the *Reference Image* is used the Grid of Vectors are displayed on the reference image. The beginning of the Quiver is fixed at the reference position and the arrow shows the position at the current step (depending on the selected scaling). If the *Current Step* is selected, the image of the current step is used. The end of the arrow is fixed at the position of the data point at the current step and the beginning shows the position in the reference step (depending on the selected scaling).

Arrow Width changes the width of the quiver.

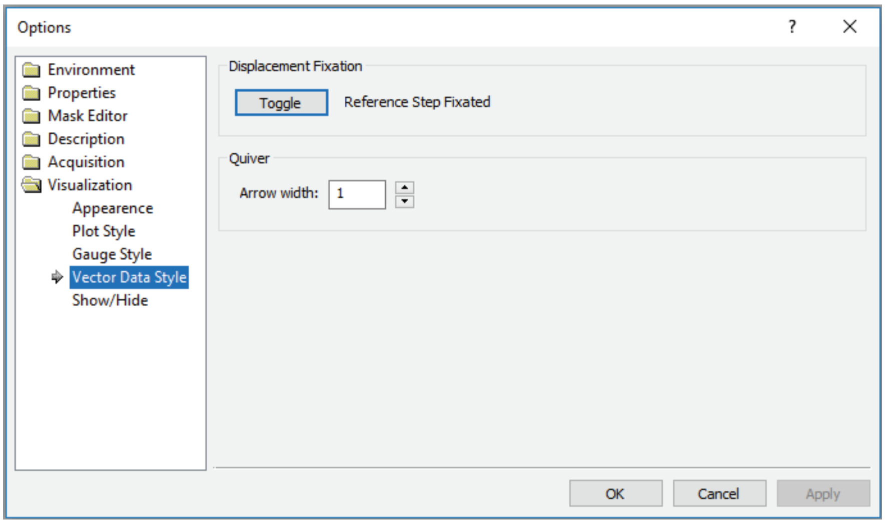

Figure 207 Dialog Options Visualization Vector Data Style

11.5.3 Plot Style

The *Plot Style* section defines the display of the *Spatial* and *Temporal Plots* in the visualization (*Display Gauge Data*). The Curve styles defines the colors and width of the curves. The *Marker* set the color used for the indication of the *Reference* and the *Current Step* in the *Temporal Plot*.

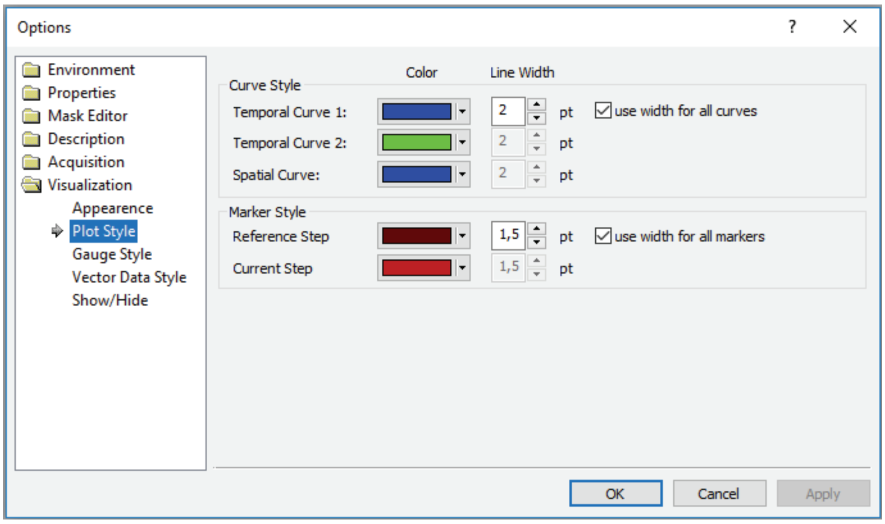

Figure 208 Dialog Options Visualization Plot Style

11.5.4 Gauge Style

The *Gauge Style* section defines the display of the Gauge Elements (*Gauge*). The Points and Markers can be diplayed as *Default* or the display can be selcetd from *Star*,*Square* and *Circle*. In this case the color and the width can be defined as well. For Line Elements the color and width can be defined.

If the **OVERLAY VISIBILITY** is activated, the name of each gauge element is labled and the name from the gauge tree is drawn in the image (*Gauge Data Labels*). The font and the color of the labels can be defined. To each label an outline can be selected. It can be a rectangular box or an oultine defined by the text. The size and color can be defined as well.

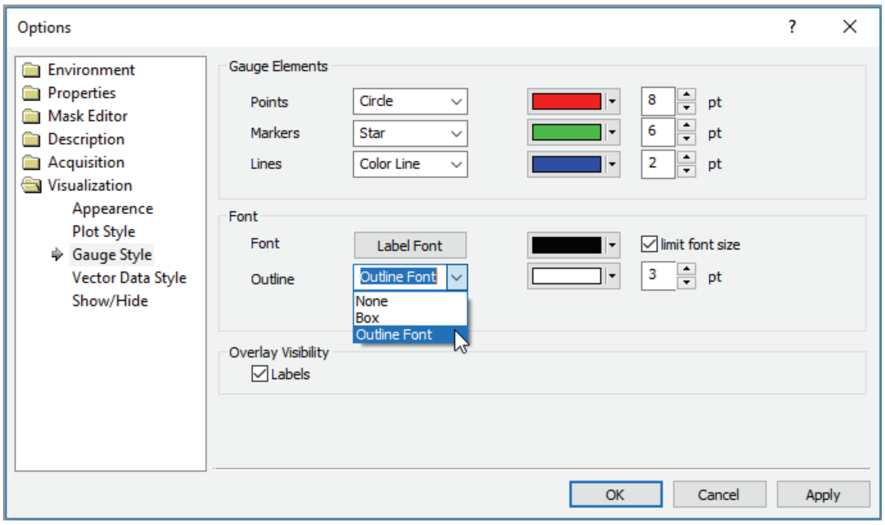

Figure 209 Dialog Options Visualization Gauge Style

11.5.5 Show/Hide

Within the Show/Hide selection the elemetns to be displayed in the Visualization list (*Graphics*) can be restricted. With the *Strain Measures* the definition of the displayed strain calculations can be selected.

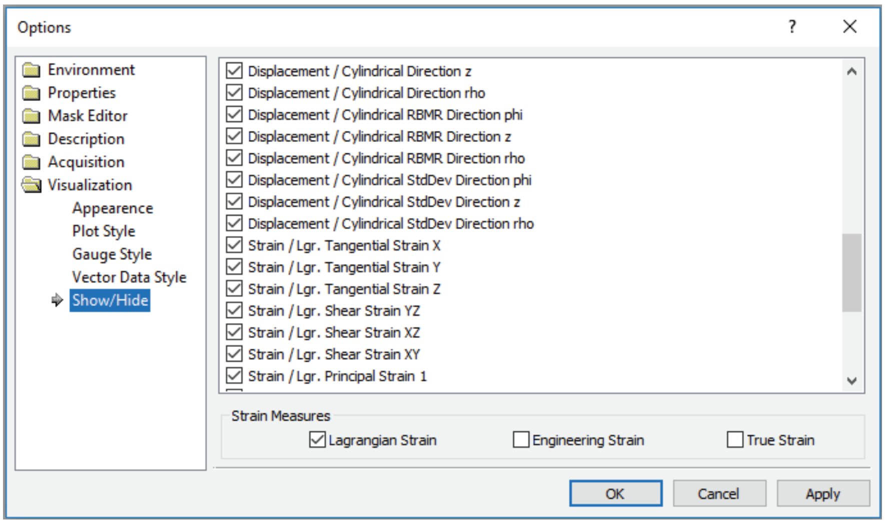

Figure 210 Dialog Options Visualization Show/Hide

11.6 Extensions

If the **ISTRA 4D** is started in administrator mode an additional option *Extensions* is available. In this section the path to external software packages used is defined. By using the Add button new search path can be added. This modification is active after a restart of **ISTRA 4D** only.

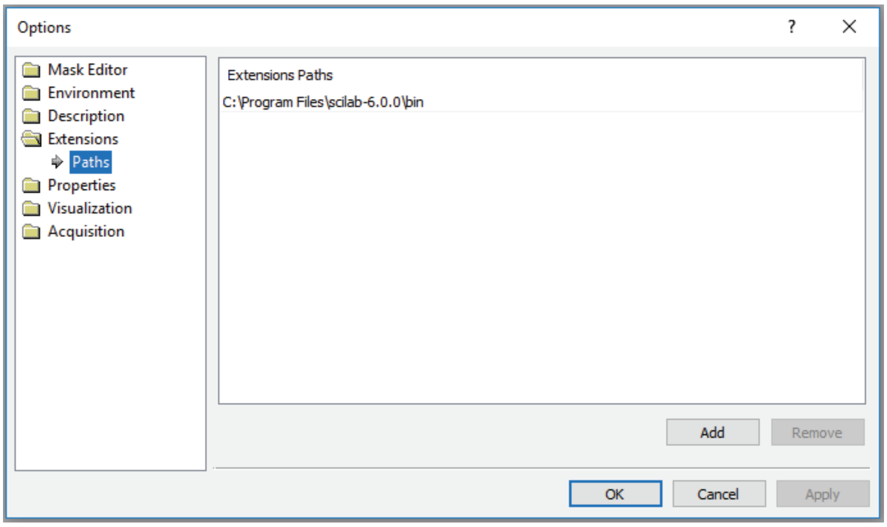

Figure 211 Dialog Options Extensions Path

NOTE: *This options is needed only if a new external software package is used.*

12. Miscellaneous

Some more generic options of the **ISTRA 4D** software are described in the following chapters.

12.1 Additional View Options

The program window consists of different elements like the tool bars, the View area and as dock-able Windows the Repository Explorer and the Message Window.

Dock-able windows can be closed by clicking on the cross in the title bar. The Pin docks the window to be always visible or switch to an automatic mode where they are minimized and only appear if the cursor is over the window. Using the entries of the View Menu (Figure 43) these windows can be reopened.

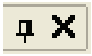

12.2 Help Menu

In the Help Menu you'll find helpful information about the actual software version running (Figure 212).

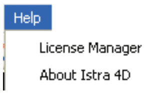

Figure 212 Menu Help

The first entry opens the *License Manager*. Here you can see and change the active license, delete and install additional licenses for more information see Chapter 1.2.

The function *About* opens a dialog displaying the actual version of software running and the active license information.

Below the version of the software information about the hardware used is shown. If the software version is not a rlease version the expiry date is displayed as well.

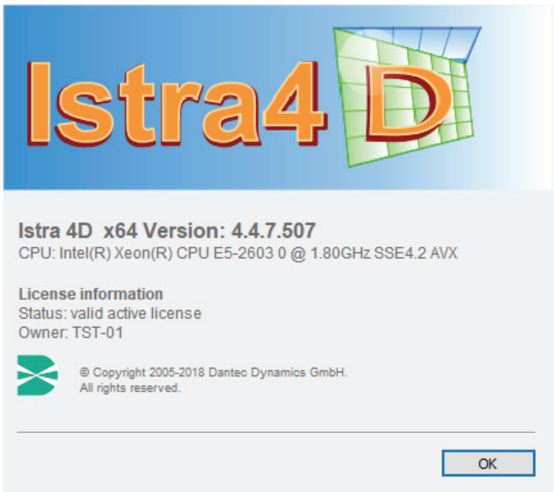

Figure 213 Dialog About

12.3 Samples of Recording Procedures

In this chapter presents a collection of different recording procedures as an example for the use of this features.

NOTE: *The source code of these procedures can be found with the program installation in the following directory:*

[Program files]\Istra 4D 4.4.7\templates\recording_procedures

The first procedure contains a single loop with one break and one item element (Figure 214). The item element saves each 10th and the break element stops the procedure after 100 saved images.

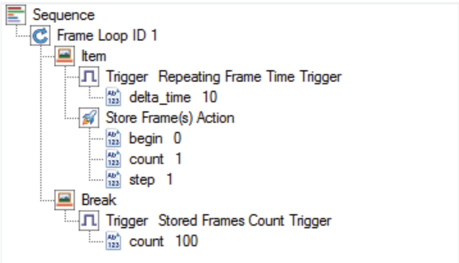

Figure 214 Recording Procedure Save a fixed number of images

The second procedure is an example how to increase the rate for saving images three times by an external trigger signal (Figure 215). It contains three loops and each loop is build from one break and one item element.

The first loop stores every 10th image on the hard disk. If the analog signal at channel ai0 exceeds 3 volts the next loop starts. Here every 5th image is saved until the signal at ai0 exceeds 3 volts again. The last loop saves every image from the stream until the third trigger is detected at channel ai0.

If for example the acquisition rate of the cameras is 10 Hz in the first loop every second (1Hz) an image is saved, in the second loop with 2Hz and in the last with 10Hz.

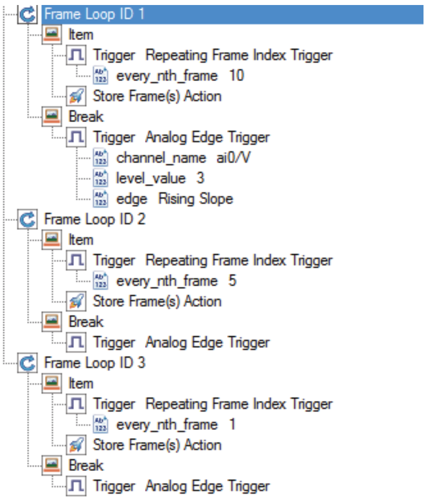

Figure 215 Recording Procedure Save images with increased speed

The third example demonstrates how to use the recording procedure for the definition of a post trigger measurement.

It might be used for a tensile test experiment where the point of failure is unknown. The goal is to acquire the time just before the failure at the maximum rate. In general the maximum frame rate from the cameras to the computer is too high to be save all images on the hard disk directly. That's why in this case the images at the beginning of the test are saved at a reduced rate. Just before the failure the images should be saved with the maximum frame rate of the cameras to the computer.

This procedure contains two loops (Figure 216). The first loop contains only one break element reacting on a rising voltage on channel named *"Start"*. This acts as a start trigger for the procedure.

The actual loop is the second one. It contains two item elements and one break element (this element is not shown in Figure 216). The first item element saves every 10th image (assuming that this rate is lower than the maximum storage rate). The second item element is triggered by a trigger signal on channel named *"Stop"* which indicates the failure of the sample. It saves 40 images before

this trigger at maximum frame rate of the cameras to the computer. The break element which is not shown exits the loop at the same trigger.

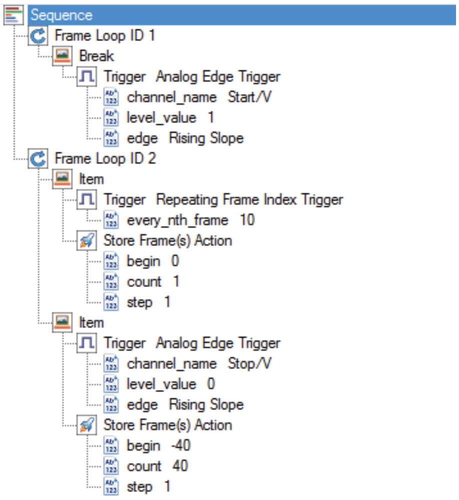

Figure 216 Recording Procedure Save images with a post trigger

The next example demonstrates how to use the recording procedure to perform a Phase Stepped Acquisition. Assuming the *DAQ master mode with external clock* is used and a proper signal is connected to the *Sync In* connector. The first loop sets the delay between the *Sync In* signal and acquisition signal of the camera to 0 degree and waits until the delay is equal to 0 degree (Figure 217).

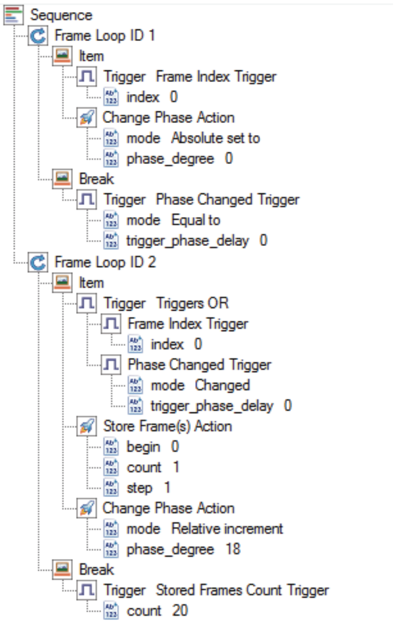

Figure 217 Recording Procedure Using Phase Stepped Acquisition

Within the second loop either at the first image or when the delay hasn’t changed one image is stored and the delay is changed by 18 degree. If 20 images are stored the procedure is finished.

Index

A
Acquisition
activation of hardware 7, 21
analog input 38
brightness setup 40
clock setup 35
default Projection Parameter 35
external clock 37
image format 35, 42
interval,see Camera
frame rate
load series 45
manual 45
phase stepped 54
pre-post trigger 46
Recording Procedure 49
region of interest (ROI) 35, 42
select hardware 21
settings 33
toolbar 28
trigger 46
ASCII, see Data
export
AVI, see Visualization
export movie
C
Calibration 56
2D calibration 77
automatic target detection 66
Baseline 77
Import Calibration Series 10
large field 68
Manual Projection 14
parameter 71
perform 73
Perpendicular Plane Mode 16
Position Marker Mode 17
prepare 63
QR Code 60
quality feedback 76
result 77
select target 65
separate for intrinsic and extrinsic 67
simultaneous 67
Stereo Angle 77
user defined targets 58
Camera
camera temperature 31
frame rate 36
shutter time 41
Coordinate system 128
dynamic 131
initial 128
static 131
user defined 129
Correlation
abort criteria 91
facet size 91, 101
grid spacing 92, 93
Real-Time see Real-Time Correlation
settings 89
D
Data
descriptions 164
export 156
HDF5 157
movie 156
properties 163
smoothing 156
smoothing globally 115
smoothing locally 115
unit system 160
E
Epipolar Line 103
Evaluation 78, 176
2D, 3D 80
create new 78
reference step 85
Universal Correlation Evaluation 79
Evaluation, see also Universal Correlation Evaluation
G
Gauge
circle 118

elements 116
export data 122
extensometer 120
labels 121
line 117
point 117
polygon 119
Show Value in 2D Visualization 126
show/hide elements 133, 171
spatial plot 123
spectral (FFT) 126
style 170
temporal plot 123
tree 120
H
Hardware for acquisition, see Acquisition
HDF5, see Data
HDF5
I
Images
export 108, 154
Import 7
movie 108
visualization 105
Import
Calibration Series 10
External Channels 174
Measurement Series 8
L
License 1
change 1
install 2
List
Select Steps 85
Live Image
color table 31, 33
toolbar 31
M
Mask
change 98
icon bar 97
save/load 99
Modules
set extension path 172
start 133
working directory 133
Multi Camera
Alignment 92
Evaluation 92
Visualization 137
O
Option
gauge style 170
icon size 162
mask editor 167
plot style 170
step for vector display 169
visualization 168
P
Parametrization 93
Pattern Quality 152
Projection Parameter 84
visualization 88
R
Real-Time Correlation 147
Analog Output 151
positioning assistance 145
start 148
visualization 149
Recording Procedure
break element 50
change phase action 54
edit procedure 55
examples 174
image feature 50
item element 52
storage frame action 54
Repository 3
activation 3
change 3
elements 4
S
see also Startpoint search
Shortcuts 161
Software version 173
Spatial 123
Startpoint search
add/delete 102
automatic mode 100
manual interaction 102
search radius 95
Status Bar 30
STL, see Data export
Strain
definition 112
Engineering (Eng.) 112
Lagrange (Lgr.) 112
sets 112
True (True) 112
U
Units
angle 160
displacement 160
gauge elements 110
options 160
phase 160

position 160
strain 160
time 160
Universal Correlation Evaluation
control panel 83
correlation settings 89
input data selection 84
mask definition, see also Mask
reference step 85
rung step 85
startpoint search 99
V
View
Full Screen 30
Visualization
2D graphic 108
2D Show Min Max Position 114
3D graphic 134
background color 168
control panel 108
coordinate system see Coordinate system 128
data 108
evaluated data 108
export movie 108, 156
facet size 101
Gauge spectral (FFT) 126
Gauge Value in 2D Visualization 126
grid spacing 93
images,see Images
visualization
line plots, see Gauge 123
measured data 105
movie 108
multi image control view 104
Orthographic projections 135
Real-Time see Real-Time Correlation visualization
smoothing,see Data
smoothing
step selection 108
strain 112
unit system, see Data unit system

List of Figures

Figure 1 Dialog License Explorer . . . 2
Figure 2 Change Repository . . . 3
Figure 3 Dialog Change Repository . . . 4
Figure 4 Repository Explorer . . . 4
Figure 5 Repository Explorer Tool Tip . . . 5
Figure 6 Repository Explorer Modify . . . 5
Figure 7 Transfer Evaluation Settings . . . 6
Figure 8 File-Import Series . . . 7
Figure 9 Dialog Import Series Select Cameras . . . 8
Figure 10 Dialog Import Series Select Name . . . 9
Figure 11 Dialog Import Series Progress . . . 10
Figure 12 Dialog Import Series Calculate Projection . . . 11
Figure 13 Display of imported images in BW (left) and Seg Line mode (right) . . . 12
Figure 14 Import Series Projection Calculation Extract Markers . . . 12
Figure 15 Import Series Projection Calculation Check Connectivity . . . 13
Figure 16 Import Series Projection Calculation Result . . . 14
Figure 17 Dialog Define Marker Positions . . . 15
Figure 18 Definition of the position of the line . . . 16
Figure 19 Definition of distance between two points . . . 16
Figure 20 Position of marker in the image . . . 17
Figure 21 Position of marker in the image (zoomed) . . . 17
Figure 22 Dialog Entering Marker Coordinates . . . 17
Figure 23 Menu Integrate External Channels Set . . . 18
Figure 24 Dialog Select Time Channel . . . 19
Figure 25 Integrated Channels Sets . . . 19
Figure 26 Menu Set Time Offset . . . 19
Figure 27 Temporal Plot Integrated External Channel (blue) and evaluated data (green). 20
Figure 28 Temporal Plot Integrated External Channel (blue) and evaluated data (green) with optimized Time Offset. 20
Figure 29 Hardware Activate . . . 21
Figure 30 Dialog Select Hardware . . . 21
Figure 31 Dialog Import Hardware Configuration . . . 22
Figure 32 Dialog Select Hardware Configuration Template . . . 23
Figure 33 Dialog Edit Hardware Configuration . . . 23
Figure 34 Menu Edit Configuration Files . . . 24
Figure 35 Edit Electronics Configuration File FireWire . . . 24
Figure 36 Edit SensorConfiguration File FireWire . . . 25
Figure 37 Edit Electronics Configuration File GigE . . . 26
Figure 38 Edit Sensor Configuration File GigE . . . 27
Figure 39 Acquisition Menu . . . 28
Figure 40 Select Measurement Configuration . . . 28
Figure 41 Acquisition Toolbar . . . 28
Figure 42 Live Image View . . . 29
Figure 43 Arrange Windows . . . 30
Figure 44 Status Bar . . . 30
Figure 45 Camera Temperature Status . . . 31
Figure 46 Acquisition Toolbar . . . 31
Figure 47 Image Display Toolbar . . . 31

Figure 48	Acquisition Settings Toolbar	33
Figure 49	Acquisition Correlation Menu	34
Figure 50	Acquisition Measurement Configuration	35
Figure 51	Select Acquisition Clock Dialog DAQ with internal clock	36
Figure 52	Select Acquisition Clock Dialog DAQ with external clock	37
Figure 53	Select Analog Input Dialog	38
Figure 54	Live Image with Analog Channel	39
Figure 55	Brightness Setup Dialog	40
Figure 56	Brightness Setup Dialog	41
Figure 57	Select Image Format Dialog	42
Figure 58	Definition of Region of Interest Dialog	42
Figure 59	Select Projection Parameter Dialog	44
Figure 60	Select Acquisition Type Dialog	45
Figure 61	Continue Recording Dialog	45
Figure 62	Image Acquisition toolbar	45
Figure 63	Dialog Pre-Post Trigger Settings	47
Figure 64	Video Display Toolbar	47
Figure 65	Dialog Save Images from the Buffer	49
Figure 66	Acquisition Menu Recording Procedure	50
Figure 67	Acquisition Menu Recording Procedure	52
Figure 68	Recording Procedure Add a Break Element	52
Figure 69	Acquisition Menu Recording Procedure	53
Figure 70	Default Recording Sequence Procedure	55
Figure 71	Dialog Select Target Specification	57
Figure 72	Dialog Create Target ID	58
Figure 73	Dialog Target Specification New	59
Figure 74	Dialog Create Target Images	59
Figure 75	Example of User Defined Calibration Target	60
Figure 76	Copy Target ID via Clipboard	61
Figure 77	Calibration Live Image	61
Figure 78	Acquisition Calibration Menu	62
Figure 79	Select Calibration Target Dialog	65
Figure 80	Selected Calibration Target	66
Figure 81	Calibration Target with detected QR code	66
Figure 82	Calibration Mode Selection	67
Figure 83	Calibration Set Points	69
Figure 84	Calibration Determine Camera Position	69
Figure 85	Calibration Pose Dialog	70
Figure 86	Calibration Pose Dialog Input	70
Figure 87	Calibration Pose Dialog Result	71
Figure 88	Calibration Settings Dialog	71
Figure 89	Calibration Process Toolbar	73
Figure 90	Calibration Acquisition of images	74
Figure 91	Calibration Display Mode Selection	75
Figure 92	Calibration display mode Lines and Black and White	75
Figure 93	Calibration Parameters calculated during the calibration process	76
Figure 94	Save Projection Parameters Dialog	77
Figure 95	Dialog New Evaluation	78
Figure 96	New Evaluation Select Projection Parameter	79
Figure 97	New Evaluation Select Camera Configuration	80
Figure 98	New Evaluation Settings Transfer	81
Figure 99	Control panel for Universal Evaluation	82
Figure 100	Controls for Universal Evaluations	82
Figure 101	Controls for Universal Evaluation (minimized)	82
Figure 102	Universal Correlation Evaluation Control	83
Figure 103	Universal Correlation Evaluation Input Data Selection Control	84
Figure 104	Menu Type of Evaluation Steps	85
Figure 105	Dialog Recalibration Results	86

Figure 106	Dialog Add Evaluation Step	87
Figure 107	Dialog Projection Parameter	88
Figure 108	3D Setup of cameras and calibration target	88
Figure 109	Universal Correlation Evaluation Settings Control	89
Figure 110	Parametrization showing grid points and facets	94
Figure 111	Parametrization showing grid points and facets (zoomed in)	94
Figure 112	Universal Correlation Evaluation Advanced Settings	95
Figure 113	Universal Correlation Evaluation Mask Control	97
Figure 114	Universal Evaluation Startpoints	100
Figure 115	Startpoint Show Facet Size	101
Figure 116	Image of the reference camera with mask area.	101
Figure 117	Startpoint Selection Menu	102
Figure 118	Startpoint search reset specified steps	102
Figure 119	Startpoint search Show Epipolar Line	103
Figure 120	Control window with image list	104
Figure 121	Visualization of acquired images	105
Figure 122	Menu View - In Image Info Settings	106
Figure 123	In Image Info Settings selection of area	106
Figure 124	In Image Info Settings insert new information	107
Figure 125	In Image Info Settings top area	107
Figure 126	Default Visualization	108
Figure 127	Default Visualization step selection control	109
Figure 128	Visualization 2D display	109
Figure 129	Visualization Graphics	110
Figure 130	Visualization 2D Color Overlay	113
Figure 131	Visualization 2D Color Overlay with MinMax display	113
Figure 132	Visualization 2D Grid Display	114
Figure 133	Visualization 2D Quiver Display	115
Figure 134	Visualization Smoothing	115
Figure 135	Visualization Gauge	116
Figure 136	Visualization Gauge New Point	117
Figure 137	Visualization Gauge New Line	118
Figure 138	Visualization Gauge New Circle	118
Figure 139	Visualization Gauge New Polygon	119
Figure 140	Visualization Gauge Point, Line Polygon Element	120
Figure 141	Visualization Gauge Extensometer	121
Figure 142	Visualization Gauge Set Extensometer Length	121
Figure 143	Visualization Gauge Rename Elements	121
Figure 144	Visualization of Gauge Elements with labels	122
Figure 145	Visualization Gauge Report Entry	122
Figure 146	Visualization Gauge Spatial Plot	123
Figure 147	Visualization Gauge Temporal Plot	123
Figure 148	Temporal Plot over step number or time	124
Figure 149	Temporal Plot two lines	124
Figure 150	Visualization Gauge Export Context Menu	125
Figure 151	Gauge ASCII Export Wizard File Name	127
Figure 152	Gauge ASCII Export Wizard Step Selection	127
Figure 153	Gauge ASCII Export Wizard Separator	128
Figure 154	Visualization Coordinate Systems	128
Figure 155	Dialog Create New Coordinate System	129
Figure 156	Visualization control panel Coordinates 2 Points Method	130
Figure 157	Visualization control panel Coordinates Step Selection	130
Figure 158	Visualization control panel Coordinates Scalar Input	131
Figure 159	Stencil mode with display of data points	132
Figure 160	Stencil mode marking points (left area, right individual)	132
Figure 161	Stencil mode marked points (left stencil mode, right graphics mode)	133
Figure 162	Visualization control panel Modules	133
Figure 163	3D Visualization Contour and Distance to Bestfit Plane	134

Figure 164 3D Visualization Change color scaling by mouse 135
Figure 165 3D Visualization Orthographic Projections XY plane 136
Figure 166 3D Visualization Orthographic Projections XZ plane (left, top view) and YZ (right, side view) plane. 136
Figure 167 Available Cylinder Coordinate displacements 137
Figure 168 Comparison between Cartesian and Cylinder Coordinate System 2D . . . 138
Figure 169 Comparison between Cartesian and Cylinder Coordinate System 3D . . . 138
Figure 170 2D Visualization of a Bi-Plane object overlay 139
Figure 171 Visualization Bi-Plane Point 140
Figure 172 3D Visualization Global Gauge Strain temporal plot 140
Figure 173 Spatial plot of the thickness change at single step 141
Figure 174 Spatial plot of the thickness strain at single step. 141
Figure 175 3D Visualization Bi-Plane 141
Figure 176 3D Visualization strain X, strain Y and strain Z 142
Figure 177 Live Display Camera Images 144
Figure 178 Live Display Control Panel 144
Figure 179 Live Display Positioning Control 145
Figure 180 Live Image Positioning Parameter 146
Figure 181 Live Image Real Time Correlation. 148
Figure 182 Live Image Real Time Correlation Connect 148
Figure 183 Live Image Real Time Correlation Connection List 149
Figure 184 Live Image Real Time Correlation Graphics 150
Figure 185 Live Image Real Time Correlation Smoothing. 151
Figure 186 Live Image Real Time Correlation Analog Output. 152
Figure 187 Live Image with Pattern Quality Overlay 153
Figure 188 Export Image Series 154
Figure 189 AVI File Configuration Dialog 155
Figure 190 File Export Menu 156
Figure 191 Dialog Select Data Export Format STL 157
Figure 192 Dialog Select Data Export Format HDF5 157
Figure 193 Dialog Select Data Export Format Step Selection. 158
Figure 194 Dialog Select Data Export Format ASCII 159
Figure 195 Dialog Options Environment Units 160
Figure 196 Dialog Options Environment Keyboard 161
Figure 197 Dialog Options Environment UI 162
Figure 198 Dialog Options Environment Cache 162
Figure 199 Dialog Options Properties 163
Figure 200 Dialog Change Properties 164
Figure 201 Dialog Options Description 165
Figure 202 Dialog Insert Properties. 165
Figure 203 Description Allocation Dialog. 166
Figure 204 Options Select Mask and Start Point color 167
Figure 205 Dialog Options Acquisition Recording 168
Figure 206 Dialog Options Visualization Appearance 169
Figure 207 Dialog Options Visualization Vector Data Style. 170
Figure 208 Dialog Options Visualization Plot Style 170
Figure 209 Dialog Options Visualization Gauge Style. 171
Figure 210 Dialog Options Visualization Show/Hide 171
Figure 211 Dialog Options Extensions Path 172
Figure 212 Menu Help 173
Figure 213 Dialog About 174
Figure 214 Recording Procedure Save a fixed number of images 174
Figure 215 Recording Procedure Save images with increased speed 175
Figure 216 Recording Procedure Save images with a post trigger 176
Figure 217 Recording Procedure Using Phase Stepped Acquisition 176

List of Tables

Table 1:	Repository Icons	4
Table 2:	Acquisition Mode Icons	29
Table 3:	Image Display Toolbar	32
Table 4:	Acquisition Settings Icons	34
Table 5:	Acquisition Settings Menu	35
Table 6:	Acquisition Icons	46
Table 7:	Video Display Icons	48
Table 8:	Acquisition Calibration Settings Menu	62
Table 9:	Indication of found corners during on-line calibration	63
Table 10:	Calibration Process Icons	73
Table 11:	Calibration Status Indication	74
Table 12:	Control Mask Edit Icons	97
Table 13:	Control Mask Change Elements	98
Table 14:	Gauge Data Export	125
Table 15:	Orthographic Projections Control Elements	135
